# NONO regulates m^5^C modification and alternative splicing of PTEN mRNAs to drive gastric cancer progression

**DOI:** 10.1186/s13046-024-03260-z

**Published:** 2025-03-04

**Authors:** Gaichao Zhao, Ruochen liu, Lingjun Ge, Dan Qi, Qishu Wu, Zini Lin, Houji Song, Liping Zhong, Hongjuan Cui

**Affiliations:** 1 State Key Laboratory of Resource Insects, Medical Research Institute, Southwest University, Chongqing, 400716 China; 2 Chongqing Engineering and Technology Research Center for Silk Biomaterials and Regenerative Medicine, Chongqing, 400716 China; 3 Engineering Research Center for Cancer Biomedical and Translational Medicine, Southwest University, Chongqing, 400716 China; 4 Jinfeng Laboratory, Chongqing, 401329 China; 5 State Key Laboratory of Targeting Oncology, Guangxi Medical University, Nanning, 530021 Guangxi China

**Keywords:** NONO, Alternative splicing, m^5^C, NSUN2, PTEN, GC

## Abstract

**Background:**

The effect of m^5^C modification on oncogene mRNAs has been well studied, while little is known about its influence on mRNAs of tumor suppressor genes (TSGs). Early studies showed *PTEN*, a key TSG, undergoes alternative splicing (AS) in cancers, however, the underlying regulatory mechanism remains elusive.

**Methods:**

We analyzed tissue microarrays and transcriptomic data derived from gastric cancer, with an emphasis on RNA splicing and m^5^C regulators. To unravel the role of NONO in GC, we employed RNA sequencing, RNA-Bis-Seq, RNA immunoprecipitation, RNA in situ hybridization, and Minigene reporter assay with NONO knockdown cells. The clinical relevance was validated using CDX models and human tissue microarrays.

**Results:**

Analysis of publicly available datasets and immunohistochemistry assay of tissue microarrays containing 40 GC tissues showed NONO was upregulated in GC and contributed to poor prognosis. In vitro and in vivo experiments indicated a positive regulatory role of NONO in terms of cell proliferation, migration, and invasion of GC. Mechanically, NONO interacted directly with PTEN pre-mRNA and recruited the RNA m^5^C methyltransferase NSUN2 via RNA-recognition motif (RRM) domains, altering the mRNA methylation pattern across PTEN pre-mRNA. The oncogenic role of NONO/NSUN2/PTEN axis in GC progression was further confirmed with pre-clinical experiments and clinical data.

**Conclusion:**

Here, we revealed NONO-regulated AS of PTEN mRNA in an m^5^C-dependent manner, resulting in the downregulation of PTEN expression in gastric cancer (GC).This study unveils a novel regulatory mechanism of tumor suppressor gene inactivation mediated by m^5^C modification and related alternative splicing in cancer.

**Supplementary Information:**

The online version contains supplementary material available at 10.1186/s13046-024-03260-z.

## Introduction

RNA modifications, such as N6-methyladenosine (m^6^A) and 5-methylcytosine (m^5^C), play an important role in gene regulation [1, 2]. Dynamic RNA modifications have been shown to regulate diverse cellular functions under physiological and/or pathological conditions. Recently, m^5^C has been detected not only in ribosomal RNAs and transfer RNAs, but also messenger RNAs (mRNAs) by high-throughput sequencing-based transcriptome-wide mapping approaches [3]. The m^5^C modification in mRNAs is mainly catalyzed by NOP2/Sun RNA methyltransferase family member 2 (NSUN2) and displays a preferential accumulation in the vicinity of the translational start sites, 3′ untranslated regions (UTRs), and the Argonaute-binding regions in mRNAs, regulating the fate of many mRNAs of oncogenes [4, 5]. NSUN2-catalyzed mRNA methylation of TREX2, TIAM2, PFAS, and ORAI2 promotes the oncoprotein expression and tumor progression, mainly through enhancing the mRNA stability of these oncogenes [6–9]. However, little is known about the influence of m^5^C on tumor suppressor gene (TSG) mRNAs and the regulation mechanism of m^5^C deposition.

PTEN is a critical TSG and the main negative regulator of the PI3K pathway which is one of the main signaling cues that drive tumor progression. Various mechanisms at transcriptional, translational, and posttranslational levels control the expression of PTEN. Alternative splicing (AS) of the PTEN transcripts has long been suggested as an important aspect of PTEN regulation and biology [10, 11]. Two different PTEN splicing variants (SVs) were first identified in glioblastoma and prostate cancer cells [12]. At least eight additional PTEN SVs have been identified and found to be differentially expressed in breast cancer, including SVs with intron 3 (transcripts 3 A, 3B, and 3 C indicate different resident fragments of the intron 3) and 5 (transcripts 5 A, 5B, and 5 C indicate different resident fragments of the intron 5) in the mature transcripts, and SVs with partial or complete deletion of exon 5 (DelE5) or exon 6 (DelE6), respectively [13]. However, the regulation mechanism of PTEN AS remains elusive.

In this study, NONO was found to regulate AS of PTEN mRNAs in an m^5^C-dependent manner, resulting in the downregulation of PTEN expression in gastric cancer (GC). NONO was upregulated in GC and contributed to poor prognosis, and its positive roles in cell proliferation, migration, and invasion of GC were confirmed in vitro and in vivo. Mechanically, NONO interacted directly with PTEN pre-mRNA and recruited the RNA m^5^C methyltransferase NSUN2 via RNA-recognition motif (RRM) domains, altering the mRNA methylation pattern across PTEN mRNAs. The oncogenic role of the NONO/NSUN2/PTEN axis in GC progression was further confirmed with pre-clinical experiments and clinical data. Therefore, this study revealed a novel regulatory mechanism of TSG inactivation mediated by m^5^C methylation and alternative splicing in cancer.

## Materials and methods

Human GC cell lines (MKN-45, HGC-27, SGC-7901, BGC-823, and MGC-803) and Human Gastric Epithelial Cells (GES-1) were purchased from ATCC. Human-derived gastric cancer cell (GC-1) were purchased from Pricella. The cells were cultured in RPMI-1640 Medium (RPMI-1640; 01-100-1, Bio Ind) supplemented with 10% fetal bovine serum (FBS; Gibco).

The full-length (FL) sequences of NONO and mutants of NONO (ΔRRM1, ΔRRM2, ΔRRM1/2, ΔNOPS, ΔCC) were subcloned into a pCDH-CMV-MCS-EF1-Puro vector. All plasmids were validated by DNA sequencing. The short-hairpin RNA (shRNA) oligonucleotides against NONO and NSUN2 were synthesized by Sangon Biotech (Shanghai, China), which are listed in Supplementary Table S3. According to the manufacturer’s instructions, transfection was performed using Lipofectamine 2000 reagent (Invitrogen Life Technologies^®^, Carlsbad, CA, USA).

NONO-knockdown GC cell lines were constructed using the Lentivirus transfection system. Lentivirus infection was performed on GC cells at 30% confluency, infected twice, 24 h each time. The cells were selected after culture for one week in a medium containing 2 µg/ml puromycin (MCE, Shanghai, China). Monocolonies were picked, and the knockout efficiency was determined by western blotting.

### Animal experiments

Four-week-old female NOD/SCID nude mice were injected subcutaneously with 1×10^7^ GC cells infected with various lentiviruses. All processes were executed according to NIH Guidelines for the Care and Use of Laboratory Animals and approved by Southwest University Animal Care and Use Committee (IACUC-20231109-01). The width and length of the tumor were measured once every week, and tumor volume was calculated based on the formula (length × width^2^/2). Mice were sacrificed and tumors were harvested and weighed after 4 weeks. The tumors and lungs were obtained for the following study. GC cells were stably infected with short hairpin control (shGFP), and shNONO#1. 4 × 10^6^ cells were injected into female NOD/SCID nude mice tail veins. Mice were sacrificed when they began to show symptoms of continuous discomfort. Lungs were collected and fixed in 4% formaldehyde for HE staining and IHC analysis.

### Construct

A lentiviral plasmid encoding FLAG-NONO and HA-NSUN2 were constructed by cloning the coding region of NONO or NSUN2 into pCDH-CMV-MCS-EF1-Puro vector (YouBio) using the ClonExpress II One Step Cloning Kit (Vazyme). FLAG-NONO-(ΔRRM1, ΔRRM2, ΔRRM1/2, ΔNOPS, ΔCC) constructs were generated by cloning the coding region of corresponding the truncated mutants of NONO into pCDH-CMV-MCS-EF1-Puro vector (YouBio) using the ClonExpress II One Step Cloning Kit (Vazyme). Prokaryotic expression recombinant plasmid HA-NSUN2 and FLAG-NONO-FL/S were constructed into p-GEX-4T-1 (YouBio).

The PTEN minigene reporter was generated by amplifying genomic segments including the PTEN exon 5, intron 5, and exon 6, and ligating into restriction enzyme-linearized pMini-CopGFP vector using ClonExpress II One Step Cloning Kit (Vazyme). Mutations (the truncated mutants of NONO/NSUN2 binding sites) were introduced into PTEN minigene using ClonExpress II One Step Cloning Kit (Vazyme).

### Immunohistochemistry (IHC)

The expression level of NONO in tumor and normal tissues was determined using IHC on a tissue microarray (TMA), constructed as previously described [14, 15]. Briefly, after paraffin-embedded tissue sections, dewaxing, and hydration, the sections were placed in a citric acid buffer (pH = 6.0) for antigen retrieval. Then, removing endogenous peroxidase, the sections were blocked with 10% BSA at 37 °C for 50 min. After the incubation with primary antibodies (NONO: ab70335, Abcam; NSUN2: 20854-1-AP, Proteintech; PTEN: ab267787, Abcam; Ki67: 27309-1-AP, Proteintech; m^5^C: 68301-1-Ig, Proteintech) at 4 °C overnight. The sections were incubated with a secondary antibody at 37 °C for 1 h. The immunoreactive signals were performed with DAB staining (ZLI-9018, ZSGB-BIO, Beijing, China). Nuclei were stained with hematoxylin (Beyotime, China). For m^5^C detection, samples were pretreated with DNase before antigen retrieval.

The H score was determined based on the intensity of staining and the proportion of labeled tumor cells as previously described [16]. A semi-quantitative analysis of the sample tissue sections was performed to score for IHC staining. At least 5 fields of view were randomly selected for each section, and the positive expression was judged as weak expression (+, 1 point), medium expression (++, 2 points), and strong expression (+++, 3 points) according to the degree of positive expression. Combined with the proportion of positive expression, the pathological score was finally obtained by “H-Score”. The formula where *pi* is the percentage of positive cells, and *i* is the staining intensity.

$$\begin{aligned}\text{H-Score} &= \sum (pi\times i)\cr&= (\% \text{ of weak int ensity cells }\times 1) \cr&\quad+ (\% \text{ of mod erate int ensity cells}\times 2) \cr&\quad+ (\% \text{ of strong int ensity cells} \times 3)\end{aligned}$$

### Immunofluorescence

After transfection, the cells grown on the coverslips were rinsed twice with PBS and fixed with 4% paraformaldehyde in PBS for 10 min on ice. After washing three times with PBS, cells were permeabilized with 0.3% TritonX-100 in PBS for 10 min on ice. Cells were then washed twice with PBS, blocked with 5% goat serum in PBS for 30 min at 37 °C. and incubated overnight at 37 °C with primary antibodies at the dilution ratio as indicated. After washing with PBST, cells were incubated with Alexa Fluor 488 and Alexa Fluor 594 goat anti-rabbit/mouse secondary antibodies for 2 h at 37 °C. Afterward, the cells were incubated with 4,6-diamidino-2-phenylindole (DAPI) for 15 min. Fluorescent images were acquired using a confocal fluorescence microscope (Olympus Fv1000, Japan). The relative mean fluorescence densities were analyzed by Image-Pro Plus and plotted using GraphPad Prism 9 software.

### Co-immunoprecipitation (Co-IP)

For Co-IP assays, cells transfected with Flag-tagged NONO were lysed with Cell lysis buffer for IP-containing protease-inhibitor Phenylmethanesulfonyl fluoride (PMSF). The supernatant was collected and incubated with anti-NONO magnetic beads (Thermo Fisher Scientific Inc.) for at least 4 h at 4 °C. The immunoprecipitates were then boiled in 2× SDS loading buffer and analyzed by SDS-PAGE.

### GST pull-down assay

The plasmids for GST-Flag-NONO and GST-HA-NSUN2 were transfected into E. coli. The fusion proteins were prepared as described previously. Approximately 100 µg of GST-Flag-NONO and HA-NSUN2 fusion protein were incubated overnight with an anti-Flag antibody at 4 ℃ with shaking. The immune complex solution was incubated with protein A/G magnetic beads for 1 h at room temperature with mixing and then washed to remove the unbound immune complex. The bound immune complex was dissociated from the beads with low-pH buffer for western blotting analysis.

### Extraction of cytoplasmic and nuclear lysates

Cytoplasmic and nuclear lysates were extracted to assess the localization of NONO using a Nuclear and Cytoplasmic Protein Extraction Kit (P0027, beyotime) as previously described [17]. Briefly, cells were lysed on ice using a cold cell fractionation buffer for 10 min and centrifuged at 13,000 × g for 5 min to obtain the cytoplasmic fraction. The precipitate was then lysed using a cell-disruption buffer to obtain the nuclear protein.

### Western blotting

Cells were harvested and dissolved in RIPA lysis buffer and a protease-inhibitor cocktail. Whole-cell lysates were subjected to SDS-PAGE and transferred to PVDF membranes (Bio-Rad, Hercules, CA, USA). After blocking and incubating with specific primary and secondary antibodies, the proteins on the membranes were visualized using the Bio-Rad ChemiDoc^®^ Touch Imaging System (Bio-Rad). The antibodies used are listed in Supplementary Table S3.

### RNA isolation and quantitative reverse-transcription polymerase chain reaction (qRT-PCR)

Total RNA was extracted from the GC cell lines using TRIzol reagent (TAKARA), and reverse-transcribed using a Hifair^®^ III 1st Strand cDNA Synthesis SuperMix for qPCR (gDNA digester plus) (yeasen, Shanghai, China) according to the manufacturer’s instructions. The complementary DNA was then amplified to detect genes by qRT-PCR using a SYBR Green Master mix (QIAGEN) according to the manufacturer’s protocol. All tests were repeated independently three times. The mRNA expression was normalized to the expression of GAPDH mRNA and calculated using the 2^−ΔΔCt^ method. The PCR primers used are listed in Supplementary Table S4.

### Cell proliferation and colony-formation assays

Cell proliferation was assessed using Methylthiazolyldiphenyl -tetrazolium bromide (MTT, Beyotime, China). Briefly, cells were seeded into 96-well plates (2000 cells/well, five replicates). Then, cells were transfected and treated with MTT regent. The OD value at 560 nm was measured using a microplate reader. For colony formation assays, transfected cells were placed in six-well plates (1000 cells/well) for two weeks. Cells were then fixed with 4% paraformaldehyde for 10 min and stained with 0.1% crystal violet for 10 min. The number of colonies was counted.

### EdU assay

The effect of NONO on GC cell proliferation was also tested using an EdU(5-Ethynyl-2′-deoxyuridine) assay kit (BeyoClick™ EdU Cell Proliferation Kit with Alexa Fluor 594, Beyotime, China). Briefly, 10,000 cells per well were added to 96-well plates (three replicates). After transfection, EdU (10 µM) was added and incubated at 37 °C for 2 h. Subsequently, the cells were washed with PBS and fixed with 4% paraformaldehyde for 30 min. Next, 0.1% Triton X-100–PBS was used for cell membrane permeabilization for 10 min. After washing with PBS three times, cells were stained with EdU Alexa Fluor 594 for 30 min, and DNA was stained with Hoechst-33,342 for 20 min. The proportion of EdU-positive cells was visualized by fluorescence microscopy.

### Transwell migration and invasion assays

Transwell migration and invasion assays were performed as described previously [17, 18]. For the invasion assay, the chambers were coated in advance with Matrigel (BD Pharmingen, San Jose, CA, USA). Then 1 × 10^5^ transfected cells were seeded into the upper chamber in a serum-free medium, and a medium with 10% FBS was added to the basolateral chamber. After 24-h incubation for the migration assay and 36-h incubation for the invasion assay, cells were fixed with 4% paraformaldehyde for 10 min and stained with 0.1% crystal violet for 10 min. The cells were photographed under a microscope (Leica, London, UK) in five randomly selected visual fields.

### RNA-Seq analysis

Total RNA was extracted from cells (shNONO cells and shGFP MKN-45 cells, three biological replicates) using TRIzol (Invitrogen) and subjected to RNA-Seq, according to the manufacturer’s instructions. The sequencing library was built and sequenced with mRNA-Seq kit of Illumina by Sangon Biotech (Shanghai, China). In brief, PolyA + RNA was purified from 100 ng of total RNA with oligo-dT beads, and then fragmented with divalent cations under elevated temperature. First strand synthesis was performed with random hexamer and reverse transcriptase, and second strand synthesis with RNAseH and DNA PolI. Following cDNA synthesis, the double stranded products were end-repaired, a single “A” was added and then the Illumina PE adaptors were ligated on to the cDNA products. The ligation products were purified using gel electrophoresis. The target size range for these libraries was 200 ~ 300 bp, such that the final library for sequencing would have cDNA inserts with sizes of ~ 200 bp long. One run of 2 × 50 bp paired-end sequencing was performed on the HiSeq2500 instrument, using one lane per tissue, to produce approximately 80 million read pairs per cells (160 million sequences). The sequencing quality of RNA-Seq libraries was assessed by FastQC v0.11.2 (http://www.bioinformatics.babraham.ac.uk/projects/fastqc/). RNA-seq libraries were mapped to the human genome using HISAT2 (v2.1.0), and the mapped reads were then processed by StringTie to estimate the expression levels of all genes and identify differentially expressed genes. The expression level of a gene is expressed as a gene-level fragments per kilobase of transcripts per million mapped reads (FPKM) value. Since there were only 3 replicates in each group, upregulated or downregulated genes in shNONO groups were identified according to ≥ 2-fold expression changes. ASprofile [19] was used to analyze the different AS events between the control and shNONO groups.

### RNA-Bis-seq and bioinformatics analyses

The RNA-BS libraries were constructed as follows. RNA fragmentation and bisulfite conversion were performed as previously described [20] with some modifications. In brief, total RNA was isolated from cultured cells (shNONO MKN-45 cells and shGFP MKN-45 cells, two biological replicates) using TRIzol (Invitrogen). The extracted total RNA was firstly subjected to DNase I (NEB) treatment at 37℃ for 30 min to remove residual DNA. After purification, mRNA was enriched using Dynabeads™ mRNA Purification Kit (Thermo Fisher Scientific) according to the manufacturer’s instructions.The purified mRNA was further treated using Ribominus Transcriptome Isolation Kit (Invitrogen) to depletion rRNA. 200 ng mRNA along with 1 ng in-vitro-transcribed mouse dihydrofolate reductase (Dhfr) spike-in RNA were used. RNA bisulfite conversion of the isolated mRNA samples and was conducted using EZ RNA Methylation Kit (Zymo Research) with 70 ℃ for 5 min followed by 54℃ for 45 min incubation and then purified using the provided Zymo-Spin IC Column according to the manufacturers’ instructions. Bisulfite converted RNA reverse transcription was carried out using ACT random hexamers and Superscript II Reverse Transcriptase (Invitrogen) according to the manufacturer’s instructions. The synthesized cDNA was amplified using STARmix Taq DNA Polymerase (GenStar) with the following program: 94 °C for 3 min; 25 cycles of 94 °C for 30 s, 52 °C for 30 s and 72 °C for 20 s; and 72 °C for 1 min. Then the PCR product used for the final library. After analyzed by Agilent 2100 Bioanalyzer (Agilent Technologies, USA) and quantified by real time PCR, RNA-BS libraries were finally sequenced using Illumina Novaseq 6000 platform (Illumina, USA).

Raw RNA-BisSeq reads for each sample were stripped of adaptor sequences and removed low-quality bases using Trimmomatic [21]. The processed reads with lengths greater than 20 nt were defined as clean reads. To ensure the sufficient conversion efficiency, Analysis the C to T conversion rates > 99%. Reads with > 30% unconverted cytosines that may reflect insufficient bisulfite conversion were eliminated [4]. Human reference genomes (GRCh38.p13) were downloaded from the Ensembl database. The alignment procedure was performed by mapping the clean reads against the GRCh38.p13 genome by BS-RNA V1.0 [22]. The unmapped reads were mapped against the transcriptome by BS-RNA with the same parameters. The remaining reads were further mapped to the library collecting all exon-exon junctions based on the Ensembl annotation.

The methylation level is estimated as *i* / (*i* + *j*) where *i* represents the number of reads showing methylation (C) at each m^5^C site, and j represents the number of reads lacking methylation (T). Differential m^5^C sites were defined with the following criteria: mean m^5^C level difference ≥ 0.1 (shNONO and shGFP cells), *p* < 0.05 (Wilcoxon test). For the hypermethylated m^5^C sites in shNONO cells, mean m^5^C level difference ≥ 0.1 and sites with *P* < 0.05 between the comparison groups (shGFP) were considered to be statistically significant.

### RNA in situ hybridization

GC cells grown on coverslips were fixed with 4% formaldehyde in PBS at room temperature for 20 min. Cells were then permeabilized with 0.5% TritonX-100 in PBS for 15 min, and washed twice with PBS. Hybridization was performed for 5 h at 37 °C in the mixture containing 20% formamide, 2× SSC, 1 mg/ml tRNA (Sigma), 10% dextransulfate. Cy3 labeled oligo(dT) 50 probes (Sangon Biotech) were used for analyzing endogenous mRNA export. Cy3-labeled oligonucleotide probes complementary to PTEN mRNAs were conducted to visualize the location of PTEN mRNA minigene. After washing three times with 2× SSC buffer and once with 1× SSC buffer, coverslips were then mounted with a DAPI-containing mounting medium (Vector Laboratories). Optical sections were captured with a confocal fluorescence microscope (Olympus Fv1000, Japan). The probes are listed in the Supplementary Table S6.

### RNA-stability assay

MKN-45 cells were transfected with shRNAs against NONO or controls in six-well plates. After shRNA transfection (24 h), the cells were treated with 5 µg/ml actinomycin D or DMSO and collected at the indicated time points(0 min, 20 min,40 min,60 min). The total RNA was extracted and analysed by quantitative real-time PCR. GAPDH was used as an internal control.

### Dot blot assay of m5C level

Dot blot assay of m^5^C level was carried out following previous reports [23]. Briefly, 1 µg total RNA from GC cell lines was dropped onto Hybond-N + membrane after denaturing at 95℃. Then the RNA was cross-linked to membrane by UV -light. After blocking by 5% of non-fat milk, the membrane was incubated with anti-m5C antibody (ABclonal Cat# A22404). Then the membrane was incubating with HRP-conjugated goat anti-rabbit IgG (Proteintech Cat# SA00001-2), and was visualized through Super ECL Plus detection reagent (Abbkine Cat# BUM102-CN) with a detection instrument (Tanon).

### RNA immunoprecipitation (RIP)

To examine the interaction of NONO protein and the target sequence, RNA immunoprecipitation was conducted and all steps were performed according to the manufacturer’s protocol (BerSinBio# Bes5101), with the following modifications. In short, 4 × 10^^7^ cells were washed with PBS and resuspended and lysised with polysome lysis buffer containing protease inhibitors and RNA enzyme inhibitors (polysome lysis buffer: protease inhibitors: RNA enzyme inhibitors = 1700:17:7.5); then, the DNA was removed using DNase. Purified recombinant Flag-NONO protein was added into cell lysate and divided into three groups (input, IP, and IgG groups). Anti-Flag antibodies (Abcam) and IgG antibodies (BerSinBio#Bes5101, 5 µg) were incubated with cell lysate and Flag-NONO protein mixture, respectively, at 4 °C for 16 h. Next, the RNA-protein complexes were isolated by incubating cell lysates mixture with the protein A/G magnetic beads at 4 °C for 4 h. After proteinase K digestion, protein-bound RNAs were extracted by phenol/chloroform/isoamyl alcohol (125:24:1) (Solarbio). The protein-bound RNAs were detected by qRT-PCR and assessed by %Input (%Input = 2^− [ΔCt IP−(ΔCt input − log2 Input Dilution Factor)]^, Input Dilution Factor = (Volume Input / Volume Input + Volume IP + Volume IgG)^−1^). The fold enrichment (fold enrichment = 2^− (ΔCt IP−ΔCt IgG)^) was also calculated to evaluate the alternations of RNA-protein binding caused by NONO knockdown. The primers used in this assay are listed in the Supplementary Table S7.

### RNA-protein pull-down experiments

#### In vitro transcription of PTEN RNA

The human PTEN gene encoding full-length (FL) PTEN mRNA was amplified by PCR and subcloned into the downstream of the T7 promoter which contains a T7 promoter sequence at its 5′ terminus. Purified T7- PTEN line plasmid was subjected to an in vitro transcription reaction with T7 RNA polymerase (Beyotime, China) at 37 °C for 4 h in a 100 µl reaction mixture, according to the manufacturer’s instructions. The primers used for cloning are listed in the Supplementary Table S8.

#### Pull-down with biotin-labeled mRNAs

RNA-protein pull-down experiment was performed by Pierce Magnetic RNA–Protein Pull Down Kit (Thermo Fisher Scientific, USA) following the manufacturer’s guideline. Briefly, NONO-overexpressing GC-1 cells were harvested and lysed in IP lysis buffer on ice for 30 min. The FL-PTEN mRNAs were labeled with biotin by Pierce™ RNA 3′ End Desthiobiotinylation Kit (Thermo Fisher Scientific). The cell lysates were incubated with biotin-labeled PTEN mRNA or control probes at 4 °C for 12 h to generate RNA-protein complexes. Streptavidin agarose magnetic beads were incubated with the RNA-protein mixture at 4 °C for 6 h to pull down RNA-protein complexes and flow-through (FT) was obtained. Then use the elution buffer (E) to collect the binding proteins. The binding proteins were identified by Western blot.

### Docking and molecular modeling

The computer-based virtual docking utilized the crystal structure based on NSUN2 (PDB: Q08J23) and NONO (PDB: 5IFM). Before proceeding with docking, pre-processing steps under the Chemical Computing Group Molecular Operating Environment included preparing the protein structures, performing protonation, assigning partial charges, and refining the energy of the system. For actual protein-protein docking, the Zdock server (https://zdock.umassmed.edu/) was utilized, and for the plotting of results, PyMOL 2.5 (https://pymol.org/2/) was used.

#### RNA-binding proteins site prediction

RBPsuite (http://www.csbio.sjtu.edu.cn/bioinf/RBPsuite/) was used to predict RBP binding sites on PTEN RNAs. (1) Choose human species; (2) Choose linear RNA type; (3) Choose general RBP model; (4) Input RNA sequence. When the job is finished, the prediction results will appear on the results page.

### Minigene reporter assay

To study the splicing of PTEN exon 5 following NONO knockdown, HGC-27 cells were co-transfected with minigene splicing reporter and shRNA oligos targeting the NONO (shNONO). The cells were harvested for RNA analyses 48 h after transfection.

### Statistical analysis

Statistical analyses were conducted by GraphPad Prism 9.0 (GraphPad Software Inc., CA, USA). Data are expressed as the mean ± S.D. (Standard Deviation). The comparisons of the two groups were computed using a two-tailed Student’s t-test, ANOVA or χ2 test. Correlations were analyzed by Pearson’s correlation test. Univariate and multivariate Cox proportional hazards regression models were used to identify factors associated with survival. The survival analysis was conducted with the Kaplan–Meier plots and the log-rank test. Differences were statistically significant at *p* < 0.05.

## Results

### NONO acts as an oncogene and PI3K-AKT signaling regulator in gastric cancer

**Fig. 1:**
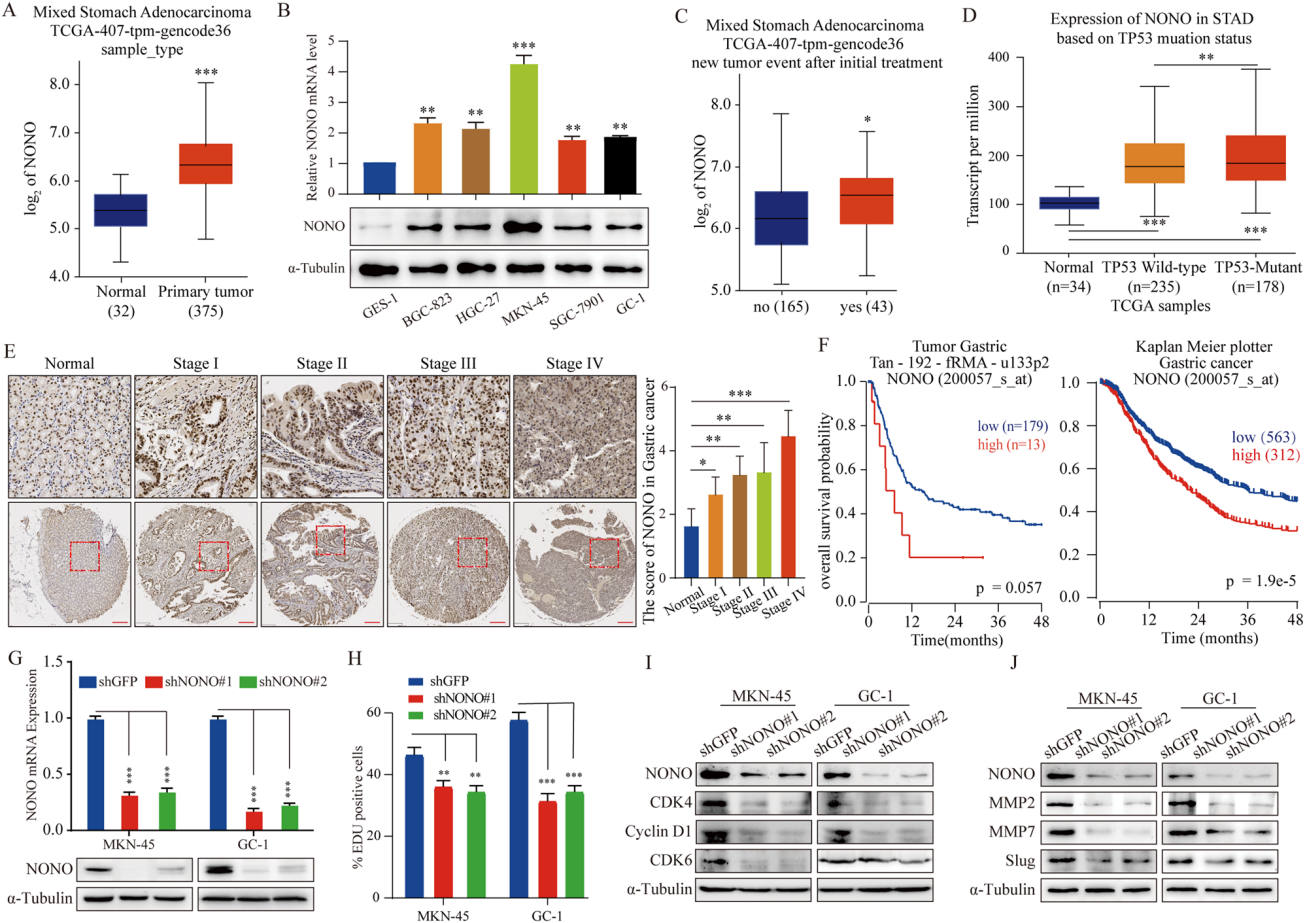
NONO acts as an oncogene and PI3K-AKT signaling regulator in gastric cancer . **(A)** The level of NONO mRNA was significantly increased from normal stomach tissues (32) to gastric cancer tissues (*n* = 375) in TCGA dataset and *p* values were indicated. **(B)** qRT-PCR and Western blot analysis of NONO mRNA expression in a non-cancer cell lines (GES-1), 4 GC cell lines (BGC-823, HGC-27, MKN-45 and SGC-7901) and a patient-derived primary GC cell (GC-1) (*n* = 3). **(C)** NONO mRNA expression was analyzed in gastric tissues (*n* = 208), with or without new tumor event after initial treatment. **(D)** NONO mRNA expression was analyzed in gastric tissues (*n* = 447) based on TP53 mutation status. **(E)** Representative images of immunohistochemical staining for NONO protein in a tissue microarray (scale bar = 200 μm). **(F)** The correlation between NONO expression levels and survival rate were obtained from Tumor Gastric Tan-192-fRMA-u133p2 and Kaplan-Meier Plotter and were performed through Kaplan–Meier (K–M) analysis, *p* values for Kaplan-Meier curves were determined using a log-rank test. **(G)** Western Blot and qRT-PCR assay were performed to prove the knockdown of NONO. **(H)** EdU assays determined the proliferation of cells under NONO knockdown. **(I)** Western blot assays were used to detect the expression of cell cycle-related protein levels in NONO knockdown. **(J)** Western blot analysis was performed to detect the expression of metastasis-related protein levels in NONO knockdown cells. Data shown as means ± SD. The *p* values were calculated using an unpaired two-tailed Student’s t test; ∗*p* < 0.05, ∗∗*p* < 0.01, ∗∗∗*p* < 0.001

In order to investigate the role of NONO with poorly known function in GC, we analyzed mixed stomach adenocarcinoma (2022-v32) data retrieved from The Cancer Genome Atlas Program (TCGA). Higher level of NONO mRNAs was observed in GC tissues than that of normal gastric tissues (Fig. 1A and Fig. S1A). The expression of NONO was examined with RT-PCR and Western blot in cell lines including normal cell lines (GES-1), GC cell lines (BGC-823, HGC-27, MKN-45, SGC-7901), and human-derived gastric cancer cells (GC-1), and the results consolidated the upregulation of NONO in GC (Fig. 1B). Furthermore, the expression of NONO mRNAs was higher in recurrent tissue than that in primary tissues, higher in *TP53* mutant tissues than that in TP53 wild-type (WT) tissues (Fig. 1C-D). Immunohistochemistry (IHC) staining of GC tissue microarray (8 cases of normal gastric tissues and 40 cases of GC tissues) showed a trend of increasing expression of NONO with the tumor stage (Fig. 1E). Kaplan–Meier survival analysis showed that GC patients with high NONO expression had a lower overall survival (OS) than those with low NONO expression (Fig. 1F). In contrast, no significant correlation was observed between NONO expression and gender, age (*P* > 0.05) (Supplementary Tables S1 and S2). Additionally, we analyzed the multi-cancer cohort data obtained from the TCGA database and found NONO was overexpressed in multiple other cancers (Fig. S1B). These results suggested NONO may act as an oncogene in gastric cancer progression.

To investigate the potential oncogenic role of NONO in GC cells, the expression of NONO was interfered (Fig. 1G) or upregulated (Fig. S3A, B) in MKN-45 and GC-1 cells, and the cell proliferation, migration, and invasion ability was evaluated. MTT (3-[4,5-dimethylthiazol-2-yl]-2,5 diphenyl tetrazolium bromide) and EdU (5-ethynyl-2′-deoxyuridine) assays showed that downregulation of NONO led to a decreased cell proliferation rate in both MKN-45 and GC-1 cells compared with that of shGFP control (Fig. 1H, Fig. S2A, B). Flow cytometry revealed an increase in the percentage of shNONO cells in the G0/G1 phase (Fig. S2C). The cell cycle checkpoint molecules also demonstrated that knockdown of NONO caused loss of G0/G1 phase-related proteins (Fig. 1I). Fewer colonies formed in the NONO-knockdown group after two weeks (Fig. S2D). Further, the number of migrating and invading cells significantly decreased in GC cells transfected with shNONO compared with shGFP (Fig. S2E). The levels of proteins involved in the epithelial-mesenchymal transition (EMT) and migration were reduced in NONO-knockdown MKN-45 and GC-1 cells (Fig. 1J). In contrast, upregulating the expression of NONO in MKN-45 and GC-1 cells (Fig. S3A, B), the cell proliferation, migration, and invasion was enhanced compared to that of the control (Fig. S3C-E).

**Fig. 2:**
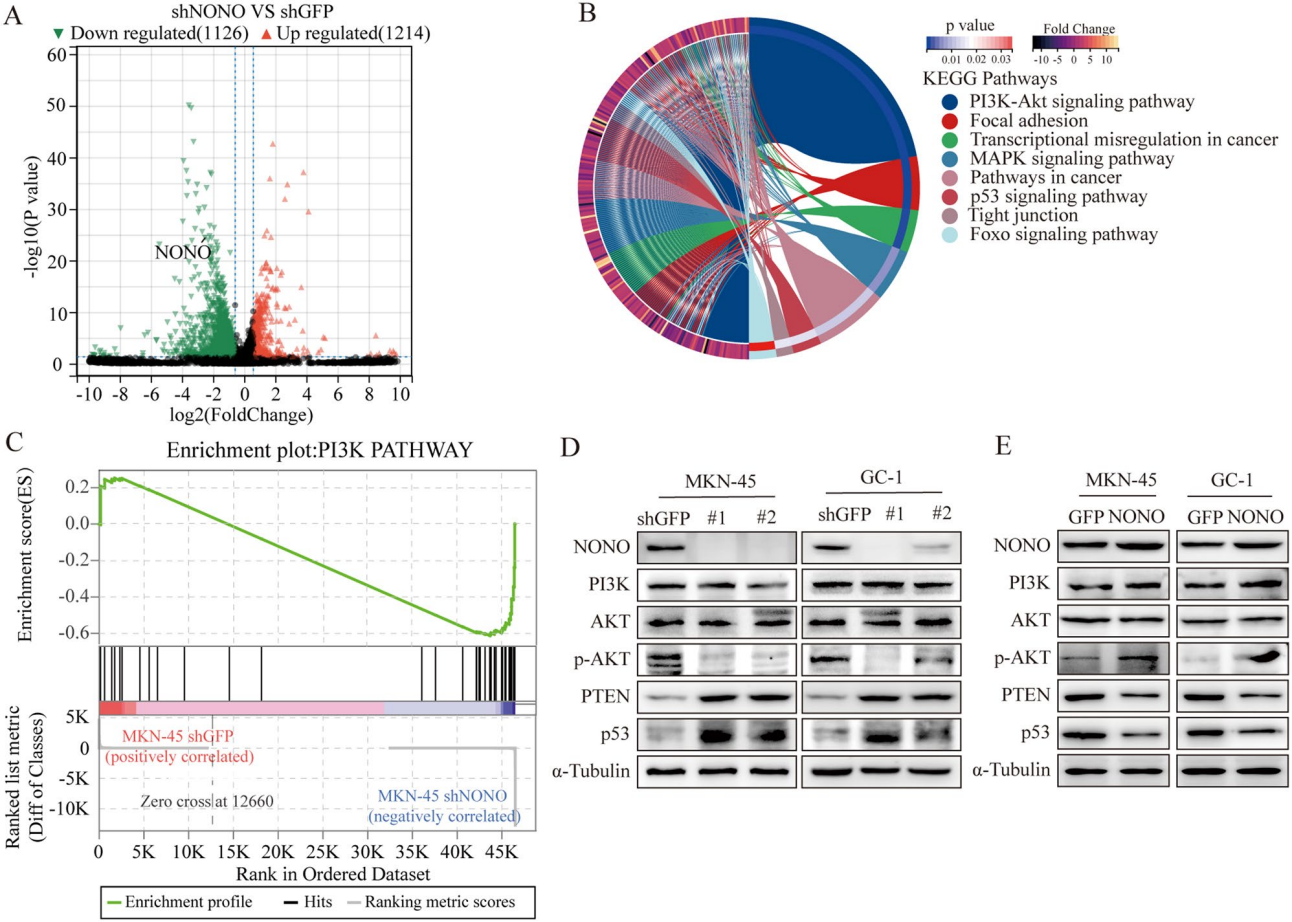
NONO regulated PI3K/AKT signaling pathway by PTEN in GC Cells. **(A)** Volcano plot showing the fold-change (log_2_) in expression levels of mRNA based on shNONO vs. shGFP in MKN-45 cells. **(B)** Chord plot of KEGG pathway enrichment analyses for DEGs. **(C)** GSEA enrichment plots of PI3K signaling pathway in shGFP versus shNONO MKN-45 cells. Normalized enrichment score (NES), false discovery rate (FDR), and *p* values were shown in the plot. **D E.** Western blot analysis of the expression of PI3K-AKT signaling proteins after NONO knockdown and overexpression in MKN-45 and GC-1 cells

To explore the downstream genes affected by NONO in cancer, we systematically analyzed the change of gene expression in patients with high or low expression of NONO using the GSE65801 dataset. KEGG (Kyoto Encyclopedia of Genes and Genomes) analysis revealed enrichment of genes in the PI3K-AKT signaling pathway and p53 signaling pathway (Fig. S4A), which are key pathways that promote cell survival and proliferation. To further verify this consequence, RNA-seq assay was conducted on the MKV-45 cell with knockdown of NONO. The differential genes were filtered with the criteria of conditions (p value < 0.05, |log2 fold change| > 1.5); a total of 1214 genes with significant up-regulation and 1126 genes with significant down‐regulation in the shNONO GC cells were identified, compared to that of the control group (Fig. 2A). KEGG analysis revealed that PI3K-AKT signaling pathway indeed was changed significantly (Fig. 2B). GSEA (Gene Set Enrichment Analysis) analysis confirmed that knockdown of NONO made a significant change of PI3K signaling (Fig. 2C). Previous studies have reported that PTEN negatively regulates AKT activation through PI(3,4,5)P3 dephosphorylation [24] and enhances p53 transactivation [25]. A negative correlation was found between the NONO and PTEN expression in GC from the Gastric cohort (Fig. S4B). Western blot results showed NONO knockdown significantly reduced the expression of p-AKT and increased the expression of PTEN and p53 in gastric cancer line MKN-45 cells and GC-1 cells (Fig. 2D), while overexpression of NONO induced opposite changes (Fig. 2E). These results suggest that PTEN and PI3K-AKT signaling pathway might play important role in the NONO-driven cancer.

### The alternative splicing of PTEN mRNAs is directly regulated by NONO

**Fig. 3:**
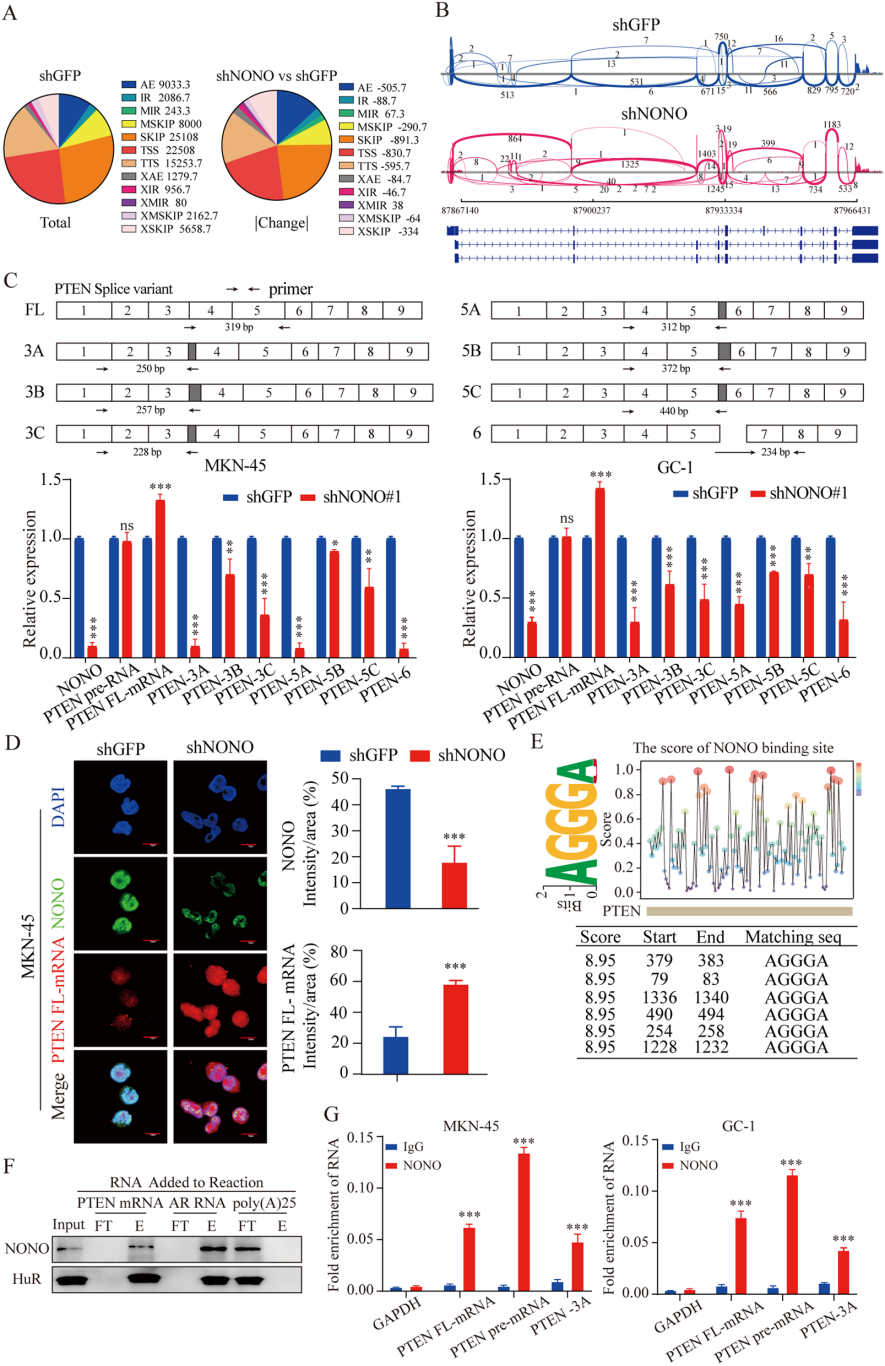
The alternative splicing of PTEN mRNAs is directly regulated by NONO. **(A)** Quantification of AS events after NONO knockdown in MKN-45 cells. Left: the distribution of AS events detected in control (shGFP) cells; Right: the distribution of AS change between shNONO and shGFP cell lines. **(B)** Sashimi plot visualization of RNA-seq reads mapping to PTEN in MKN-45 cells in response to NONO knockdown. **(C)** Real-time qPCR analyses of different PTEN RNAs expressing after NONO knockdown in MKN-45 and GC-1 cells (*n* = 3). **(D)** Representative images of RNA FISH for PTEN mRNA (red) and immunofluorescence for NONO (green) in MKN-45. Scale bar = 10 μm. **(E)** The results of RBPsuite for predicting NONO binding sites on PTEN mRNA. **(F)** RNA-protein pull-down experiment showed the interaction between NONO and PTEN mRNA. Full-length PTEN mRNA was used. Input: lysate load; FT = flow-through, E = eluate; AR (androgen receptor): positive RNA Control, poly(A) 25: negative RNA control. **(G)** The interaction between different PTEN mRNA species and NONO were assayed by RIP-qPCR with lysate of GC cells (*n* = 3). Data shown as means ± SD. The *p* values were calculated using an unpaired two-tailed Student’s t test; ∗*p* < 0.05, ∗∗*p* < 0.01, ∗∗∗*p* < 0.001

Since NONO functions as an essential splicing factor [26], we investigated the potential effect of NONO on AS of *PTEN*. We examined the NONO-regulated AS events in GC and found more than thousands of NONO-related AS events through comparing the different shRNA treatments. The AS events in the transcriptome were classified into 12 types according to their structures. The number of alternative exon end (AE) and single intron (IR) retention of AS events significantly increased in NONO knockdown MKN-45 cells, while the exon skipping (SKIP), alternative transcriptional start site (TSS), and alternative transcription terminal site (TTS) were decreased in NONO knockdown MKN-45 cell (Fig. 3A). Sashimi plot visualization of *PTEN* revealed AS events involving intron 3, intron 5, exon 6 and others (Fig. 3B). To directly assess AS in PTEN genes, we performed RT-PCR with primers specific for pre-mRNA, full-length (FL) mRNA, and various SV mRNAs (Fig. 3C). Although the levels of the pre-mRNAs remained constant, the levels of the FL mRNAs were significantly increased and the other splice forms were significantly decreased in the NONO knockdown GC cell lines (Fig. 3C). The increase in PTEN FL mRNAs upon NONO knockdown was also detected with FISH (Fluorescence in Situ Hybridization) (Fig. 3D, Fig. S4C).

Then we interrogated the interaction between NONO and PTEN pre-mRNAs. Multiple NONO binding motifs were found in the PTEN mRNA sequence (Fig. 3E). RNA-protein pull-down experiments were performed using cell lysate of NONO-overexpressing GC cell line in vitro, and the result showed NONO was specifically pulled down by synthesized full-length (FL) PTEN mRNA and androgen receptor (AR) mRNA (a known target of NONO and a positive control), but not by polyA (25) RNA (a negative control) (Fig. 3F). Furthermore, RIP-qPCR assay with purified recombinant NONO protein showed that the amount of PTEN pre-mRNA and FL-mRNA were increased upon NONO-knockdown in GC cell lines, while that of PTEN SV 3 A (as a representative of SVs) was decreased (Fig. 3G).

To further validate this effect and exclude the possibility that the observed splicing differences of PTEN might reflect RNA degradation in NONO knockdown, an RNA stability assay was performed by the transcription inhibitor actinomycin D. A significantly shortened PTEN FL-mRNA half-life after NONO knockdown, which conflicted with the phenomenon that PTEN FL-mRNA is elevated in NONO deficiency (Fig. S4D). Collectively, these results indicated that NONO could directly bind to PTEN pre-mRNA to mediate the AS events.

### The m5C pattern at PTEN pre-mRNA is affected by NONO-mediated NSUN2 recruitment

**Fig. 4:**
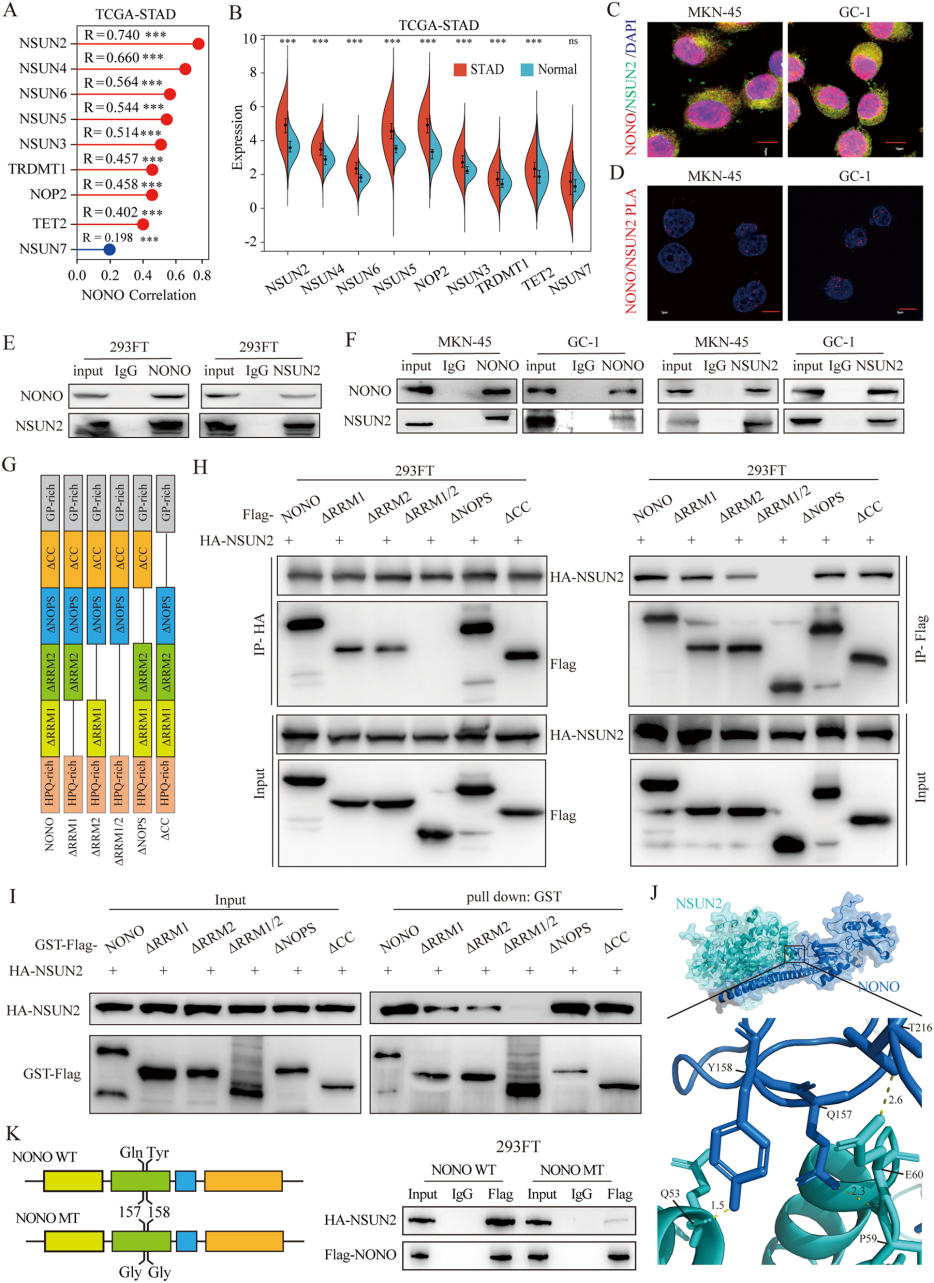
NONO Interacts with NSUN2 in GC. **(A)** Correlation between NONO expression and RNA m^5^C relative genes expression in TCGA-ATAD. R: Pearson’s correlation coefficient. *p* value was based on two-sided Pearson’s correlation test. **(B)** The expression level of RNA m^5^C relative genes in TCGA-STAD. **(C)** NONO and NSUN2 colocalize in the nucleus. Immunofluorescence analysis was performed using anti-NONO and -NSUN2 antibodies in MKN-45 and GC-1 cells. Scale bar = 10 μm. **(D)** NONO interacts with NSUN2. Duolink PLA was performed with indicated antibodies. Scale bar = 10 μm. **E F.** NONO andNSUN2 interact in 293FT cells, MKN-45 and GC-1 cells. **G H.** Detection of the functional domain of NONO required for its interaction with NSUN2. Schematic diagram of truncated construct of NONO(G). HEK-293FT cells were cotransfected with Flag-NONO truncations and HA-NSUN2 for 48 h before Co-IP assay (H). **I.** GST pull-down assay was utilized to detection of the functional domain of NONO required for its interaction with NSUN2. GST-tagged Flag-NONO and NONO truncations were incubated with purified HA-NSUN2. Proteins were separated on SDS–PAGE and subjected to Western blotting analysis. **J.** The overall structure of the human NONO-NSUN2 complex. Hydrogen bonds are indicated with yellow dashes. **K.** Q157 and Y158 mutation of NONO abolishes the interaction between NONO and NSUN2. NONO mutants are illustrated schematically at the left. WT or MT NONO protein was immunoprecipitated with Flag beads and then immunoblotted with anti-HA or anti-Flag antibodies

To explore the mechanism of NONO-mediated AS regulation of *PTEN*, we used the normalized pan-cancer dataset (TCGA, PANCAN, *N* = 10535, G = 60499) from the UCSC database (https://xenabrowser.net/) and found broad correlation (Pearson) between the transcription of *NONO* and 41 RNA modification genes (10 m^1^A-related genes, 10 m^5^C-related genes, 21 m^6^A-related genes) in 37 cancer types (Fig. S5A). The expression of NONO and some m^5^C-related genes was positively correlate in gastric cancer, and the strongest correlation was NSUN2, and the expression of these genes were increased compared with normal gastric tissue (Fig. 4A-B, Fig. S5B). Mass spectrometry assay identified NSUN2 as a NONO-interacting protein among the RNA modification related proteins (Fig. S5C and Table S11). Immunofluorescence assay showed colocalization of NONO and NSUN2 in the nucleus of GC cells (Fig. 4C). Furthermore, Duolink PLA (Fig. 4D) and Co-immunoprecipitation (Co-IP) (Fig. 4E-F) experiments indicated the interaction between them.

A Co-IP experiments with various truncated mutants of NONO and NSUN2 expressed in HEK-293FT cells showed that the positive signal was diminished upon single deletion of RRM1 or RRM2 domain of NONO. Intriguingly, the interacting signal was undetectable upon double deletion of RRM1/2 domains (Fig. 4G and H), indicating RRM1/2 domains of NONO were required for its interaction with NSUN2. In vitro GST pull-down assays with purified proteins further consolidated the declaration (Fig. 4I). To identify which amino acid of NONO binds NSUN2, we modeled the structure of these protein complexes using the Zdock. Interestingly, in complexes of NONO with NSUN2, the Q157 and Y158 of NONO is interaction with NSUN2(Fig. 4J). To test these predictions, we assessed whether the mutant NONO (Q157G and Y158G) mediate binding to full-length NSUN2 protein in GC cell lines. As expected, compared with the wild-type(WT) NONO, the binding of NONO mutant (MT) with NSUN2 was significantly decreased (Fig. 4K), and the PTEN expression was nonsignificantly rescued (Fig. S5D).

**Fig. 5:**
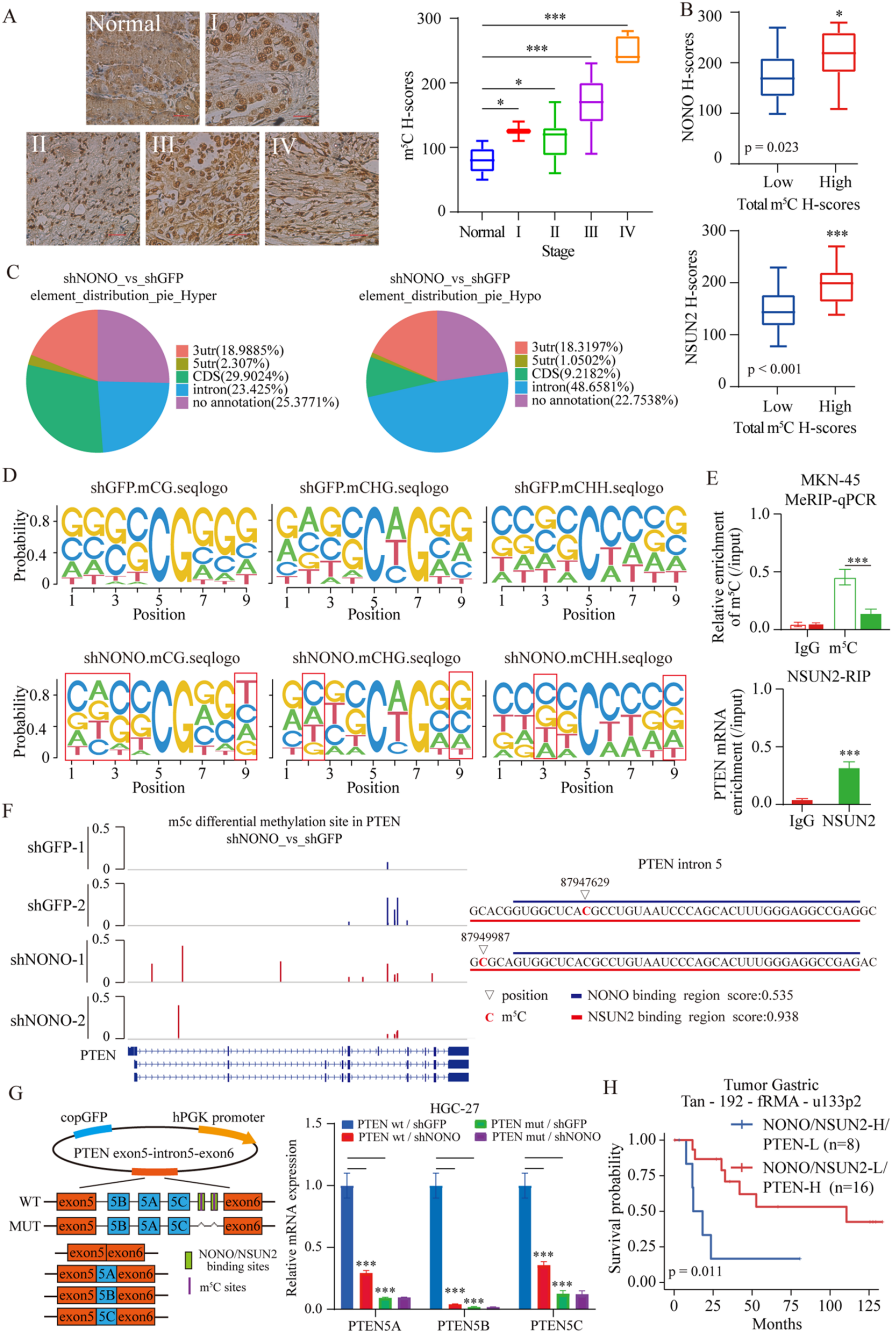
The m^5^C pattern at PTEN mRNAs is affected by NONO and NSUN2. **(A)** Representative images of immunohistochemical staining for m^5^C methylation in a tissue microarray (scale bar = 100 μm; left) and the differential levels of m^5^C methylation between different stage tumors and normal tissue. The horizontal bars in the boxplots represent the median and the box edges represent the first and third interquartile ranges. The *p* values were calculated using a two-sided unpaired Student’s t-test. **(B)** Distribution of the protein expression of NONO or NSUN2 in high expression of m^5^C methylation tumors compared with matched low expression samples. The horizontal bars in the boxplots represent the median and the box edges represent the first and third interquartile ranges. The *p* values were calculated using a two-sided unpaired Student’s t-test. **(C)** Distribution of differential m^5^C sites in GC. The differential m^5^C sites were defined as follows: mean m^5^C level difference ≥ 0.05 (shNONO and shGFP) and *p* < 0.05 (Wilcoxon test). **(D)** m^5^C sequence frequency logo in GC transcripts. **(E)** MeRIP-qPCR indicated the PTEN mRNA enrichment precipitated by m^5^C antibody (up) and RIP-qPCR indicated the PTEN mRNA enrichment precipitated by NSUN2 antibody (down) in MKN-45 cell line. **(F)** Integrative-genomics-viewer tracks displaying the read coverage and m^5^C levels of PTEN in shGFP and shNONO MKN-45 cells (left). and RBPsuite was used to predict the binding sites of NONO and NSUN2 along PTEN intron 5 (right). **(G)** Minigene assay for verifying coordination between NONO and NSUN2. Graphical representation of the control splicing reporter (WT) and the mutation splicing reporter (MUT: specifically introducing mutations to NONO/NSUN2-binding sites; green) (left). qRT-PCR analyses were performed for the PTEN minigenes after NONO knockdown (right). **(H)** Kaplan–Meier analysis indicated the correlation between the combination of high expression of NONO, NSUN2, and low expression PTEN with a poorer OS. *p* values for Kaplan-Meier curves were determined using a log-rank test. Data shown as means ± SD. The *p* values were calculated using an unpaired two-tailed Student’s t test; ∗*p* < 0.05, ∗∗*p* < 0.01, ∗∗∗*p* < 0.001

IHC analysis of 40 GC tumors illustrated an increased global RNA m^5^C content in tumor samples (Fig. 5A) and the m^5^C methylation levels of tissue correlated positively with NONO and NSUN2 expression (Fig. 5B and Fig. S6A). This result was confirmed by m^5^C dot blot analysis using the total RNA isolated from NONO or NSUN2 knockdown cells (Fig. S6B). We map the transcriptome-wide m^5^C modifications at a single-base resolution with an improved RNA bisulfite sequencing (RNA-BisSeq) method in the shGFP and shNONO MKN-45 cells. There were 1984 m^5^C sites with different methylation level following NONO knockdown, which was distributed on different chromosomes (Fig. S6C). Among these, 1503 differential m^5^C sites occurred in mRNAs. The differential m^5^C sites showed enrichment in the intron, CDS and 3′ UTRs (Fig. 5C). Differential analysis of transcriptome data and methylation sequencing showed that 95 genes were changed in expression level and m^5^C modification after NONO knockdown (Table S13). A sequence frequency analysis at CG, CHG, and CHH (where H = A, C, U) contexts displayed that NONO knockdown affected the sequence preference near the methylation sites, with CG-contained sequences most affected (Fig. 5D). MeRIP-qPCR showed that the m^5^C modification of PTEN mRNA was significantly reduced after NSUN2 knockdown, and RIP-qPCR suggested that NSUN2 could bind to PTEN mRNA (Fig. 5E). Furthermore, the m^5^C levels of total RNA were significantly rescued after NSUN2 overexpression in NONO knockdown cells (Fig. S6D).

Then, RBPsuite [27], a deep-learning based model, was used to predict the potential binding between the NONO/NSUN2 and PTEN pre-mRNA, and shown that NONO/NSUN2 could binding to PTEN intron 5, there are including the two sites with m^5^C-modification (Fig. 5F). Next, we analyzed the local structure of this m^5^C sites. We found that the clustered sites tended to be located in the stem region (Fig. S6E). To further confirm the regulation of *PTEN* splicing by NONO and NSUN2, we constructed a splicing minigene reporter containing the genomic region from PTEN exons 5 to 6. As expected, knockdown of NONO promoted the inclusion of PTEN intron 5A/B/C splicing compared to the shGFP. The splicing of PTEN intron 5A/B/C was markedly impaired when the NONO/NSUN2-binding sites were mutated (Fig. 5G). These results suggested that NONO acts in conjunction with NSUN2 to regulate AS and that loss of either protein results in splicing dysregulation in terms of PTEN intron 5 splicing in HGC-27 cells. The expression of PTEN was negatively correlated with the expression of NSUN2 in gastric tumor (Fig. S6F). IHC analysis showed that the PTEN levels of tissue correlated negatively with m^5^C methylation and NSUN2 expression (Fig. S6G-H). Survival analysis illustrated that high expression of NONO /NSUN2 and low expression of PTEN predicted poor OS (Fig. 5H). The expression of NONO and NSUN2 was higher in the gastric tissue, which was in contrast to the expression of PTEN (Fig. S6I), suggesting that the NONO-NSUN2-PTEN regulatory axis is a bona fide signaling pathway critical to tumor pathogenesis and patient prognosis in human GC.

### NONO/NSUN2/PTEN axis promotes GC progression

**Fig. 6:**
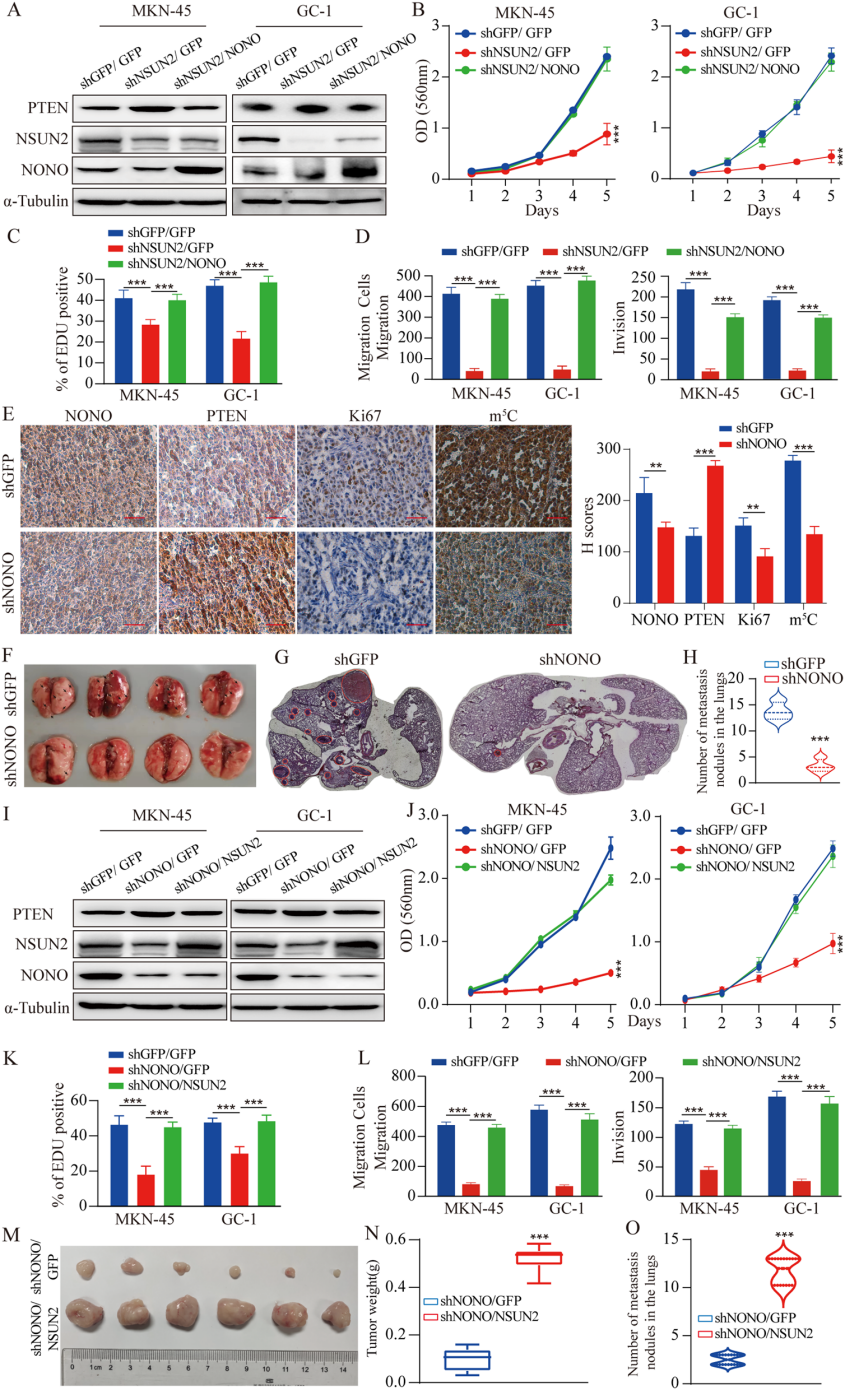
NONO promotes GC pathogenesis in an m^5^C-dependent mechanism. **A.** PTEN expression was analyzed by Western blot under restore NONO after Knockdown NSUN2 as indicated. **B C D.** The proliferation and invasion of cells under overexpression NONO after NSUN2 knockdown was determined via MTT (B), EdU (C) and Transwell (D) assays. **E.** IHC for NONO, PTEN, Ki67 and m^5^C levels in the GC xenografts. Scale bar = 100 μm. **F G H.** Representative images of the lung and H&E staining. H&E staining of lung sections from nude mice that were tail-vein injected with NONO knockdown MKN-45 cells. Numbers of lung nodule per animal are shown in the right as mean ± SD (*n* = 4). **I.** PTEN expression was analyzed by Western blot under restore NSUN2 after Knockdown NONO as indicated. **J K L.** The proliferation and invasion of cells under overexpression NSUN2 after NONO knockdown was determined via MTT, EdU and transwell assays (*n* = 3). **M N.** Xenograft assays were performed in MKN-45 cells under overexpression NSUN2 after NONO knockdown (*n* = 6). The weight of tumors was analyzed and P-values were indicated. **O.** Numbers of lung nodule per animal are shown as mean ± SD (*n* = 4). Data shown as means ± SD. The *p* values were calculated using an unpaired two-tailed Student’s t test. ∗*p* < 0.05, ∗∗*p* < 0.01, ∗∗∗*p* < 0.001

Similar to NONO depletion, NSUN2 knockdown significantly changed the PTEN expression (Fig. 6A) and pre-mRNA splicing events (Fig. S7A), which were rescued by overexpression of NONO. MTT (Fig. 6B), EDU (Fig. 6C, Fig. S7B) experiments showed that the inhibitory effect of NSUN2-knockdown on the GC cell proliferation was reversed by the overexpression of NONO. Similar result was observed in term of the migration ability of GC cells in the Transwell experiment (Fig. 6D; Fig. S7C). Supporting this, the results from GC cell line-based xenograft (CDX) models showed that NONO or NSUN2 knockdown significantly inhibited the subcutaneous growth of CDX tumors generated from the MKN-45 cell line (Fig. S7D). Subsequently, IHC analyses show that depletion of NONO significantly inhibits cell growth and the level of m^5^C compared with the control (Fig. 6E). We also assessed the influence of NONO on lung invasion. The amount of pulmonary metastatic nodules was obviously reduced in the shNONO group compared with that of shGFP control group (Fig. 6F). Hematoxylin-eosin (HE) staining of lung tissues and statistics showed the median number of metastatic nodules was 3 in the control group, while only 14 in the shNONO group (Fig. 6G and H).

Interestingly, the overexpression of NSUN2 efficiently rescued the PTEN expression (Fig. 6I) and pre-mRNA splicing events (Fig. S8A) caused by NONO-knockdown. MTT (Fig. 6J) and EDU (Fig. 6K, Fig. S8B) experiments showed that the inhibitory effect of NONO-knockdown on the GC cell proliferation was reversed by the overexpression of NSUN2. Similar result was observed in term of the migration ability of GC cells in the Transwell experiment (Fig. 6L; Fig. S8C). Moreover, NSUN2 overexpression compromised the shNONO-induced tumor burden (Fig. 6M-N). The effects of the overexpression of NSUN2 on NONO knockdown-induced pulmonary invasion were also determined in vivo. The NSUN2 overexpression significantly increased the capacity of MKN-45 cells to metastasize from the tail vein to the lungs, as determined by the number and the volume of nodules (Fig. 6O), suggesting that the overexpression of NSUN2 can increase the lung invasion, which was inhibited by NONO knockdown. Encouragingly, the PTEN overexpression inhibited the impact of NONO and NSUN2 on the growth of GC cells (Fig. S8D). Taken together, we provide evidence that NONO/NSUN2/PTEN axis regulates the proliferation and invasion abilities of GC cells.

## Discussion

Gastric cancer (GC) remains the fifth most common malignant cancer and the fourth leading cause of cancer-related mortality globally [28]. Despite significant clinical and surgical improvements, the prognosis of metastatic, recurrent, and advanced GC is still unsatisfactory, and the 5-year survival rate of GC is low, as more than 80% of patients are diagnosed at an advanced stage [17]. Hence, there is an urgent need to investigate the mechanisms of GC tumorigenesis and progression to develop new therapeutic strategies. This study unveils a novel regulatory mechanism of tumor suppressor gene inactivation mediated by m^5^C methylation and alternative splicing in GC, which provides profound insights into therapeutic strategy for GC.

**Fig. 7:**
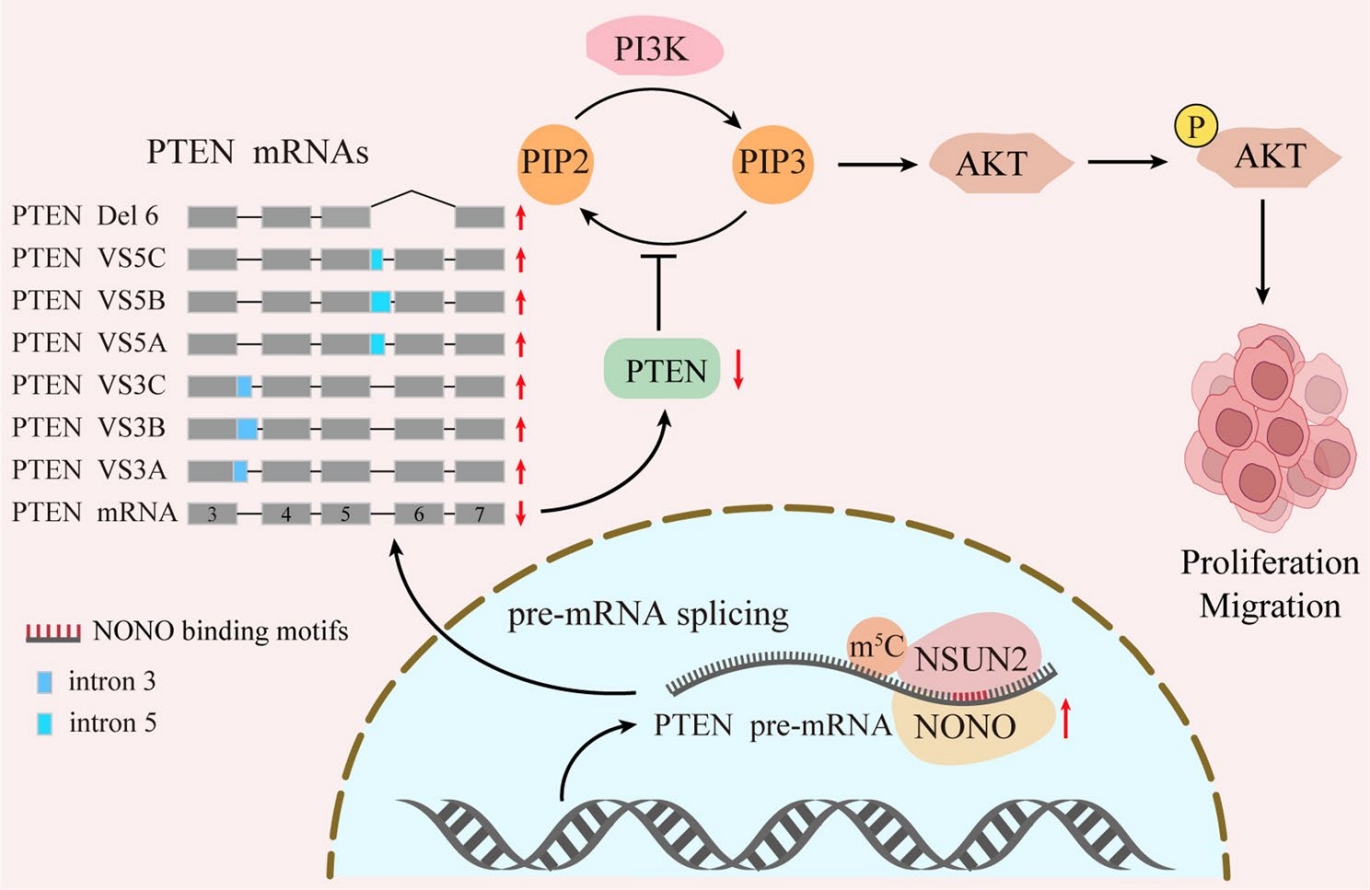
Proposed model for the functional landscape of the NONO–NSUN2–PTEN in gastric cancer

NONO has been identified as an important DBHS family splicing factor [29] and engages in almost every procedure of gene regulation [30], including transcription [31–33], RNA processing [34–39], and transport [40–42]. Previous studies showed the involvement of NONO in the progression of multiple cancers, such as prostate cancer [43, 44], bladder cancer [36], melanoma [45, 46] and GBM [34]. A piece of evidence supported an oncogenic role of NONO in gastric cancer progression [47], however, the underlying mechanism remains to be clarified. Our study revealed NONO-regulated m^5^C modification and alternative splicing of PTEN mRNAs drive gastric cancer progression. As an RNA binding protein, NONO directly recognized PTEN mRNAs, meanwhile recruited NSUN2, which caused AS of PTEN mRNAs in an m^5^C-dependent manner. Consequently, the full-length (FL) PTEN mRNA and SV mRNAs were decreased and increased, respectively (Fig. 7), resulting in reduced protein level of WT PTEN and diminished PTEN signaling pathway. In addition, some SVs with loss-of-function [13] could further compromise the PTEN signaling pathway. There are more than eight SVs of PTEN, further studies are needed to clarify the function of these SV transcripts. It has been reported that the abnormality of some PTEN SV transcripts could not be connected to tumorigenesis [48–51], which might be related to the fact that the proteins produced from SV mRNAs are unstable and degraded rapidly [13, 52, 53]. From the point of view of our study, the flow shift from PTEN WT to SV transcripts per se mattered.

The tumor-suppressor activity of PTEN relies largely on its lipid phosphatase activity that antagonizes PI3K/AKT activation [24]. Cheng et al. found that NONO plays a potent role in multiple biological aspects of ESCC through activation of the AKT signaling pathways [54]. Lone BA et al. reported that NONO is a regulator of AKT/MAPK/β-catenin signaling pathways [55]. The results of our studies further consolidated the regulation of the AKT signaling pathway by NONO. Moreover, we found downregulation of functional PTEN by NONO-mediated m^5^C alteration accounted for the upregulation of the AKT signaling pathway in GC, therefore, filling the gap between NONO expression and the AKT pathway. However, how the m^5^C changes triggered PTEN AS remains an interesting open question that deserves future research.

Recent studies have shown that there may be only a few hundred mRNAs in human transcripts with m^5^C modification [3, 23, 56]. We showed that NONO regulates RNA cytosine methylation of many genes including *PTEN*, by recruiting RNA methyltransferase NSUN2 in gastric cancer. This may explain why the changes of mRNA m^5^C levels after NONO-knockdown were not as big as those after NSUN2-knockout [23]. Convincingly, the sequence feature of sites with methylation changes after NONO-knockdown is very similar to the sequence feature of NSUN2 targets, i.e., containing CG-rich motif and stem-ring structure. Interestingly, the depletion of NONO or NSUN2 may affect the protein level of each other (Fig. 6A and I). Since their transcription were not significantly influenced in the knockdown conditions of each other (Fig. S7A and S8A), we speculated that the interaction between NONO and NSUN2 might stabilize each other. Other possibility also exists, such as alteration in mRNA translation as NONO and NSUN2 have been reported to be involved in the regulation of mRNA translation [40, 57]. Regardless, the mutual promotion between NONO and NSUN2 may aggravate the cancer progression since both serve as oncogene.

## Conclusion

Collectively, we propose a working model in which the NONO/NSUN2/m^5^C-PTEN signaling axis promotes the pathogenesis of GC (Fig. 7). Our findings suggest effective prognostic biomarkers and the opportunity for epitranscriptomic-targeted therapy for GC.

## Electronic supplementary material

Below is the link to the electronic supplementary material.

Supplementary Material 1

Supplementary Material 2

Supplementary Material 3

Supplementary Material 4

Supplementary Material 5

Supplementary Material 6

## Data Availability

RNA sequencing raw data for MKN-45 cells have been deposited in Sequence Read Archive (SRA) database (PRJNA1067164) and are publicly available as of the date of publication. This paper does not report the original code.

## Abbreviations

TSGs: Tumor suppressor genes
AS: Alternative splicing
mRNAs: Messenger RNAs
NSUN2: NOP2/Sun RNA methyltransferase family member 2
UTRs: Untranslated regions
SVs: Splicing variants
GC: Gastric cancer
RRM: RNA-recognition motif
IHC: Immunohistochemistry
Co-IP: Co-immunoprecipitation
MTT: Methylthiazolyldiphenyl-tetrazolium bromide
EdU: 5-Ethynyl-2′-deoxyuridine
RIP: RNA immunoprecipitation
EMT: Epithelial-mesenchymal transition
KEGG: Kyoto Encyclopedia of Genes and Genomes
GSEA: Gene Set Enrichment Analysis
SKIP: Exon skipping
TSS: Alternative transcriptional start site
TTS: Alternative transcription terminal site
FISH: Fluorescence in Situ Hybridization
AR: Androgen receptor
CDX: Cell line-based xenograft
