# Supplementary material for: NONO regulates m5C modification and alternative splicing of PTEN mRNAs to drive gastric cancer progression
Source: J Exp Clin Cancer Res. 2025 Mar 4;44:81. doi: 10.1186/s13046-024-03260-z (PMC11877715; doi:10.1186/s13046-024-03260-z)
Supplement: Supplementary file 5 — Supplementary Material 5 [file 13046_2024_3260_MOESM5_ESM.htm]

  


 
PDBe < PISA < EMBL-EBI

 

- Skip to main content
- Skip to local navigation
- Skip to EBI global navigation menu
- Skip to expanded EBI global navigation menu (includes all sub-sections)

- Services
- Research
- Training
- Industry
- About us

# Protein Data Bank in Europe

# Bringing Structure to Biology

# PDBePISA

pdbe.org/pisa

- Feedback
- Share

PISA Interface.

  


|  |  |  |  |  |  |  |  |  |  |  |  |  |  |  |  |  |
| --- | --- | --- | --- | --- | --- | --- | --- | --- | --- | --- | --- | --- | --- | --- | --- | --- |
| |  |  |  |  |  | | --- | --- | --- | --- | --- | | Session Map  (id=634-88-1J3) | | | | | | Start | Interfaces | Interface Search | |  | Monomers |  | |  | Assemblies |  | | |  | | --- | | **interface # 1 in ZGC.pdb** | |

---

|  |  |  |
| --- | --- | --- |
| **interface #1/1**      XML   <<   <   >   >> | | |

|  |  |  |
| --- | --- | --- |
| **Interface Summary**      XML |  |  |
| |  | | | | **Structure 1** | | **Structure 2** | | | --- | --- | --- | --- | --- | --- | --- | --- | | **Selection range** | | | | B | | A | | | class | | | | Protein | | Protein | | | symmetry operation | | | | x,y,z | | ,, | | | symmetry ID | | | | 1\_555 | | 0\_555 | | | **Number of atoms** | | | | | interface | | | | 129 | 4.9% | 128 | 2.5% | | surface | | | | 1905 | 72.4% | 2845 | 55.9% | | total | | | | 2630 | 100.0% | 5087 | 100.0% | | **Number of residues** | | | | | interface | | | | 31 | 9.8% | 35 | 5.5% | | surface | | | | 302 | 95.9% | 577 | 91.2% | | total | | | | 315 | 100.0% | 633 | 100.0% | | **Solvent-accessible area, Å** | | | | | interface | | | | 1248.9 | 4.9% | 1113.7 | 3.5% | | total | | | | 25306.1 | 100.0% | 31803.6 | 100.0% | | **Solvation energy, kcal/mol** | | | | | isolated structure | | | | -205.1 | 100.0% | -645.0 | 100.0% | | gain on complex formation | | | | -5.0 | 2.5% | -2.2 | 0.3% | | average gain | | | | -3.2 | 1.5% | -1.8 | 0.3% | | P-value | | | | 0.346 |  | 0.470 |  | |  | View Download |
|  |  | This interface scored   **0.000**  in Complex Formation Significance Score (CSS).  CSS ranges from 0 to 1 as interface relevance to complex formation increases. Achieved CSS implies that the interface does not play any role in complex formation and seems to be a result of crystal packing only |

|  |
| --- |
|  |
| |  | | --- | | **Hydrogen bonds**      XML | | | ## | **Structure 1** | **Dist. [Å]** | **Structure 2** | | --- | --- | --- | --- | | `1` | `B:ARG 184[ NH1]` | `3.77` | `A:LYS  43[ O  ]` | | `2` | `B:GLN 157[ NE2]` | `3.80` | `A:VAL  58[ O  ]` | | `3` | `B:GLN 157[ NE2]` | `2.26` | `A:PRO  59[ O  ]` | | `4` | `B:THR 216[ OG1]` | `2.64` | `A:GLU  60[ OE2]` | | `5` | `B:HIS 305[ NE2]` | `2.82` | `A:GLU 407[ OE2]` | | `6` | `B:LYS 290[ NZ ]` | `3.47` | `A:HIS 415[ O  ]` | | `7` | `B:TYR 158[ OH ]` | `3.68` | `A:GLU  54[ N  ]` | | `8` | `B:GLU 291[ O  ]` | `3.64` | `A:LYS 356[ NZ ]` | | `9` | `B:MET 298[ SD ]` | `3.41` | `A:HIS 416[ ND1]` | | `10` | `B:MET 298[ SD ]` | `3.41` | `A:TRP 357[ N  ]` | | `11` | `B:VAL 309[ O  ]` | `3.01` | `A:GLN 402[ NE2]` | | | |  | | --- | | **Salt bridges**      XML | | | ## | **Structure 1** | **Dist. [Å]** | **Structure 2** | | --- | --- | --- | --- | | `1` | `B:ARG 184[ NE ]` | `3.80` | `A:GLU  44[ OE1]` | | `2` | `B:ARG 186[ NH2]` | `3.93` | `A:ASP  68[ OD1]` | | `3` | `B:HIS 305[ NE2]` | `2.82` | `A:GLU 407[ OE2]` | | `4` | `B:GLU 294[ OE1]` | `3.56` | `A:HIS 415[ NE2]` | | | No disulfide bonds found |

|  |  |  |
| --- | --- | --- |
|  | | |
| **Interfacing residues** (not a contact table)      XML  Display level:  Residues Interface atoms Surface atoms | | |
| |  |  |  |  |  |  |  |  |  |  |  | | --- | --- | --- | --- | --- | --- | --- | --- | --- | --- | --- | |  | Inaccessible residues | | | |  | HSDC | Residues making **H**ydrogen/**D**isulphide bond, **S**alt bridge or **C**ovalent link | | | | |  | Solvent-accessible residues | | | |  |  | Interfacing residues | | | | | **ASA** | Accessible Surface Area, Å² |  | **BSA** | Buried Surface Area, Å² |  | **ΔiG** | Solvation energy effect, kcal/mol |  | |||| | Buried area percentage, one bar per 10% | |  | | | | | | | | | | | | | |
| | ## | **Structure 1** | **HSDC** | **ASA** | **BSA** | **ΔiG** | | --- | --- | --- | --- | --- | --- | | `1` | `B:THR  56` |  | `194.29` | `0.00` | `0.00` | | `2` | `B:ILE  57` |  | `142.63` | `0.00` | `0.00` | | `3` | `B:ASP  58` |  | `97.11` | `0.00` | `0.00` | | `4` | `B:LEU  59` |  | `77.60` | `0.00` | `0.00` | | `5` | `B:LYS  60` |  | `167.21` | `0.00` | `0.00` | | `6` | `B:ASN  61` |  | `131.92` | `0.00` | `0.00` | | `7` | `B:PHE  62` |  | `44.53` | `0.00` | `0.00` | | `8` | `B:ARG  63` |  | `132.32` | `0.00` | `0.00` | | `9` | `B:LYS  64` |  | `114.74` | `0.00` | `0.00` | | `10` | `B:PRO  65` |  | `140.09` | `0.00` | `0.00` | | `11` | `B:GLY  66` |  | `77.21` | `0.00` | `0.00` | | `12` | `B:GLU  67` |  | `29.54` | `0.00` | `0.00` | | `13` | `B:LYS  68` |  | `70.54` | `0.00` | `0.00` | | `14` | `B:THR  69` |  | `51.03` | `0.00` | `0.00` | | `15` | `B:PHE  70` |  | `68.07` | `0.00` | `0.00` | | `16` | `B:THR  71` |  | `9.32` | `0.00` | `0.00` | | `17` | `B:GLN  72` |  | `15.12` | `0.00` | `0.00` | | `18` | `B:ARG  73` |  | `80.25` | `0.00` | `0.00` | | `19` | `B:SER  74` |  | `0.23` | `0.00` | `0.00` | | `20` | `B:ARG  75` |  | `42.98` | `0.00` | `0.00` | | `21` | `B:LEU  76` |  | `0.00` | `0.00` | `0.00` | | `22` | `B:PHE  77` |  | `54.82` | `0.00` | `0.00` | | `23` | `B:VAL  78` |  | `0.33` | `0.00` | `0.00` | | `24` | `B:GLY  79` |  | `4.19` | `0.00` | `0.00` | | `25` | `B:ASN  80` |  | `73.85` | `0.00` | `0.00` | | `26` | `B:LEU  81` |  | `0.96` | `0.00` | `0.00` | | `27` | `B:PRO  82` |  | `3.52` | `0.00` | `0.00` | | `28` | `B:PRO  83` |  | `90.77` | `0.00` | `0.00` | | `29` | `B:ASP  84` |  | `78.25` | `0.00` | `0.00` | | `30` | `B:ILE  85` |  | `1.84` | `0.00` | `0.00` | | `31` | `B:THR  86` |  | `56.71` | `0.00` | `0.00` | | `32` | `B:GLU  87` |  | `80.90` | `0.00` | `0.00` | | `33` | `B:GLU  88` |  | `138.78` | `0.00` | `0.00` | | `34` | `B:GLU  89` |  | `54.14` | `0.00` | `0.00` | | `35` | `B:MET  90` |  | `0.17` | `0.00` | `0.00` | | `36` | `B:ARG  91` |  | `87.31` | `0.00` | `0.00` | | `37` | `B:LYS  92` |  | `152.50` | `0.00` | `0.00` | | `38` | `B:LEU  93` |  | `32.16` | `0.00` | `0.00` | | `39` | `B:PHE  94` |  | `0.08` | `0.00` | `0.00` | | `40` | `B:GLU  95` |  | `118.54` | `0.00` | `0.00` | | `41` | `B:LYS  96` |  | `122.51` | `0.00` | `0.00` | | `42` | `B:TYR  97` |  | `5.59` | `0.00` | `0.00` | | `43` | `B:GLY  98` |  | `35.29` | `0.00` | `0.00` | | `44` | `B:LYS  99` |  | `146.27` | `0.00` | `0.00` | | `45` | `B:ALA 100` |  | `25.88` | `0.00` | `0.00` | | `46` | `B:GLY 101` |  | `28.10` | `0.00` | `0.00` | | `47` | `B:GLU 102` |  | `69.10` | `0.00` | `0.00` | | `48` | `B:VAL 103` |  | `21.02` | `0.00` | `0.00` | | `49` | `B:PHE 104` |  | `101.05` | `0.00` | `0.00` | | `50` | `B:ILE 105` |  | `32.75` | `0.00` | `0.00` | | `51` | `B:HIS 106` |  | `49.64` | `0.00` | `0.00` | | `52` | `B:LYS 107` |  | `104.75` | `0.00` | `0.00` | | `53` | `B:ASP 108` |  | `132.72` | `0.00` | `0.00` | | `54` | `B:LYS 109` |  | `136.62` | `0.00` | `0.00` | | `55` | `B:GLY 110` |  | `11.98` | `0.00` | `0.00` | | `56` | `B:PHE 111` |  | `45.66` | `0.00` | `0.00` | | `57` | `B:GLY 112` |  | `0.00` | `0.00` | `0.00` | | `58` | `B:PHE 113` |  | `42.18` | `0.00` | `0.00` | | `59` | `B:ILE 114` |  | `0.31` | `0.00` | `0.00` | | `60` | `B:ARG 115` |  | `41.10` | `0.00` | `0.00` | | `61` | `B:LEU 116` |  | `0.00` | `0.00` | `0.00` | | `62` | `B:GLU 117` |  | `54.69` | `0.00` | `0.00` | | `63` | `B:THR 118` |  | `6.36` | `0.00` | `0.00` | | `64` | `B:ARG 119` |  | `49.76` | `0.00` | `0.00` | | `65` | `B:THR 120` |  | `33.06` | `0.00` | `0.00` | | `66` | `B:LEU 121` |  | `14.40` | `0.00` | `0.00` | | `67` | `B:ALA 122` |  | `0.00` | `0.00` | `0.00` | | `68` | `B:GLU 123` |  | `60.49` | `0.00` | `0.00` | | `69` | `B:ILE 124` |  | `55.71` | `0.00` | `0.00` | | `70` | `B:ALA 125` |  | `0.00` | `0.00` | `0.00` | | `71` | `B:LYS 126` |  | `30.17` | `0.00` | `0.00` | | `72` | `B:VAL 127` |  | `115.57` | `0.00` | `0.00` | | `73` | `B:GLU 128` |  | `89.83` | `0.00` | `0.00` | | `74` | `B:LEU 129` |  | `5.35` | `0.00` | `0.00` | | `75` | `B:ASP 130` |  | `25.84` | `0.00` | `0.00` | | `76` | `B:ASN 131` |  | `72.54` | `0.00` | `0.00` | | `77` | `B:MET 132` |  | `59.20` | `0.00` | `0.00` | | `78` | `B:PRO 133` |  | `103.55` | `0.00` | `0.00` | | `79` | `B:LEU 134` |  | `28.79` | `0.00` | `0.00` | | `80` | `B:ARG 135` |  | `135.33` | `0.00` | `0.00` | | `81` | `B:GLY 136` |  | `84.61` | `0.00` | `0.00` | | `82` | `B:LYS 137` |  | `71.25` | `0.00` | `0.00` | | `83` | `B:GLN 138` |  | `113.18` | `0.00` | `0.00` | | `84` | `B:LEU 139` |  | `2.33` | `0.00` | `0.00` | | `85` | `B:ARG 140` |  | `111.93` | `0.00` | `0.00` | | `86` | `B:VAL 141` |  | `0.00` | `0.00` | `0.00` | | `87` | `B:ARG 142` |  | `47.75` | `0.00` | `0.00` | | `88` | `B:PHE 143` |  | `47.65` | `0.00` | `0.00` | | `89` | `B:ALA 144` |  | `12.74` | `0.00` | `0.00` | | `90` | `B:CYS 145` |  | `112.39` | `0.00` | `0.00` | | `91` | `B:HIS 146` |  | `20.94` | `0.00` | `0.00` | | `92` | `B:SER 147` |  | `67.16` | `0.00` | `0.00` | | `93` | `B:ALA 148` |  | `1.85` | `0.00` | `0.00` | | `94` | `B:SER 149` |  | `10.89` | `0.00` | `0.00` | | `95` | `B:LEU 150` |  | `3.76` | `0.00` | `0.00` | | `96` | `B:THR 151` |  | `17.54` | `0.00` | `0.00` | | `97` | `B:VAL 152` |  | `0.00` | `0.00` | `0.00` | | `98` | `B:ARG 153` |  | `110.45` | `0.00` | `0.00` | | `99` | `B:ASN 154` |  | `46.76` | `0.00` | `0.00` | | `100` | `B:LEU 155` |  | `12.77` | `7.85`  ||||||| | `-0.09` | | `101` | `B:PRO 156` |  | `8.20` | `3.67`  ||||| | `0.06` | | `102` | `B:GLN 157` | `H` | `130.27` | `121.52`  |||||||||| | `-0.58` | | `103` | `B:TYR 158` | `H` | `197.76` | `116.26`  |||||| | `0.76` | | `104` | `B:VAL 159` |  | `13.21` | `0.00` | `0.00` | | `105` | `B:SER 160` |  | `48.23` | `0.00` | `0.00` | | `106` | `B:ASN 161` |  | `61.51` | `0.00` | `0.00` | | `107` | `B:GLU 162` |  | `116.17` | `0.00` | `0.00` | | `108` | `B:LEU 163` |  | `61.47` | `0.00` | `0.00` | | `109` | `B:LEU 164` |  | `0.00` | `0.00` | `0.00` | | `110` | `B:GLU 165` |  | `53.68` | `0.00` | `0.00` | | `111` | `B:GLU 166` |  | `121.44` | `0.00` | `0.00` | | `112` | `B:ALA 167` |  | `17.15` | `0.00` | `0.00` | | `113` | `B:PHE 168` |  | `0.00` | `0.00` | `0.00` | | `114` | `B:SER 169` |  | `57.01` | `0.00` | `0.00` | | `115` | `B:VAL 170` |  | `107.31` | `0.00` | `0.00` | | `116` | `B:PHE 171` |  | `18.50` | `0.00` | `0.00` | | `117` | `B:GLY 172` |  | `22.80` | `0.00` | `0.00` | | `118` | `B:GLN 173` |  | `131.30` | `0.00` | `0.00` | | `119` | `B:VAL 174` |  | `6.47` | `0.00` | `0.00` | | `120` | `B:GLU 175` |  | `13.76` | `0.00` | `0.00` | | `121` | `B:ARG 176` |  | `69.86` | `0.00` | `0.00` | | `122` | `B:ALA 177` |  | `7.85` | `0.00` | `0.00` | | `123` | `B:VAL 178` |  | `42.34` | `0.00` | `0.00` | | `124` | `B:VAL 179` |  | `14.71` | `0.00` | `0.00` | | `125` | `B:ILE 180` |  | `65.50` | `0.00` | `0.00` | | `126` | `B:VAL 181` |  | `84.18` | `0.00` | `0.00` | | `127` | `B:ASP 182` |  | `47.33` | `0.00` | `0.00` | | `128` | `B:ASP 183` |  | `139.89` | `0.00` | `0.00` | | `129` | `B:ARG 184` | `HS` | `179.71` | `85.31`  ||||| | `-0.59` | | `130` | `B:GLY 185` |  | `50.30` | `0.00` | `0.00` | | `131` | `B:ARG 186` | `S` | `137.27` | `115.80`  ||||||||| | `-0.77` | | `132` | `B:PRO 187` |  | `75.38` | `24.52`  |||| | `0.24` | | `133` | `B:SER 188` |  | `55.32` | `0.00` | `0.00` | | `134` | `B:GLY 189` |  | `21.54` | `1.68`  | | `0.03` | | `135` | `B:LYS 190` |  | `94.55` | `0.00` | `0.00` | | `136` | `B:GLY 191` |  | `0.00` | `0.00` | `0.00` | | `137` | `B:ILE 192` |  | `35.22` | `0.00` | `0.00` | | `138` | `B:VAL 193` |  | `0.00` | `0.00` | `0.00` | | `139` | `B:GLU 194` |  | `5.31` | `0.00` | `0.00` | | `140` | `B:PHE 195` |  | `0.00` | `0.00` | `0.00` | | `141` | `B:SER 196` |  | `39.97` | `0.00` | `0.00` | | `142` | `B:GLY 197` |  | `23.84` | `0.00` | `0.00` | | `143` | `B:LYS 198` |  | `117.15` | `0.00` | `0.00` | | `144` | `B:PRO 199` |  | `108.19` | `0.00` | `0.00` | | `145` | `B:ALA 200` |  | `15.29` | `0.00` | `0.00` | | `146` | `B:ALA 201` |  | `4.51` | `0.00` | `0.00` | | `147` | `B:ARG 202` |  | `150.36` | `0.00` | `0.00` | | `148` | `B:LYS 203` |  | `98.44` | `0.00` | `0.00` | | `149` | `B:ALA 204` |  | `0.00` | `0.00` | `0.00` | | `150` | `B:LEU 205` |  | `41.96` | `0.00` | `0.00` | | `151` | `B:ASP 206` |  | `66.05` | `0.00` | `0.00` | | `152` | `B:ARG 207` |  | `150.82` | `0.00` | `0.00` | | `153` | `B:CYS 208` |  | `15.27` | `0.00` | `0.00` | | `154` | `B:SER 209` |  | `92.72` | `0.00` | `0.00` | | `155` | `B:GLU 210` |  | `142.60` | `0.00` | `0.00` | | `156` | `B:GLY 211` |  | `37.54` | `0.00` | `0.00` | | `157` | `B:SER 212` |  | `84.75` | `0.00` | `0.00` | | `158` | `B:PHE 213` |  | `45.74` | `0.00` | `0.00` | | `159` | `B:LEU 214` |  | `81.75` | `0.00` | `0.00` | | `160` | `B:LEU 215` |  | `18.76` | `0.00` | `0.00` | | `161` | `B:THR 216` | `H` | `43.73` | `8.18`  || | `0.10` | | `162` | `B:THR 217` |  | `122.88` | `1.51`  | | `0.02` | | `163` | `B:PHE 218` |  | `50.32` | `34.28`  ||||||| | `0.52` | | `164` | `B:PRO 219` |  | `60.48` | `0.00` | `0.00` | | `165` | `B:ARG 220` |  | `55.90` | `35.03`  ||||||| | `-0.81` | | `166` | `B:PRO 221` |  | `57.61` | `0.00` | `0.00` | | `167` | `B:VAL 222` |  | `5.04` | `0.00` | `0.00` | | `168` | `B:THR 223` |  | `65.29` | `0.00` | `0.00` | | `169` | `B:VAL 224` |  | `16.14` | `0.00` | `0.00` | | `170` | `B:GLU 225` |  | `47.21` | `0.00` | `0.00` | | `171` | `B:PRO 226` |  | `92.52` | `0.00` | `0.00` | | `172` | `B:MET 227` |  | `46.26` | `0.00` | `0.00` | | `173` | `B:ASP 228` |  | `103.24` | `0.00` | `0.00` | | `174` | `B:GLN 229` |  | `85.49` | `0.00` | `0.00` | | `175` | `B:LEU 230` |  | `145.12` | `0.00` | `0.00` | | `176` | `B:ASP 231` |  | `88.94` | `0.00` | `0.00` | | `177` | `B:ASP 232` |  | `139.28` | `0.00` | `0.00` | | `178` | `B:GLU 233` |  | `134.56` | `0.00` | `0.00` | | `179` | `B:GLU 234` |  | `36.06` | `0.00` | `0.00` | | `180` | `B:GLY 235` |  | `46.29` | `0.00` | `0.00` | | `181` | `B:LEU 236` |  | `136.07` | `0.00` | `0.00` | | `182` | `B:PRO 237` |  | `38.50` | `0.00` | `0.00` | | `183` | `B:GLU 238` |  | `80.14` | `0.00` | `0.00` | | `184` | `B:LYS 239` |  | `151.30` | `0.00` | `0.00` | | `185` | `B:LEU 240` |  | `88.19` | `0.00` | `0.00` | | `186` | `B:VAL 241` |  | `37.87` | `0.00` | `0.00` | | `187` | `B:ILE 242` |  | `138.89` | `0.00` | `0.00` | | `188` | `B:LYS 243` |  | `125.74` | `0.00` | `0.00` | | `189` | `B:ASN 244` |  | `76.86` | `0.00` | `0.00` | | `190` | `B:GLN 245` |  | `162.18` | `0.00` | `0.00` | | `191` | `B:GLN 246` |  | `96.25` | `0.00` | `0.00` | | `192` | `B:PHE 247` |  | `30.83` | `0.00` | `0.00` | | `193` | `B:HIS 248` |  | `104.99` | `0.00` | `0.00` | | `194` | `B:LYS 249` |  | `129.57` | `0.00` | `0.00` | | `195` | `B:GLU 250` |  | `115.08` | `0.00` | `0.00` | | `196` | `B:ARG 251` |  | `135.78` | `0.00` | `0.00` | | `197` | `B:GLU 252` |  | `130.75` | `0.00` | `0.00` | | `198` | `B:GLN 253` |  | `108.49` | `0.00` | `0.00` | | `199` | `B:PRO 254` |  | `89.95` | `0.00` | `0.00` | | `200` | `B:PRO 255` |  | `142.46` | `0.00` | `0.00` | | `201` | `B:ARG 256` |  | `127.97` | `0.00` | `0.00` | | `202` | `B:PHE 257` |  | `195.87` | `0.00` | `0.00` | | `203` | `B:ALA 258` |  | `29.45` | `0.00` | `0.00` | | `204` | `B:GLN 259` |  | `130.65` | `0.00` | `0.00` | | `205` | `B:PRO 260` |  | `113.76` | `0.00` | `0.00` | | `206` | `B:GLY 261` |  | `85.39` | `0.00` | `0.00` | | `207` | `B:SER 262` |  | `37.62` | `0.00` | `0.00` | | `208` | `B:PHE 263` |  | `155.74` | `0.00` | `0.00` | | `209` | `B:GLU 264` |  | `57.57` | `0.00` | `0.00` | | `210` | `B:TYR 265` |  | `74.23` | `0.00` | `0.00` | | `211` | `B:GLU 266` |  | `85.13` | `0.00` | `0.00` | | `212` | `B:TYR 267` |  | `94.41` | `0.00` | `0.00` | | `213` | `B:ALA 268` |  | `37.59` | `0.00` | `0.00` | | `214` | `B:MET 269` |  | `88.95` | `0.00` | `0.00` | | `215` | `B:ARG 270` |  | `142.30` | `0.00` | `0.00` | | `216` | `B:TRP 271` |  | `158.00` | `0.00` | `0.00` | | `217` | `B:LYS 272` |  | `128.31` | `0.00` | `0.00` | | `218` | `B:ALA 273` |  | `63.66` | `0.00` | `0.00` | | `219` | `B:LEU 274` |  | `86.77` | `0.00` | `0.00` | | `220` | `B:ILE 275` |  | `102.34` | `0.00` | `0.00` | | `221` | `B:GLU 276` |  | `77.09` | `4.47`  | | `0.06` | | `222` | `B:MET 277` |  | `84.74` | `0.00` | `0.00` | | `223` | `B:GLU 278` |  | `109.31` | `0.00` | `0.00` | | `224` | `B:LYS 279` |  | `112.73` | `0.00` | `0.00` | | `225` | `B:GLN 280` |  | `103.31` | `18.61`  || | `-0.22` | | `226` | `B:GLN 281` |  | `105.30` | `0.00` | `0.00` | | `227` | `B:GLN 282` |  | `95.85` | `0.00` | `0.00` | | `228` | `B:ASP 283` |  | `64.49` | `0.00` | `0.00` | | `229` | `B:GLN 284` |  | `102.38` | `0.00` | `0.00` | | `230` | `B:VAL 285` |  | `71.34` | `0.00` | `0.00` | | `231` | `B:ASP 286` |  | `72.52` | `0.00` | `0.00` | | `232` | `B:ARG 287` |  | `146.22` | `15.74`  || | `-0.58` | | `233` | `B:ASN 288` |  | `83.54` | `0.00` | `0.00` | | `234` | `B:ILE 289` |  | `81.10` | `0.00` | `0.00` | | `235` | `B:LYS 290` | `H` | `116.03` | `61.38`  |||||| | `-0.55` | | `236` | `B:GLU 291` | `H` | `113.43` | `23.64`  ||| | `0.27` | | `237` | `B:ALA 292` |  | `61.65` | `0.00` | `0.00` | | `238` | `B:ARG 293` |  | `137.72` | `10.94`  | | `0.12` | | `239` | `B:GLU 294` | `S` | `80.43` | `79.37`  |||||||||| | `0.01` | | `240` | `B:LYS 295` |  | `120.43` | `11.00`  | | `0.17` | | `241` | `B:LEU 296` |  | `97.57` | `0.00` | `0.00` | | `242` | `B:GLU 297` |  | `54.45` | `46.08`  ||||||||| | `-0.19` | | `243` | `B:MET 298` | `H` | `130.51` | `113.50`  ||||||||| | `2.87` | | `244` | `B:GLU 299` |  | `85.15` | `0.00` | `0.00` | | `245` | `B:MET 300` |  | `50.56` | `0.00` | `0.00` | | `246` | `B:GLU 301` |  | `67.49` | `35.21`  |||||| | `0.56` | | `247` | `B:ALA 302` |  | `64.91` | `19.02`  ||| | `0.26` | | `248` | `B:ALA 303` |  | `56.96` | `0.00` | `0.00` | | `249` | `B:ARG 304` |  | `122.54` | `0.00` | `0.00` | | `250` | `B:HIS 305` | `HS` | `116.73` | `89.71`  |||||||| | `0.98` | | `251` | `B:GLU 306` |  | `115.52` | `0.00` | `0.00` | | `252` | `B:HIS 307` |  | `104.09` | `0.00` | `0.00` | | `253` | `B:GLN 308` |  | `102.62` | `2.82`  | | `-0.05` | | `254` | `B:VAL 309` | `H` | `75.55` | `37.20`  ||||| | `0.59` | | `255` | `B:MET 310` |  | `119.85` | `0.00` | `0.00` | | `256` | `B:LEU 311` |  | `96.07` | `0.00` | `0.00` | | `257` | `B:MET 312` |  | `132.03` | `65.64`  ||||| | `1.11` | | `258` | `B:ARG 313` |  | `171.65` | `5.61`  | | `0.07` | | `259` | `B:GLN 314` |  | `126.91` | `0.00` | `0.00` | | `260` | `B:ASP 315` |  | `60.47` | `0.00` | `0.00` | | `261` | `B:LEU 316` |  | `100.91` | `46.87`  ||||| | `0.75` | | `262` | `B:MET 317` |  | `115.45` | `0.00` | `0.00` | | `263` | `B:ARG 318` |  | `162.88` | `0.00` | `0.00` | | `264` | `B:ARG 319` |  | `140.23` | `6.47`  | | `-0.10` | | `265` | `B:GLN 320` |  | `95.52` | `0.00` | `0.00` | | `266` | `B:GLU 321` |  | `69.20` | `0.00` | `0.00` | | `267` | `B:GLU 322` |  | `78.44` | `0.00` | `0.00` | | `268` | `B:LEU 323` |  | `99.51` | `0.00` | `0.00` | | `269` | `B:ARG 324` |  | `146.75` | `0.00` | `0.00` | | `270` | `B:ARG 325` |  | `144.39` | `0.00` | `0.00` | | `271` | `B:MET 326` |  | `122.28` | `0.00` | `0.00` | | `272` | `B:GLU 327` |  | `117.21` | `0.00` | `0.00` | | `273` | `B:GLU 328` |  | `76.89` | `0.00` | `0.00` | | `274` | `B:LEU 329` |  | `118.69` | `0.00` | `0.00` | | `275` | `B:HIS 330` |  | `120.06` | `0.00` | `0.00` | | `276` | `B:ASN 331` |  | `80.37` | `0.00` | `0.00` | | `277` | `B:GLN 332` |  | `94.61` | `0.00` | `0.00` | | `278` | `B:GLU 333` |  | `78.46` | `0.00` | `0.00` | | `279` | `B:VAL 334` |  | `52.83` | `0.00` | `0.00` | | `280` | `B:GLN 335` |  | `93.47` | `0.00` | `0.00` | | `281` | `B:LYS 336` |  | `138.39` | `0.00` | `0.00` | | `282` | `B:ARG 337` |  | `161.45` | `0.00` | `0.00` | | `283` | `B:LYS 338` |  | `130.60` | `0.00` | `0.00` | | `284` | `B:GLN 339` |  | `106.70` | `0.00` | `0.00` | | `285` | `B:LEU 340` |  | `107.73` | `0.00` | `0.00` | | `286` | `B:GLU 341` |  | `118.34` | `0.00` | `0.00` | | `287` | `B:LEU 342` |  | `114.28` | `0.00` | `0.00` | | `288` | `B:ARG 343` |  | `163.03` | `0.00` | `0.00` | | `289` | `B:GLN 344` |  | `110.09` | `0.00` | `0.00` | | `290` | `B:GLU 345` |  | `66.50` | `0.00` | `0.00` | | `291` | `B:GLU 346` |  | `90.70` | `0.00` | `0.00` | | `292` | `B:GLU 347` |  | `85.10` | `0.00` | `0.00` | | `293` | `B:ARG 348` |  | `122.00` | `0.00` | `0.00` | | `294` | `B:ARG 349` |  | `142.83` | `0.00` | `0.00` | | `295` | `B:ARG 350` |  | `177.75` | `0.00` | `0.00` | | `296` | `B:ARG 351` |  | `148.99` | `0.00` | `0.00` | | `297` | `B:GLU 352` |  | `90.98` | `0.00` | `0.00` | | `298` | `B:GLU 353` |  | `99.61` | `0.00` | `0.00` | | `299` | `B:GLU 354` |  | `103.36` | `0.00` | `0.00` | | `300` | `B:MET 355` |  | `129.53` | `0.00` | `0.00` | | `301` | `B:ARG 356` |  | `161.40` | `0.00` | `0.00` | | `302` | `B:ARG 357` |  | `175.10` | `0.00` | `0.00` | | `303` | `B:GLN 358` |  | `104.50` | `0.00` | `0.00` | | `304` | `B:GLN 359` |  | `120.62` | `0.00` | `0.00` | | `305` | `B:GLU 360` |  | `87.60` | `0.00` | `0.00` | | `306` | `B:GLU 361` |  | `99.11` | `0.00` | `0.00` | | `307` | `B:MET 362` |  | `125.91` | `0.00` | `0.00` | | `308` | `B:MET 363` |  | `109.19` | `0.00` | `0.00` | | `309` | `B:ARG 364` |  | `153.17` | `0.00` | `0.00` | | `310` | `B:ARG 365` |  | `186.57` | `0.00` | `0.00` | | `311` | `B:GLN 366` |  | `129.70` | `0.00` | `0.00` | | `312` | `B:GLN 367` |  | `116.88` | `0.00` | `0.00` | | `313` | `B:GLU 368` |  | `116.19` | `0.00` | `0.00` | | `314` | `B:GLY 369` |  | `69.02` | `0.00` | `0.00` | | `315` | `B:PHE 370` |  | `231.98` | `0.00` | `0.00` | |  | | ## | **Structure 2** | **HSDC** | **ASA** | **BSA** | **ΔiG** | | --- | --- | --- | --- | --- | --- | | `1` | `A:ILE  41` |  | `140.98` | `0.00` | `0.00` | | `2` | `A:VAL  42` |  | `99.02` | `0.00` | `0.00` | | `3` | `A:LYS  43` | `H` | `61.86` | `1.05`  | | `-0.00` | | `4` | `A:GLU  44` | `S` | `111.18` | `30.07`  ||| | `-0.15` | | `5` | `A:ASN  45` |  | `19.53` | `0.00` | `0.00` | | `6` | `A:LYS  46` |  | `174.36` | `0.00` | `0.00` | | `7` | `A:LEU  47` |  | `51.31` | `0.00` | `0.00` | | `8` | `A:PHE  48` |  | `0.31` | `0.00` | `0.00` | | `9` | `A:GLU  49` |  | `41.10` | `21.72`  |||||| | `-0.35` | | `10` | `A:HIS  50` |  | `89.78` | `0.00` | `0.00` | | `11` | `A:TYR  51` |  | `0.85` | `0.00` | `0.00` | | `12` | `A:TYR  52` |  | `0.49` | `0.00` | `0.00` | | `13` | `A:GLN  53` |  | `89.13` | `44.30`  ||||| | `-0.02` | | `14` | `A:GLU  54` | `H` | `66.07` | `4.05`  | | `0.01` | | `15` | `A:LEU  55` |  | `18.40` | `0.00` | `0.00` | | `16` | `A:LYS  56` |  | `144.53` | `30.40`  ||| | `0.24` | | `17` | `A:ILE  57` |  | `7.81` | `0.00` | `0.00` | | `18` | `A:VAL  58` | `H` | `11.28` | `8.16`  |||||||| | `-0.06` | | `19` | `A:PRO  59` | `H` | `36.54` | `25.74`  |||||||| | `0.38` | | `20` | `A:GLU  60` | `H` | `157.10` | `138.24`  ||||||||| | `0.03` | | `21` | `A:GLY  61` |  | `76.50` | `46.56`  ||||||| | `0.40` | | `22` | `A:GLU  62` |  | `48.16` | `47.23`  |||||||||| | `-0.13` | | `23` | `A:TRP  63` |  | `49.29` | `43.90`  ||||||||| | `0.55` | | `24` | `A:GLY  64` |  | `41.32` | `13.67`  |||| | `0.18` | | `25` | `A:GLN  65` |  | `111.20` | `61.60`  |||||| | `-0.45` | | `26` | `A:PHE  66` |  | `0.00` | `0.00` | `0.00` | | `27` | `A:MET  67` |  | `4.86` | `4.86`  |||||||||| | `0.08` | | `28` | `A:ASP  68` | `S` | `76.57` | `6.14`  | | `-0.07` | | `29` | `A:ALA  69` |  | `13.47` | `2.33`  || | `0.04` | | `30` | `A:LEU  70` |  | `0.61` | `0.00` | `0.00` | | `31` | `A:ARG  71` |  | `85.50` | `53.94`  ||||||| | `-0.45` | | `32` | `A:GLU  72` |  | `99.74` | `0.00` | `0.00` | | `33` | `A:PRO  73` |  | `104.02` | `0.00` | `0.00` | | `34` | `A:LEU  74` |  | `39.64` | `0.00` | `0.00` | | `35` | `A:PRO  75` |  | `16.24` | `0.00` | `0.00` | | `36` | `A:ALA  76` |  | `39.07` | `0.00` | `0.00` | | `37` | `A:THR  77` |  | `17.05` | `0.00` | `0.00` | | `38` | `A:LEU  78` |  | `8.70` | `0.00` | `0.00` | | `39` | `A:ARG  79` |  | `3.49` | `0.00` | `0.00` | | `40` | `A:ILE  80` |  | `3.93` | `0.00` | `0.00` | | `41` | `A:THR  81` |  | `4.13` | `0.00` | `0.00` | | `42` | `A:GLY  82` |  | `35.04` | `0.00` | `0.00` | | `43` | `A:TYR  83` |  | `60.11` | `0.00` | `0.00` | | `44` | `A:LYS  84` |  | `97.94` | `0.00` | `0.00` | | `45` | `A:SER  85` |  | `49.42` | `0.00` | `0.00` | | `46` | `A:HIS  86` |  | `18.65` | `0.00` | `0.00` | | `47` | `A:ALA  87` |  | `3.34` | `0.00` | `0.00` | | `48` | `A:LYS  88` |  | `128.13` | `0.00` | `0.00` | | `49` | `A:GLU  89` |  | `55.23` | `0.00` | `0.00` | | `50` | `A:ILE  90` |  | `5.19` | `0.00` | `0.00` | | `51` | `A:LEU  91` |  | `23.27` | `0.00` | `0.00` | | `52` | `A:HIS  92` |  | `104.78` | `0.00` | `0.00` | | `53` | `A:CYS  93` |  | `25.97` | `0.00` | `0.00` | | `54` | `A:LEU  94` |  | `0.00` | `0.00` | `0.00` | | `55` | `A:LYS  95` |  | `67.58` | `0.00` | `0.00` | | `56` | `A:ASN  96` |  | `65.88` | `0.00` | `0.00` | | `57` | `A:LYS  97` |  | `63.16` | `0.00` | `0.00` | | `58` | `A:TYR  98` |  | `12.97` | `0.00` | `0.00` | | `59` | `A:PHE  99` |  | `8.01` | `0.00` | `0.00` | | `60` | `A:LYS 100` |  | `115.30` | `0.00` | `0.00` | | `61` | `A:GLU 101` |  | `100.30` | `0.00` | `0.00` | | `62` | `A:LEU 102` |  | `4.51` | `0.00` | `0.00` | | `63` | `A:GLU 103` |  | `87.57` | `0.00` | `0.00` | | `64` | `A:ASP 104` |  | `125.59` | `0.00` | `0.00` | | `65` | `A:LEU 105` |  | `25.85` | `0.00` | `0.00` | | `66` | `A:GLU 106` |  | `114.05` | `0.00` | `0.00` | | `67` | `A:VAL 107` |  | `21.35` | `0.00` | `0.00` | | `68` | `A:ASP 108` |  | `141.51` | `0.00` | `0.00` | | `69` | `A:GLY 109` |  | `72.00` | `4.54`  | | `-0.05` | | `70` | `A:GLN 110` |  | `131.57` | `0.00` | `0.00` | | `71` | `A:LYS 111` |  | `127.47` | `18.09`  || | `0.23` | | `72` | `A:VAL 112` |  | `7.90` | `0.00` | `0.00` | | `73` | `A:GLU 113` |  | `80.78` | `13.49`  || | `-0.02` | | `74` | `A:VAL 114` |  | `29.46` | `0.00` | `0.00` | | `75` | `A:PRO 115` |  | `1.10` | `0.00` | `0.00` | | `76` | `A:GLN 116` |  | `75.24` | `0.00` | `0.00` | | `77` | `A:PRO 117` |  | `45.99` | `0.00` | `0.00` | | `78` | `A:LEU 118` |  | `15.88` | `0.00` | `0.00` | | `79` | `A:SER 119` |  | `84.76` | `0.00` | `0.00` | | `80` | `A:TRP 120` |  | `14.39` | `0.00` | `0.00` | | `81` | `A:TYR 121` |  | `21.44` | `0.00` | `0.00` | | `82` | `A:PRO 122` |  | `62.58` | `0.00` | `0.00` | | `83` | `A:GLU 123` |  | `103.04` | `0.00` | `0.00` | | `84` | `A:GLU 124` |  | `73.54` | `0.00` | `0.00` | | `85` | `A:LEU 125` |  | `35.04` | `0.00` | `0.00` | | `86` | `A:ALA 126` |  | `0.00` | `0.00` | `0.00` | | `87` | `A:TRP 127` |  | `15.45` | `0.00` | `0.00` | | `88` | `A:HIS 128` |  | `43.95` | `0.00` | `0.00` | | `89` | `A:THR 129` |  | `6.69` | `0.00` | `0.00` | | `90` | `A:ASN 130` |  | `80.05` | `1.17`  | | `-0.01` | | `91` | `A:LEU 131` |  | `8.49` | `0.00` | `0.00` | | `92` | `A:SER 132` |  | `46.86` | `0.00` | `0.00` | | `93` | `A:ARG 133` |  | `194.40` | `0.00` | `0.00` | | `94` | `A:LYS 134` |  | `154.51` | `0.00` | `0.00` | | `95` | `A:ILE 135` |  | `56.47` | `0.00` | `0.00` | | `96` | `A:LEU 136` |  | `12.58` | `0.00` | `0.00` | | `97` | `A:ARG 137` |  | `171.87` | `0.00` | `0.00` | | `98` | `A:LYS 138` |  | `163.56` | `0.00` | `0.00` | | `99` | `A:SER 139` |  | `7.30` | `0.00` | `0.00` | | `100` | `A:PRO 140` |  | `112.08` | `0.00` | `0.00` | | `101` | `A:HIS 141` |  | `130.76` | `0.00` | `0.00` | | `102` | `A:LEU 142` |  | `0.33` | `0.00` | `0.00` | | `103` | `A:GLU 143` |  | `63.60` | `0.00` | `0.00` | | `104` | `A:LYS 144` |  | `91.05` | `0.00` | `0.00` | | `105` | `A:PHE 145` |  | `6.65` | `0.00` | `0.00` | | `106` | `A:HIS 146` |  | `33.45` | `0.00` | `0.00` | | `107` | `A:GLN 147` |  | `70.83` | `0.00` | `0.00` | | `108` | `A:PHE 148` |  | `2.65` | `0.00` | `0.00` | | `109` | `A:LEU 149` |  | `11.05` | `0.00` | `0.00` | | `110` | `A:VAL 150` |  | `54.05` | `0.00` | `0.00` | | `111` | `A:SER 151` |  | `9.50` | `0.00` | `0.00` | | `112` | `A:GLU 152` |  | `1.22` | `0.00` | `0.00` | | `113` | `A:THR 153` |  | `23.72` | `0.00` | `0.00` | | `114` | `A:GLU 154` |  | `83.91` | `0.00` | `0.00` | | `115` | `A:SER 155` |  | `23.36` | `0.00` | `0.00` | | `116` | `A:GLY 156` |  | `8.94` | `0.00` | `0.00` | | `117` | `A:ASN 157` |  | `16.84` | `0.00` | `0.00` | | `118` | `A:ILE 158` |  | `2.75` | `0.00` | `0.00` | | `119` | `A:SER 159` |  | `15.94` | `0.00` | `0.00` | | `120` | `A:ARG 160` |  | `124.50` | `0.00` | `0.00` | | `121` | `A:GLN 161` |  | `7.20` | `0.00` | `0.00` | | `122` | `A:GLU 162` |  | `36.41` | `0.00` | `0.00` | | `123` | `A:ALA 163` |  | `7.16` | `0.00` | `0.00` | | `124` | `A:VAL 164` |  | `3.18` | `0.00` | `0.00` | | `125` | `A:SER 165` |  | `12.81` | `0.00` | `0.00` | | `126` | `A:MET 166` |  | `0.00` | `0.00` | `0.00` | | `127` | `A:ILE 167` |  | `0.49` | `0.00` | `0.00` | | `128` | `A:PRO 168` |  | `0.00` | `0.00` | `0.00` | | `129` | `A:PRO 169` |  | `0.00` | `0.00` | `0.00` | | `130` | `A:LEU 170` |  | `25.19` | `0.00` | `0.00` | | `131` | `A:LEU 171` |  | `0.67` | `0.00` | `0.00` | | `132` | `A:LEU 172` |  | `0.00` | `0.00` | `0.00` | | `133` | `A:ASN 173` |  | `49.76` | `0.00` | `0.00` | | `134` | `A:VAL 174` |  | `8.89` | `0.00` | `0.00` | | `135` | `A:ARG 175` |  | `127.23` | `0.00` | `0.00` | | `136` | `A:PRO 176` |  | `39.62` | `0.00` | `0.00` | | `137` | `A:HIS 177` |  | `98.24` | `0.00` | `0.00` | | `138` | `A:HIS 178` |  | `11.75` | `0.00` | `0.00` | | `139` | `A:LYS 179` |  | `46.19` | `0.00` | `0.00` | | `140` | `A:ILE 180` |  | `0.00` | `0.00` | `0.00` | | `141` | `A:LEU 181` |  | `0.00` | `0.00` | `0.00` | | `142` | `A:ASP 182` |  | `0.89` | `0.00` | `0.00` | | `143` | `A:MET 183` |  | `0.00` | `0.00` | `0.00` | | `144` | `A:CYS 184` |  | `24.19` | `0.00` | `0.00` | | `145` | `A:ALA 185` |  | `6.23` | `0.00` | `0.00` | | `146` | `A:ALA 186` |  | `7.31` | `0.00` | `0.00` | | `147` | `A:PRO 187` |  | `79.20` | `0.00` | `0.00` | | `148` | `A:GLY 188` |  | `0.65` | `0.00` | `0.00` | | `149` | `A:SER 189` |  | `24.87` | `0.00` | `0.00` | | `150` | `A:LYS 190` |  | `19.50` | `0.00` | `0.00` | | `151` | `A:THR 191` |  | `0.00` | `0.00` | `0.00` | | `152` | `A:THR 192` |  | `9.93` | `0.00` | `0.00` | | `153` | `A:GLN 193` |  | `2.03` | `0.00` | `0.00` | | `154` | `A:LEU 194` |  | `0.00` | `0.00` | `0.00` | | `155` | `A:ILE 195` |  | `2.17` | `0.00` | `0.00` | | `156` | `A:GLU 196` |  | `31.43` | `0.00` | `0.00` | | `157` | `A:MET 197` |  | `38.69` | `0.00` | `0.00` | | `158` | `A:LEU 198` |  | `1.10` | `0.00` | `0.00` | | `159` | `A:HIS 199` |  | `25.72` | `0.00` | `0.00` | | `160` | `A:ALA 200` |  | `47.61` | `0.00` | `0.00` | | `161` | `A:ASP 201` |  | `58.41` | `0.00` | `0.00` | | `162` | `A:MET 202` |  | `86.02` | `0.00` | `0.00` | | `163` | `A:ASN 203` |  | `134.68` | `0.00` | `0.00` | | `164` | `A:VAL 204` |  | `60.42` | `0.00` | `0.00` | | `165` | `A:PRO 205` |  | `57.12` | `0.00` | `0.00` | | `166` | `A:PHE 206` |  | `44.55` | `0.00` | `0.00` | | `167` | `A:PRO 207` |  | `1.78` | `0.00` | `0.00` | | `168` | `A:GLU 208` |  | `106.37` | `0.00` | `0.00` | | `169` | `A:GLY 209` |  | `0.00` | `0.00` | `0.00` | | `170` | `A:PHE 210` |  | `1.41` | `0.00` | `0.00` | | `171` | `A:VAL 211` |  | `0.00` | `0.00` | `0.00` | | `172` | `A:ILE 212` |  | `0.49` | `0.00` | `0.00` | | `173` | `A:ALA 213` |  | `0.00` | `0.00` | `0.00` | | `174` | `A:ASN 214` |  | `0.29` | `0.00` | `0.00` | | `175` | `A:ASP 215` |  | `6.71` | `0.00` | `0.00` | | `176` | `A:VAL 216` |  | `96.87` | `0.00` | `0.00` | | `177` | `A:ASP 217` |  | `54.09` | `0.00` | `0.00` | | `178` | `A:ASN 218` |  | `44.44` | `0.00` | `0.00` | | `179` | `A:LYS 219` |  | `163.80` | `0.00` | `0.00` | | `180` | `A:ARG 220` |  | `104.42` | `0.00` | `0.00` | | `181` | `A:CYS 221` |  | `0.00` | `0.00` | `0.00` | | `182` | `A:TYR 222` |  | `94.97` | `0.00` | `0.00` | | `183` | `A:LEU 223` |  | `104.38` | `0.00` | `0.00` | | `184` | `A:LEU 224` |  | `0.16` | `0.00` | `0.00` | | `185` | `A:VAL 225` |  | `11.56` | `0.00` | `0.00` | | `186` | `A:HIS 226` |  | `105.40` | `0.00` | `0.00` | | `187` | `A:GLN 227` |  | `55.71` | `0.00` | `0.00` | | `188` | `A:ALA 228` |  | `5.86` | `0.00` | `0.00` | | `189` | `A:LYS 229` |  | `110.50` | `0.00` | `0.00` | | `190` | `A:ARG 230` |  | `65.67` | `0.00` | `0.00` | | `191` | `A:LEU 231` |  | `27.27` | `0.00` | `0.00` | | `192` | `A:SER 232` |  | `30.64` | `0.00` | `0.00` | | `193` | `A:SER 233` |  | `3.19` | `0.00` | `0.00` | | `194` | `A:PRO 234` |  | `1.67` | `0.00` | `0.00` | | `195` | `A:CYS 235` |  | `0.17` | `0.00` | `0.00` | | `196` | `A:ILE 236` |  | `6.33` | `0.00` | `0.00` | | `197` | `A:MET 237` |  | `0.00` | `0.00` | `0.00` | | `198` | `A:VAL 238` |  | `0.67` | `0.00` | `0.00` | | `199` | `A:VAL 239` |  | `1.00` | `0.00` | `0.00` | | `200` | `A:ASN 240` |  | `9.59` | `0.00` | `0.00` | | `201` | `A:HIS 241` |  | `14.83` | `0.00` | `0.00` | | `202` | `A:ASP 242` |  | `49.45` | `0.00` | `0.00` | | `203` | `A:ALA 243` |  | `5.33` | `0.00` | `0.00` | | `204` | `A:SER 244` |  | `26.17` | `0.00` | `0.00` | | `205` | `A:SER 245` |  | `71.06` | `0.00` | `0.00` | | `206` | `A:ILE 246` |  | `7.03` | `0.00` | `0.00` | | `207` | `A:PRO 247` |  | `32.29` | `0.00` | `0.00` | | `208` | `A:ARG 248` |  | `101.42` | `0.00` | `0.00` | | `209` | `A:LEU 249` |  | `5.59` | `0.00` | `0.00` | | `210` | `A:GLN 250` |  | `55.44` | `0.00` | `0.00` | | `211` | `A:ILE 251` |  | `16.56` | `0.00` | `0.00` | | `212` | `A:ASP 252` |  | `54.55` | `0.00` | `0.00` | | `213` | `A:VAL 253` |  | `60.95` | `0.00` | `0.00` | | `214` | `A:ASP 254` |  | `144.49` | `0.00` | `0.00` | | `215` | `A:GLY 255` |  | `65.52` | `0.00` | `0.00` | | `216` | `A:ARG 256` |  | `180.93` | `0.00` | `0.00` | | `217` | `A:LYS 257` |  | `139.31` | `0.00` | `0.00` | | `218` | `A:GLU 258` |  | `82.07` | `0.00` | `0.00` | | `219` | `A:ILE 259` |  | `75.85` | `0.00` | `0.00` | | `220` | `A:LEU 260` |  | `2.14` | `0.00` | `0.00` | | `221` | `A:PHE 261` |  | `82.12` | `0.00` | `0.00` | | `222` | `A:TYR 262` |  | `2.39` | `0.00` | `0.00` | | `223` | `A:ASP 263` |  | `37.75` | `0.00` | `0.00` | | `224` | `A:ARG 264` |  | `30.89` | `0.00` | `0.00` | | `225` | `A:ILE 265` |  | `0.00` | `0.00` | `0.00` | | `226` | `A:LEU 266` |  | `0.17` | `0.00` | `0.00` | | `227` | `A:CYS 267` |  | `0.00` | `0.00` | `0.00` | | `228` | `A:ASP 268` |  | `30.57` | `0.00` | `0.00` | | `229` | `A:VAL 269` |  | `5.90` | `0.00` | `0.00` | | `230` | `A:PRO 270` |  | `60.00` | `0.00` | `0.00` | | `231` | `A:CYS 271` |  | `24.53` | `0.00` | `0.00` | | `232` | `A:SER 272` |  | `19.56` | `0.00` | `0.00` | | `233` | `A:GLY 273` |  | `8.53` | `0.00` | `0.00` | | `234` | `A:ASP 274` |  | `0.00` | `0.00` | `0.00` | | `235` | `A:GLY 275` |  | `0.77` | `0.00` | `0.00` | | `236` | `A:THR 276` |  | `10.67` | `0.00` | `0.00` | | `237` | `A:MET 277` |  | `1.46` | `0.00` | `0.00` | | `238` | `A:ARG 278` |  | `15.75` | `0.00` | `0.00` | | `239` | `A:LYS 279` |  | `110.82` | `0.00` | `0.00` | | `240` | `A:ASN 280` |  | `65.87` | `0.00` | `0.00` | | `241` | `A:ILE 281` |  | `61.83` | `0.00` | `0.00` | | `242` | `A:ASP 282` |  | `74.00` | `0.00` | `0.00` | | `243` | `A:VAL 283` |  | `13.11` | `0.00` | `0.00` | | `244` | `A:TRP 284` |  | `0.76` | `0.00` | `0.00` | | `245` | `A:LYS 285` |  | `137.20` | `0.00` | `0.00` | | `246` | `A:LYS 286` |  | `132.74` | `0.00` | `0.00` | | `247` | `A:TRP 287` |  | `5.15` | `0.00` | `0.00` | | `248` | `A:THR 288` |  | `36.30` | `0.00` | `0.00` | | `249` | `A:THR 289` |  | `1.46` | `0.00` | `0.00` | | `250` | `A:LEU 290` |  | `41.88` | `0.00` | `0.00` | | `251` | `A:ASN 291` |  | `42.57` | `0.00` | `0.00` | | `252` | `A:SER 292` |  | `1.12` | `0.00` | `0.00` | | `253` | `A:LEU 293` |  | `7.73` | `0.00` | `0.00` | | `254` | `A:GLN 294` |  | `124.05` | `0.00` | `0.00` | | `255` | `A:LEU 295` |  | `40.99` | `0.00` | `0.00` | | `256` | `A:HIS 296` |  | `0.30` | `0.00` | `0.00` | | `257` | `A:GLY 297` |  | `6.41` | `0.00` | `0.00` | | `258` | `A:LEU 298` |  | `48.70` | `0.00` | `0.00` | | `259` | `A:GLN 299` |  | `0.16` | `0.00` | `0.00` | | `260` | `A:LEU 300` |  | `28.76` | `0.00` | `0.00` | | `261` | `A:ARG 301` |  | `101.42` | `0.00` | `0.00` | | `262` | `A:ILE 302` |  | `3.85` | `0.00` | `0.00` | | `263` | `A:ALA 303` |  | `0.00` | `0.00` | `0.00` | | `264` | `A:THR 304` |  | `13.71` | `0.00` | `0.00` | | `265` | `A:ARG 305` |  | `32.01` | `0.00` | `0.00` | | `266` | `A:GLY 306` |  | `0.00` | `0.00` | `0.00` | | `267` | `A:ALA 307` |  | `0.00` | `0.00` | `0.00` | | `268` | `A:GLU 308` |  | `25.50` | `0.00` | `0.00` | | `269` | `A:GLN 309` |  | `1.63` | `0.00` | `0.00` | | `270` | `A:LEU 310` |  | `0.24` | `0.00` | `0.00` | | `271` | `A:ALA 311` |  | `19.73` | `0.00` | `0.00` | | `272` | `A:GLU 312` |  | `109.54` | `0.00` | `0.00` | | `273` | `A:GLY 313` |  | `58.00` | `0.00` | `0.00` | | `274` | `A:GLY 314` |  | `1.96` | `0.00` | `0.00` | | `275` | `A:ARG 315` |  | `31.98` | `0.00` | `0.00` | | `276` | `A:MET 316` |  | `0.00` | `0.00` | `0.00` | | `277` | `A:VAL 317` |  | `0.00` | `0.00` | `0.00` | | `278` | `A:TYR 318` |  | `0.00` | `0.00` | `0.00` | | `279` | `A:SER 319` |  | `0.00` | `0.00` | `0.00` | | `280` | `A:THR 320` |  | `0.00` | `0.00` | `0.00` | | `281` | `A:CYS 321` |  | `37.99` | `0.00` | `0.00` | | `282` | `A:SER 322` |  | `0.00` | `0.00` | `0.00` | | `283` | `A:LEU 323` |  | `0.84` | `0.00` | `0.00` | | `284` | `A:ASN 324` |  | `1.16` | `0.00` | `0.00` | | `285` | `A:PRO 325` |  | `0.00` | `0.00` | `0.00` | | `286` | `A:ILE 326` |  | `1.51` | `0.00` | `0.00` | | `287` | `A:GLU 327` |  | `0.00` | `0.00` | `0.00` | | `288` | `A:ASP 328` |  | `0.00` | `0.00` | `0.00` | | `289` | `A:GLU 329` |  | `0.00` | `0.00` | `0.00` | | `290` | `A:ALA 330` |  | `0.00` | `0.00` | `0.00` | | `291` | `A:VAL 331` |  | `0.00` | `0.00` | `0.00` | | `292` | `A:ILE 332` |  | `0.00` | `0.00` | `0.00` | | `293` | `A:ALA 333` |  | `2.14` | `0.00` | `0.00` | | `294` | `A:SER 334` |  | `12.90` | `0.00` | `0.00` | | `295` | `A:LEU 335` |  | `0.00` | `0.00` | `0.00` | | `296` | `A:LEU 336` |  | `13.20` | `0.00` | `0.00` | | `297` | `A:GLU 337` |  | `93.84` | `0.00` | `0.00` | | `298` | `A:LYS 338` |  | `86.63` | `0.00` | `0.00` | | `299` | `A:SER 339` |  | `0.86` | `0.00` | `0.00` | | `300` | `A:GLU 340` |  | `142.86` | `0.00` | `0.00` | | `301` | `A:GLY 341` |  | `58.75` | `0.00` | `0.00` | | `302` | `A:ALA 342` |  | `4.47` | `0.00` | `0.00` | | `303` | `A:LEU 343` |  | `6.50` | `0.00` | `0.00` | | `304` | `A:GLU 344` |  | `64.36` | `0.00` | `0.00` | | `305` | `A:LEU 345` |  | `2.62` | `0.00` | `0.00` | | `306` | `A:ALA 346` |  | `13.51` | `0.00` | `0.00` | | `307` | `A:ASP 347` |  | `88.21` | `0.00` | `0.00` | | `308` | `A:VAL 348` |  | `2.33` | `0.00` | `0.00` | | `309` | `A:SER 349` |  | `54.18` | `0.00` | `0.00` | | `310` | `A:ASN 350` |  | `139.94` | `0.00` | `0.00` | | `311` | `A:GLU 351` |  | `53.11` | `0.00` | `0.00` | | `312` | `A:LEU 352` |  | `21.25` | `0.00` | `0.00` | | `313` | `A:PRO 353` |  | `107.31` | `0.00` | `0.00` | | `314` | `A:GLY 354` |  | `45.32` | `0.00` | `0.00` | | `315` | `A:LEU 355` |  | `16.87` | `0.00` | `0.00` | | `316` | `A:LYS 356` | `H` | `112.45` | `75.32`  ||||||| | `-0.70` | | `317` | `A:TRP 357` | `H` | `75.14` | `16.56`  ||| | `-0.11` | | `318` | `A:MET 358` |  | `59.83` | `48.96`  ||||||||| | `1.28` | | `319` | `A:PRO 359` |  | `62.08` | `48.51`  |||||||| | `0.78` | | `320` | `A:GLY 360` |  | `7.73` | `3.07`  |||| | `-0.03` | | `321` | `A:ILE 361` |  | `36.74` | `0.00` | `0.00` | | `322` | `A:THR 362` |  | `52.85` | `21.77`  ||||| | `0.18` | | `323` | `A:GLN 363` |  | `120.02` | `0.00` | `0.00` | | `324` | `A:TRP 364` |  | `11.93` | `0.00` | `0.00` | | `325` | `A:LYS 365` |  | `65.90` | `0.00` | `0.00` | | `326` | `A:VAL 366` |  | `0.00` | `0.00` | `0.00` | | `327` | `A:MET 367` |  | `3.31` | `0.00` | `0.00` | | `328` | `A:THR 368` |  | `3.68` | `0.00` | `0.00` | | `329` | `A:LYS 369` |  | `125.22` | `0.00` | `0.00` | | `330` | `A:ASP 370` |  | `100.61` | `0.00` | `0.00` | | `331` | `A:GLY 371` |  | `24.66` | `0.00` | `0.00` | | `332` | `A:GLN 372` |  | `80.01` | `0.00` | `0.00` | | `333` | `A:TRP 373` |  | `61.24` | `0.00` | `0.00` | | `334` | `A:PHE 374` |  | `8.09` | `0.00` | `0.00` | | `335` | `A:THR 375` |  | `91.77` | `0.00` | `0.00` | | `336` | `A:ASP 376` |  | `41.44` | `0.00` | `0.00` | | `337` | `A:TRP 377` |  | `49.58` | `0.00` | `0.00` | | `338` | `A:ASP 378` |  | `113.63` | `0.00` | `0.00` | | `339` | `A:ALA 379` |  | `60.51` | `0.00` | `0.00` | | `340` | `A:VAL 380` |  | `7.39` | `0.00` | `0.00` | | `341` | `A:PRO 381` |  | `57.09` | `0.00` | `0.00` | | `342` | `A:HIS 382` |  | `158.43` | `0.00` | `0.00` | | `343` | `A:SER 383` |  | `96.24` | `0.00` | `0.00` | | `344` | `A:ARG 384` |  | `54.44` | `0.00` | `0.00` | | `345` | `A:HIS 385` |  | `48.15` | `0.00` | `0.00` | | `346` | `A:THR 386` |  | `102.59` | `0.00` | `0.00` | | `347` | `A:GLN 387` |  | `88.22` | `0.00` | `0.00` | | `348` | `A:ILE 388` |  | `0.50` | `0.00` | `0.00` | | `349` | `A:ARG 389` |  | `87.99` | `0.00` | `0.00` | | `350` | `A:PRO 390` |  | `74.20` | `0.00` | `0.00` | | `351` | `A:THR 391` |  | `49.44` | `0.00` | `0.00` | | `352` | `A:MET 392` |  | `0.00` | `0.00` | `0.00` | | `353` | `A:PHE 393` |  | `15.17` | `0.00` | `0.00` | | `354` | `A:PRO 394` |  | `19.58` | `0.00` | `0.00` | | `355` | `A:PRO 395` |  | `19.88` | `0.00` | `0.00` | | `356` | `A:LYS 396` |  | `132.18` | `0.00` | `0.00` | | `357` | `A:ASP 397` |  | `53.64` | `0.00` | `0.00` | | `358` | `A:PRO 398` |  | `101.87` | `21.56`  ||| | `0.26` | | `359` | `A:GLU 399` |  | `140.36` | `43.25`  |||| | `-0.03` | | `360` | `A:LYS 400` |  | `108.78` | `0.00` | `0.00` | | `361` | `A:LEU 401` |  | `17.22` | `0.00` | `0.00` | | `362` | `A:GLN 402` | `H` | `139.65` | `107.12`  |||||||| | `-0.72` | | `363` | `A:ALA 403` |  | `73.60` | `0.00` | `0.00` | | `364` | `A:MET 404` |  | `16.64` | `0.00` | `0.00` | | `365` | `A:HIS 405` |  | `66.40` | `6.79`  || | `0.07` | | `366` | `A:LEU 406` |  | `0.15` | `0.00` | `0.00` | | `367` | `A:GLU 407` | `HS` | `78.62` | `44.19`  |||||| | `0.44` | | `368` | `A:ARG 408` |  | `72.78` | `0.00` | `0.00` | | `369` | `A:CYS 409` |  | `0.00` | `0.00` | `0.00` | | `370` | `A:LEU 410` |  | `2.67` | `0.00` | `0.00` | | `371` | `A:ARG 411` |  | `2.32` | `0.00` | `0.00` | | `372` | `A:ILE 412` |  | `1.48` | `0.00` | `0.00` | | `373` | `A:LEU 413` |  | `0.29` | `0.00` | `0.00` | | `374` | `A:PRO 414` |  | `0.00` | `0.00` | `0.00` | | `375` | `A:HIS 415` | `HS` | `31.89` | `23.81`  |||||||| | `-0.09` | | `376` | `A:HIS 416` | `H` | `33.88` | `31.54`  |||||||||| | `0.50` | | `377` | `A:GLN 417` |  | `53.75` | `0.00` | `0.00` | | `378` | `A:ASN 418` |  | `54.37` | `0.00` | `0.00` | | `379` | `A:THR 419` |  | `9.57` | `0.00` | `0.00` | | `380` | `A:GLY 420` |  | `0.33` | `0.00` | `0.00` | | `381` | `A:GLY 421` |  | `0.98` | `0.00` | `0.00` | | `382` | `A:PHE 422` |  | `6.81` | `0.00` | `0.00` | | `383` | `A:PHE 423` |  | `0.50` | `0.00` | `0.00` | | `384` | `A:VAL 424` |  | `0.00` | `0.00` | `0.00` | | `385` | `A:ALA 425` |  | `0.00` | `0.00` | `0.00` | | `386` | `A:VAL 426` |  | `0.29` | `0.00` | `0.00` | | `387` | `A:LEU 427` |  | `0.00` | `0.00` | `0.00` | | `388` | `A:VAL 428` |  | `24.61` | `0.00` | `0.00` | | `389` | `A:LYS 429` |  | `3.21` | `0.00` | `0.00` | | `390` | `A:LYS 430` |  | `109.91` | `0.00` | `0.00` | | `391` | `A:SER 431` |  | `50.98` | `0.00` | `0.00` | | `392` | `A:SER 432` |  | `52.02` | `0.00` | `0.00` | | `393` | `A:MET 433` |  | `0.00` | `0.00` | `0.00` | | `394` | `A:PRO 434` |  | `38.77` | `0.00` | `0.00` | | `395` | `A:TRP 435` |  | `46.23` | `0.00` | `0.00` | | `396` | `A:ASN 436` |  | `40.77` | `0.00` | `0.00` | | `397` | `A:LYS 437` |  | `167.58` | `0.00` | `0.00` | | `398` | `A:ARG 438` |  | `213.87` | `0.00` | `0.00` | | `399` | `A:GLN 439` |  | `159.21` | `0.00` | `0.00` | | `400` | `A:PRO 440` |  | `116.49` | `0.00` | `0.00` | | `401` | `A:LYS 441` |  | `201.09` | `0.00` | `0.00` | | `402` | `A:LEU 442` |  | `174.27` | `0.00` | `0.00` | | `403` | `A:GLN 443` |  | `188.14` | `0.00` | `0.00` | | `404` | `A:GLY 444` |  | `123.89` | `0.00` | `0.00` | | `405` | `A:SER 496` |  | `170.35` | `0.00` | `0.00` | | `406` | `A:LYS 497` |  | `193.83` | `0.00` | `0.00` | | `407` | `A:LYS 498` |  | `201.37` | `0.00` | `0.00` | | `408` | `A:ASP 499` |  | `135.40` | `0.00` | `0.00` | | `409` | `A:GLY 500` |  | `76.23` | `0.00` | `0.00` | | `410` | `A:VAL 501` |  | `141.79` | `0.00` | `0.00` | | `411` | `A:CYS 502` |  | `109.05` | `0.00` | `0.00` | | `412` | `A:GLY 503` |  | `47.05` | `0.00` | `0.00` | | `413` | `A:PRO 504` |  | `95.18` | `0.00` | `0.00` | | `414` | `A:PRO 505` |  | `108.42` | `0.00` | `0.00` | | `415` | `A:PRO 506` |  | `123.22` | `0.00` | `0.00` | | `416` | `A:SER 507` |  | `98.94` | `0.00` | `0.00` | | `417` | `A:LYS 508` |  | `193.66` | `0.00` | `0.00` | | `418` | `A:LYS 509` |  | `190.81` | `0.00` | `0.00` | | `419` | `A:MET 510` |  | `108.98` | `0.00` | `0.00` | | `420` | `A:LYS 511` |  | `174.63` | `0.00` | `0.00` | | `421` | `A:LEU 512` |  | `94.76` | `0.00` | `0.00` | | `422` | `A:PHE 513` |  | `182.97` | `0.00` | `0.00` | | `423` | `A:GLY 514` |  | `64.31` | `0.00` | `0.00` | | `424` | `A:PHE 515` |  | `109.21` | `0.00` | `0.00` | | `425` | `A:LYS 516` |  | `195.90` | `0.00` | `0.00` | | `426` | `A:GLU 517` |  | `38.24` | `0.00` | `0.00` | | `427` | `A:ASP 518` |  | `89.42` | `0.00` | `0.00` | | `428` | `A:PRO 519` |  | `88.60` | `0.00` | `0.00` | | `429` | `A:PHE 520` |  | `28.50` | `0.00` | `0.00` | | `430` | `A:VAL 521` |  | `52.58` | `0.00` | `0.00` | | `431` | `A:PHE 522` |  | `30.33` | `0.00` | `0.00` | | `432` | `A:ILE 523` |  | `1.83` | `0.00` | `0.00` | | `433` | `A:PRO 524` |  | `76.65` | `0.00` | `0.00` | | `434` | `A:GLU 525` |  | `97.16` | `0.00` | `0.00` | | `435` | `A:ASP 526` |  | `126.90` | `0.00` | `0.00` | | `436` | `A:ASP 527` |  | `16.96` | `0.00` | `0.00` | | `437` | `A:PRO 528` |  | `112.37` | `0.00` | `0.00` | | `438` | `A:LEU 529` |  | `20.69` | `0.00` | `0.00` | | `439` | `A:PHE 530` |  | `5.23` | `0.00` | `0.00` | | `440` | `A:PRO 531` |  | `83.26` | `0.00` | `0.00` | | `441` | `A:PRO 532` |  | `53.51` | `0.00` | `0.00` | | `442` | `A:ILE 533` |  | `2.34` | `0.00` | `0.00` | | `443` | `A:GLU 534` |  | `39.63` | `0.00` | `0.00` | | `444` | `A:LYS 535` |  | `169.02` | `0.00` | `0.00` | | `445` | `A:PHE 536` |  | `16.48` | `0.00` | `0.00` | | `446` | `A:TYR 537` |  | `0.64` | `0.00` | `0.00` | | `447` | `A:ALA 538` |  | `40.81` | `0.00` | `0.00` | | `448` | `A:LEU 539` |  | `20.91` | `0.00` | `0.00` | | `449` | `A:ASP 540` |  | `44.70` | `0.00` | `0.00` | | `450` | `A:PRO 541` |  | `123.66` | `0.00` | `0.00` | | `451` | `A:SER 542` |  | `78.65` | `0.00` | `0.00` | | `452` | `A:PHE 543` |  | `8.30` | `0.00` | `0.00` | | `453` | `A:PRO 544` |  | `36.13` | `0.00` | `0.00` | | `454` | `A:ARG 545` |  | `69.13` | `0.00` | `0.00` | | `455` | `A:MET 546` |  | `65.29` | `0.00` | `0.00` | | `456` | `A:ASN 547` |  | `10.36` | `0.00` | `0.00` | | `457` | `A:LEU 548` |  | `1.01` | `0.00` | `0.00` | | `458` | `A:LEU 549` |  | `0.17` | `0.00` | `0.00` | | `459` | `A:THR 550` |  | `4.11` | `0.00` | `0.00` | | `460` | `A:ARG 551` |  | `56.20` | `0.00` | `0.00` | | `461` | `A:THR 552` |  | `47.02` | `0.00` | `0.00` | | `462` | `A:THR 553` |  | `93.95` | `0.00` | `0.00` | | `463` | `A:GLU 554` |  | `146.65` | `0.00` | `0.00` | | `464` | `A:GLY 555` |  | `69.93` | `0.00` | `0.00` | | `465` | `A:LYS 556` |  | `135.17` | `0.00` | `0.00` | | `466` | `A:LYS 557` |  | `43.06` | `0.00` | `0.00` | | `467` | `A:ARG 558` |  | `185.44` | `0.00` | `0.00` | | `468` | `A:GLN 559` |  | `72.03` | `0.00` | `0.00` | | `469` | `A:LEU 560` |  | `0.00` | `0.00` | `0.00` | | `470` | `A:TYR 561` |  | `33.51` | `0.00` | `0.00` | | `471` | `A:MET 562` |  | `0.00` | `0.00` | `0.00` | | `472` | `A:VAL 563` |  | `0.00` | `0.00` | `0.00` | | `473` | `A:SER 564` |  | `3.01` | `0.00` | `0.00` | | `474` | `A:LYS 565` |  | `146.14` | `0.00` | `0.00` | | `475` | `A:GLU 566` |  | `30.26` | `0.00` | `0.00` | | `476` | `A:LEU 567` |  | `0.00` | `0.00` | `0.00` | | `477` | `A:ARG 568` |  | `47.92` | `0.00` | `0.00` | | `478` | `A:ASN 569` |  | `27.13` | `0.00` | `0.00` | | `479` | `A:VAL 570` |  | `0.17` | `0.00` | `0.00` | | `480` | `A:LEU 571` |  | `14.98` | `0.00` | `0.00` | | `481` | `A:LEU 572` |  | `89.88` | `0.00` | `0.00` | | `482` | `A:ASN 573` |  | `28.16` | `0.00` | `0.00` | | `483` | `A:ASN 574` |  | `2.23` | `0.00` | `0.00` | | `484` | `A:SER 575` |  | `82.49` | `0.00` | `0.00` | | `485` | `A:GLU 576` |  | `156.48` | `0.00` | `0.00` | | `486` | `A:LYS 577` |  | `79.14` | `0.00` | `0.00` | | `487` | `A:MET 578` |  | `9.30` | `0.00` | `0.00` | | `488` | `A:LYS 579` |  | `40.48` | `0.00` | `0.00` | | `489` | `A:VAL 580` |  | `26.76` | `0.00` | `0.00` | | `490` | `A:ILE 581` |  | `4.80` | `0.00` | `0.00` | | `491` | `A:ASN 582` |  | `13.66` | `0.00` | `0.00` | | `492` | `A:THR 583` |  | `0.00` | `0.00` | `0.00` | | `493` | `A:GLY 584` |  | `8.20` | `0.00` | `0.00` | | `494` | `A:ILE 585` |  | `10.05` | `0.00` | `0.00` | | `495` | `A:LYS 586` |  | `40.89` | `0.00` | `0.00` | | `496` | `A:VAL 587` |  | `0.00` | `0.00` | `0.00` | | `497` | `A:TRP 588` |  | `0.77` | `0.00` | `0.00` | | `498` | `A:CYS 589` |  | `17.30` | `0.00` | `0.00` | | `499` | `A:ARG 590` |  | `57.87` | `0.00` | `0.00` | | `500` | `A:ASN 591` |  | `37.24` | `0.00` | `0.00` | | `501` | `A:ASN 592` |  | `143.32` | `0.00` | `0.00` | | `502` | `A:SER 593` |  | `51.72` | `0.00` | `0.00` | | `503` | `A:GLY 594` |  | `0.00` | `0.00` | `0.00` | | `504` | `A:GLU 595` |  | `105.31` | `0.00` | `0.00` | | `505` | `A:GLU 596` |  | `138.45` | `0.00` | `0.00` | | `506` | `A:PHE 597` |  | `50.03` | `0.00` | `0.00` | | `507` | `A:ASP 598` |  | `108.25` | `0.00` | `0.00` | | `508` | `A:CYS 599` |  | `5.36` | `0.00` | `0.00` | | `509` | `A:ALA 600` |  | `12.74` | `0.00` | `0.00` | | `510` | `A:PHE 601` |  | `7.36` | `0.00` | `0.00` | | `511` | `A:ARG 602` |  | `68.92` | `0.00` | `0.00` | | `512` | `A:LEU 603` |  | `0.12` | `0.00` | `0.00` | | `513` | `A:ALA 604` |  | `3.52` | `0.00` | `0.00` | | `514` | `A:GLN 605` |  | `14.43` | `0.00` | `0.00` | | `515` | `A:GLU 606` |  | `69.10` | `0.00` | `0.00` | | `516` | `A:GLY 607` |  | `0.49` | `0.00` | `0.00` | | `517` | `A:ILE 608` |  | `0.00` | `0.00` | `0.00` | | `518` | `A:TYR 609` |  | `72.60` | `0.00` | `0.00` | | `519` | `A:THR 610` |  | `8.09` | `0.00` | `0.00` | | `520` | `A:LEU 611` |  | `0.34` | `0.00` | `0.00` | | `521` | `A:TYR 612` |  | `49.43` | `0.00` | `0.00` | | `522` | `A:PRO 613` |  | `71.63` | `0.00` | `0.00` | | `523` | `A:PHE 614` |  | `25.82` | `0.00` | `0.00` | | `524` | `A:ILE 615` |  | `6.50` | `0.00` | `0.00` | | `525` | `A:ASN 616` |  | `96.76` | `0.00` | `0.00` | | `526` | `A:SER 617` |  | `34.49` | `0.00` | `0.00` | | `527` | `A:ARG 618` |  | `23.43` | `0.00` | `0.00` | | `528` | `A:ILE 619` |  | `54.30` | `0.00` | `0.00` | | `529` | `A:ILE 620` |  | `1.00` | `0.00` | `0.00` | | `530` | `A:THR 621` |  | `66.03` | `0.00` | `0.00` | | `531` | `A:VAL 622` |  | `6.26` | `0.00` | `0.00` | | `532` | `A:SER 623` |  | `41.39` | `0.00` | `0.00` | | `533` | `A:MET 624` |  | `27.79` | `0.00` | `0.00` | | `534` | `A:GLU 625` |  | `99.27` | `0.00` | `0.00` | | `535` | `A:ASP 626` |  | `2.16` | `0.00` | `0.00` | | `536` | `A:VAL 627` |  | `0.74` | `0.00` | `0.00` | | `537` | `A:LYS 628` |  | `55.02` | `0.00` | `0.00` | | `538` | `A:ILE 629` |  | `42.81` | `0.00` | `0.00` | | `539` | `A:LEU 630` |  | `1.15` | `0.00` | `0.00` | | `540` | `A:LEU 631` |  | `0.28` | `0.00` | `0.00` | | `541` | `A:THR 632` |  | `36.86` | `0.00` | `0.00` | | `542` | `A:GLN 633` |  | `70.02` | `0.00` | `0.00` | | `543` | `A:GLU 634` |  | `100.91` | `0.00` | `0.00` | | `544` | `A:ASN 635` |  | `77.25` | `0.00` | `0.00` | | `545` | `A:PRO 636` |  | `3.18` | `0.00` | `0.00` | | `546` | `A:PHE 637` |  | `114.19` | `0.00` | `0.00` | | `547` | `A:PHE 638` |  | `12.34` | `0.00` | `0.00` | | `548` | `A:ARG 639` |  | `192.97` | `0.00` | `0.00` | | `549` | `A:LYS 640` |  | `118.38` | `0.00` | `0.00` | | `550` | `A:LEU 641` |  | `11.81` | `0.00` | `0.00` | | `551` | `A:SER 642` |  | `39.73` | `0.00` | `0.00` | | `552` | `A:SER 643` |  | `85.97` | `0.00` | `0.00` | | `553` | `A:GLU 644` |  | `119.06` | `0.00` | `0.00` | | `554` | `A:THR 645` |  | `0.49` | `0.00` | `0.00` | | `555` | `A:TYR 646` |  | `67.28` | `0.00` | `0.00` | | `556` | `A:SER 647` |  | `60.73` | `0.00` | `0.00` | | `557` | `A:GLN 648` |  | `63.99` | `0.00` | `0.00` | | `558` | `A:ALA 649` |  | `0.00` | `0.00` | `0.00` | | `559` | `A:LYS 650` |  | `115.10` | `0.00` | `0.00` | | `560` | `A:ASP 651` |  | `117.72` | `0.00` | `0.00` | | `561` | `A:LEU 652` |  | `40.23` | `0.00` | `0.00` | | `562` | `A:ALA 653` |  | `61.49` | `0.00` | `0.00` | | `563` | `A:LYS 654` |  | `85.08` | `0.00` | `0.00` | | `564` | `A:GLY 655` |  | `0.00` | `0.00` | `0.00` | | `565` | `A:SER 656` |  | `1.64` | `0.00` | `0.00` | | `566` | `A:ILE 657` |  | `0.33` | `0.00` | `0.00` | | `567` | `A:VAL 658` |  | `0.34` | `0.00` | `0.00` | | `568` | `A:LEU 659` |  | `0.48` | `0.00` | `0.00` | | `569` | `A:LYS 660` |  | `34.72` | `0.00` | `0.00` | | `570` | `A:TYR 661` |  | `13.00` | `0.00` | `0.00` | | `571` | `A:GLU 662` |  | `93.53` | `0.00` | `0.00` | | `572` | `A:PRO 663` |  | `38.75` | `0.00` | `0.00` | | `573` | `A:ASP 664` |  | `72.80` | `0.00` | `0.00` | | `574` | `A:SER 665` |  | `109.32` | `0.00` | `0.00` | | `575` | `A:ALA 666` |  | `90.69` | `0.00` | `0.00` | | `576` | `A:ASN 667` |  | `67.33` | `0.00` | `0.00` | | `577` | `A:PRO 668` |  | `106.74` | `0.00` | `0.00` | | `578` | `A:ASP 669` |  | `142.55` | `0.00` | `0.00` | | `579` | `A:ALA 670` |  | `28.41` | `0.00` | `0.00` | | `580` | `A:LEU 671` |  | `15.89` | `0.00` | `0.00` | | `581` | `A:GLN 672` |  | `87.28` | `0.00` | `0.00` | | `582` | `A:CYS 673` |  | `3.64` | `0.00` | `0.00` | | `583` | `A:PRO 674` |  | `65.80` | `0.00` | `0.00` | | `584` | `A:ILE 675` |  | `0.75` | `0.00` | `0.00` | | `585` | `A:VAL 676` |  | `5.35` | `0.00` | `0.00` | | `586` | `A:LEU 677` |  | `0.84` | `0.00` | `0.00` | | `587` | `A:CYS 678` |  | `0.33` | `0.00` | `0.00` | | `588` | `A:GLY 679` |  | `0.00` | `0.00` | `0.00` | | `589` | `A:TRP 680` |  | `44.03` | `0.00` | `0.00` | | `590` | `A:ARG 681` |  | `22.43` | `0.00` | `0.00` | | `591` | `A:GLY 682` |  | `14.80` | `0.00` | `0.00` | | `592` | `A:LYS 683` |  | `166.69` | `0.00` | `0.00` | | `593` | `A:ALA 684` |  | `40.13` | `0.00` | `0.00` | | `594` | `A:SER 685` |  | `25.35` | `0.00` | `0.00` | | `595` | `A:ILE 686` |  | `0.31` | `0.00` | `0.00` | | `596` | `A:ARG 687` |  | `101.06` | `0.00` | `0.00` | | `597` | `A:THR 688` |  | `8.72` | `0.00` | `0.00` | | `598` | `A:PHE 689` |  | `49.22` | `0.00` | `0.00` | | `599` | `A:VAL 690` |  | `7.97` | `0.00` | `0.00` | | `600` | `A:PRO 691` |  | `69.80` | `0.00` | `0.00` | | `601` | `A:LYS 692` |  | `144.25` | `0.00` | `0.00` | | `602` | `A:ASN 693` |  | `33.61` | `0.00` | `0.00` | | `603` | `A:GLU 694` |  | `36.66` | `0.00` | `0.00` | | `604` | `A:ARG 695` |  | `59.34` | `0.00` | `0.00` | | `605` | `A:LEU 696` |  | `23.73` | `0.00` | `0.00` | | `606` | `A:HIS 697` |  | `4.78` | `0.00` | `0.00` | | `607` | `A:TYR 698` |  | `3.14` | `0.00` | `0.00` | | `608` | `A:LEU 699` |  | `0.17` | `0.00` | `0.00` | | `609` | `A:ARG 700` |  | `43.69` | `0.00` | `0.00` | | `610` | `A:MET 701` |  | `24.34` | `0.00` | `0.00` | | `611` | `A:MET 702` |  | `20.78` | `0.00` | `0.00` | | `612` | `A:GLY 703` |  | `65.10` | `0.00` | `0.00` | | `613` | `A:LEU 704` |  | `17.85` | `0.00` | `0.00` | | `614` | `A:GLU 705` |  | `50.40` | `0.00` | `0.00` | | `615` | `A:VAL 706` |  | `23.54` | `0.00` | `0.00` | | `616` | `A:LEU 707` |  | `95.17` | `0.00` | `0.00` | | `617` | `A:GLY 708` |  | `45.68` | `0.00` | `0.00` | | `618` | `A:GLU 709` |  | `105.89` | `0.00` | `0.00` | | `619` | `A:LYS 710` |  | `196.95` | `0.00` | `0.00` | | `620` | `A:LYS 711` |  | `193.76` | `0.00` | `0.00` | | `621` | `A:LYS 712` |  | `189.88` | `0.00` | `0.00` | | `622` | `A:GLU 713` |  | `161.74` | `0.00` | `0.00` | | `623` | `A:GLY 714` |  | `79.28` | `0.00` | `0.00` | | `624` | `A:VAL 715` |  | `106.68` | `0.00` | `0.00` | | `625` | `A:ILE 716` |  | `33.27` | `0.00` | `0.00` | | `626` | `A:LEU 717` |  | `151.18` | `0.00` | `0.00` | | `627` | `A:THR 718` |  | `39.85` | `0.00` | `0.00` | | `628` | `A:ASN 719` |  | `87.58` | `0.00` | `0.00` | | `629` | `A:GLU 720` |  | `142.74` | `0.00` | `0.00` | | `630` | `A:SER 721` |  | `102.13` | `0.00` | `0.00` | | `631` | `A:ALA 722` |  | `94.54` | `0.00` | `0.00` | | `632` | `A:ALA 723` |  | `100.87` | `0.00` | `0.00` | | `633` | `A:SER 724` |  | `158.58` | `0.00` | `0.00` | |

---

|  |  |  |
| --- | --- | --- |
| <<   <   >   >> | | |

PDBe PISA v1.52 [20/10/2014]

### EMBL-EBI

### Services

### Research

### Training

### Industry

### About us

EMBL-EBI, Wellcome Trust Genome Campus, Hinxton, Cambridgeshire, CB10 1SD, UK     +44 (0)1223 49 44 44

Copyright © EMBL-EBI 2012 | EBI is an Outstation of the European Molecular Biology Laboratory | Privacy | Cookies | Terms of use
