# Supplementary material for: NONO regulates m5C modification and alternative splicing of PTEN mRNAs to drive gastric cancer progression
Source: J Exp Clin Cancer Res. 2025 Mar 4;44:81. doi: 10.1186/s13046-024-03260-z (PMC11877715; doi:10.1186/s13046-024-03260-z)
Supplement: Supplementary file 6 — Supplementary Material 6 [file 13046_2024_3260_MOESM6_ESM.docx]

**Supplementary** **figure Legends**

**
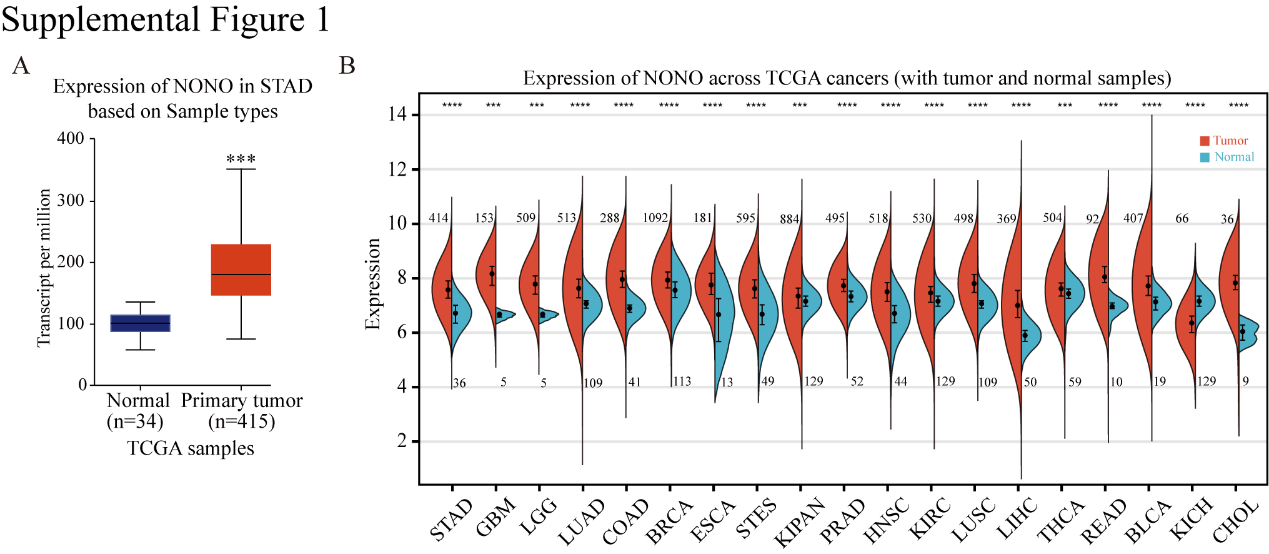
**

**Fig. S1 Aberrant expression of NONO and malignant phenotypes in GC cancers.**

**A.** The level of NONO mRNA was significantly increased from normal stomach tissues to gastric cancer tissues in TCGA dataset and p values were indicated. **B.** Expression of NONO across 19 different cancers in TCGA database.


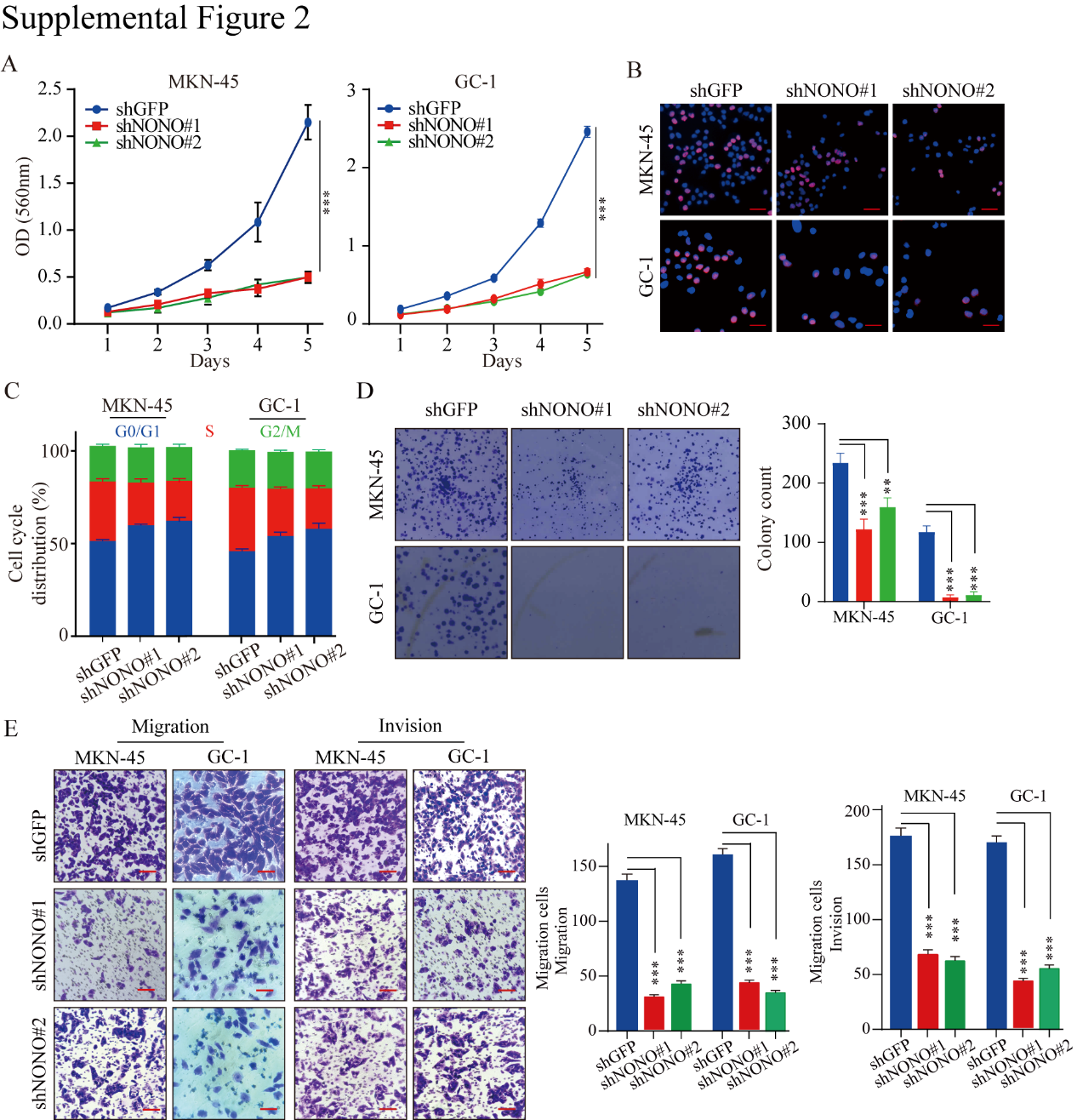


**Fig. S2 NONO promotes cell proliferation and metastasis of GC cells.**

**A B.** The proliferation of cells under knockdown NONO are determined via MTT (A) and EDU (B) assays, Scale bar = 100 μm. **C.** Flow cytometry analysis of propidium iodide (PI) staining for the detection of the cell cycle in MKN-45 and GC-1 transfected with shGFP, shNONO#1, and shNONO#2 (n = 3). **D.** Two-dimensional colony formation for the detection of cell proliferation in MKN-45 and GC-1 transfected with shGFP, shNONO#1, and shNONO#2. **E.** Transwell assay to evaluate invasion of NONO-knockdown NONO in GC cell lines (n = 3). Scale bar = 100 μm. Data shown as means ± SD. The *p* values were calculated using an unpaired two-tailed Student’s t test; ∗p < 0.05, ∗∗p < 0.01, ∗∗∗p <0.001.


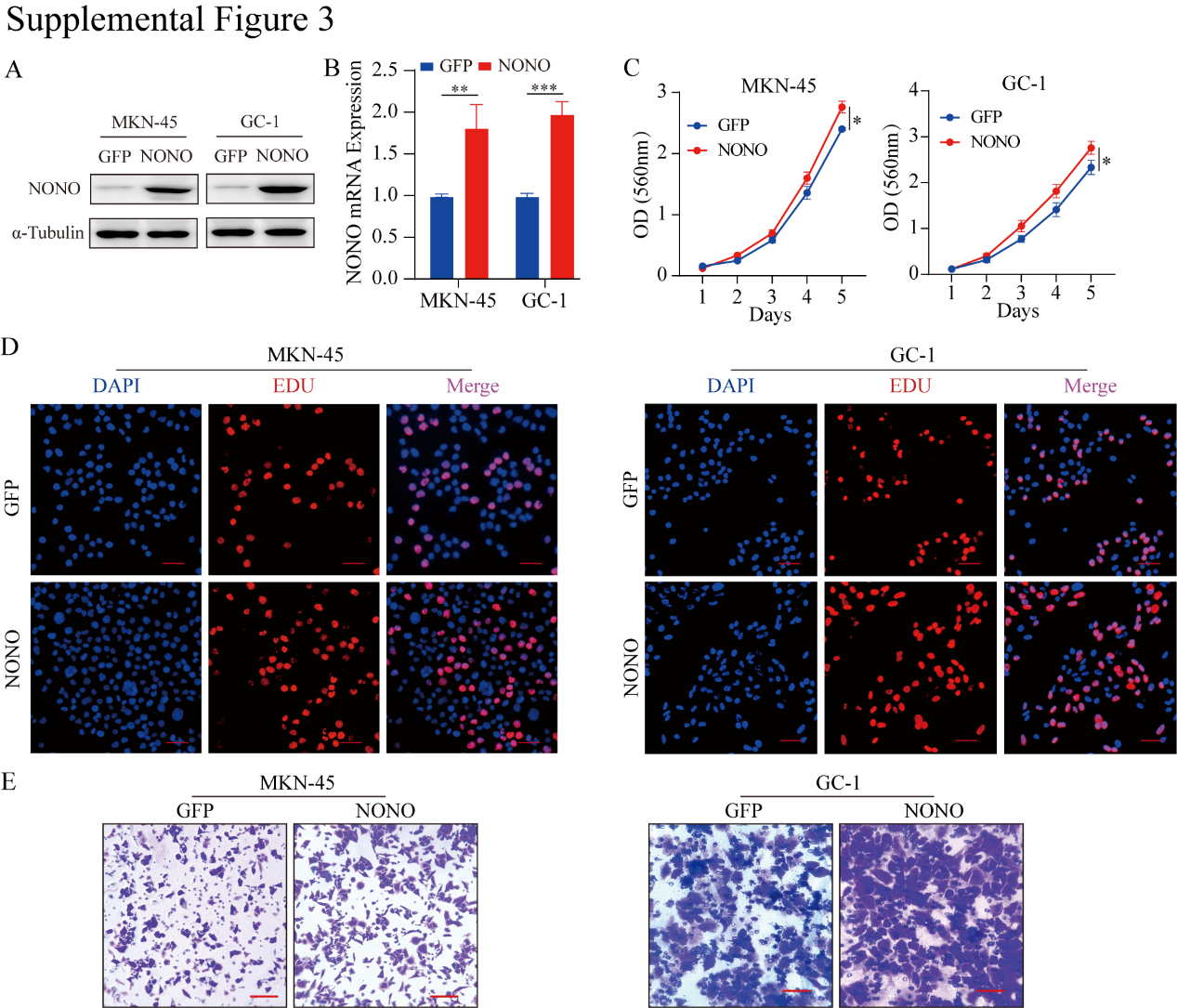


**Fig. S3 NONO promotes cell proliferation and metastasis of GC cells.**

**A B.** Western Blot and qRT-PCR assay were performed to prove the overexpression of NONO. **C D.** The proliferation of cells under overexpression NONO are determined via MTT and EDU assays (n = 3), Scale bar = 100 μm. **E.** Transwell assay to evaluate invasion of NONO-knockdown or overexpression NONO in GC cell lines (n = 3). Scale bar = 100 μm. Data shown as means ± SD. The *p* values were calculated using an unpaired two-tailed Student’s t test; ∗p < 0.05, ∗∗p < 0.01, ∗∗∗p <0.001.

**
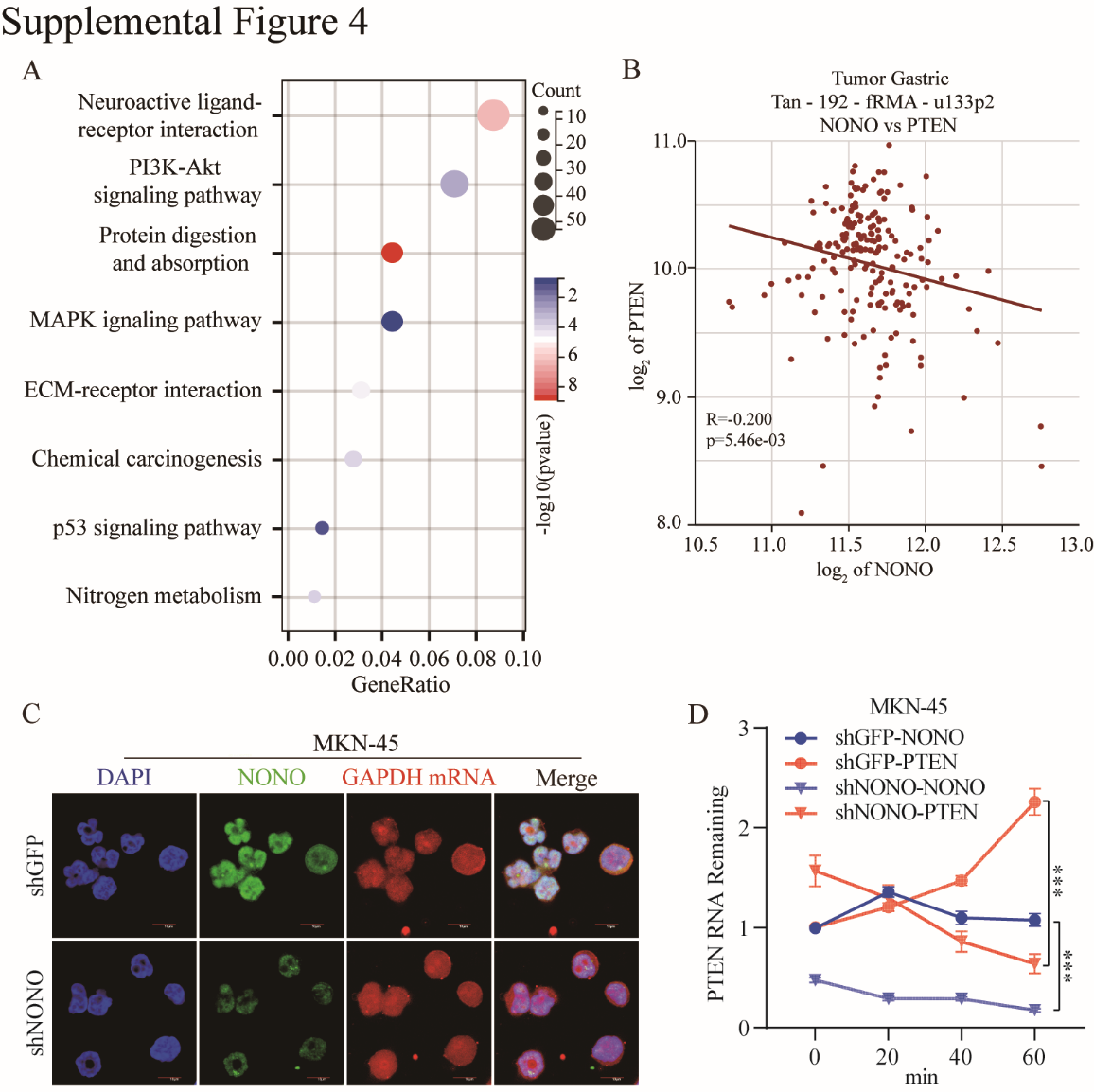
**

**Fig. S4 Downstream pathway regulated by NONO in GC.**

**A.** The bubble plots showing KEGG pathway enrichment data for genes which were up-regulated. Kyoto Encyclopedia of Genes and Genomes (KEGG) pathway enrichment analysis of NONO-regulated module genes.

**B.** Pearson correlations between the expression of PTEN and NONO in Tumor Gastric Tan-192-fRMA-u133p2. **C.** Representative images of RNA FISH for GAPDH mRNA (red) and immunofluorescence for NONO (green) in MKN-45. Scale bar = 10 μm. **D.** Reduced PTEN FL-mRNA half-life by knockdown NONO in MKN-45 cells. Data are mean ± s.d. (n = 3 independent experiments).


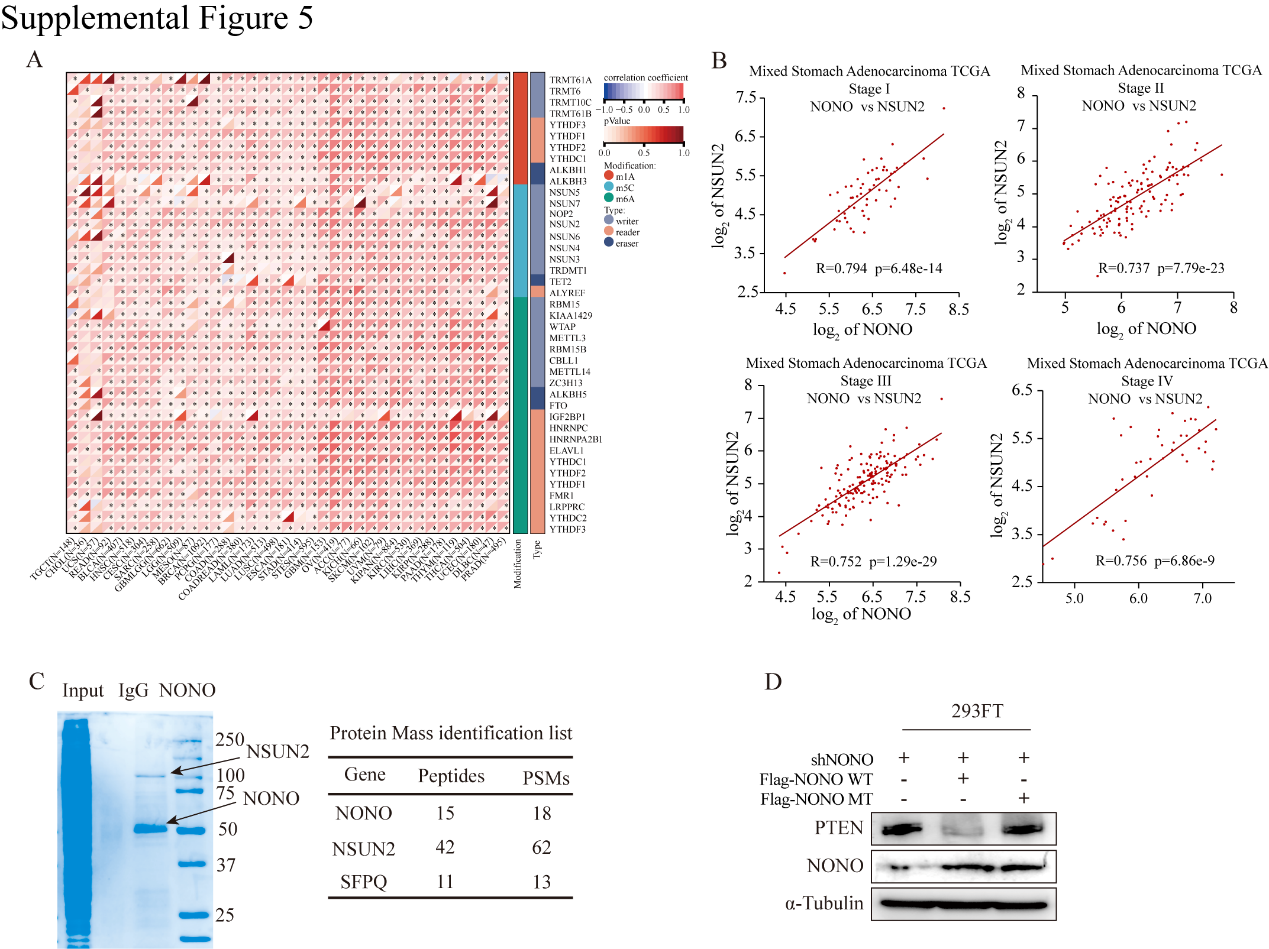


**Fig. S5. NONO Interacts with NSUN2 in GC.**

**A.** Pearson correlation between transcription of NONO and genes related to three RNA-modification types in TCGA-STAD. **B.** Pearson correlations between the expression of NONO and NSUN2 about different stage in TCGA-STAD. **C.** NONO protein were immunoprecipitated from MKN-45 cells, separated by SDS–PAGE, and subjected to Commassie Blue staining. **D.** Effects of NONO wild type (WT) and mutants (MT) on PTEN expression.

**
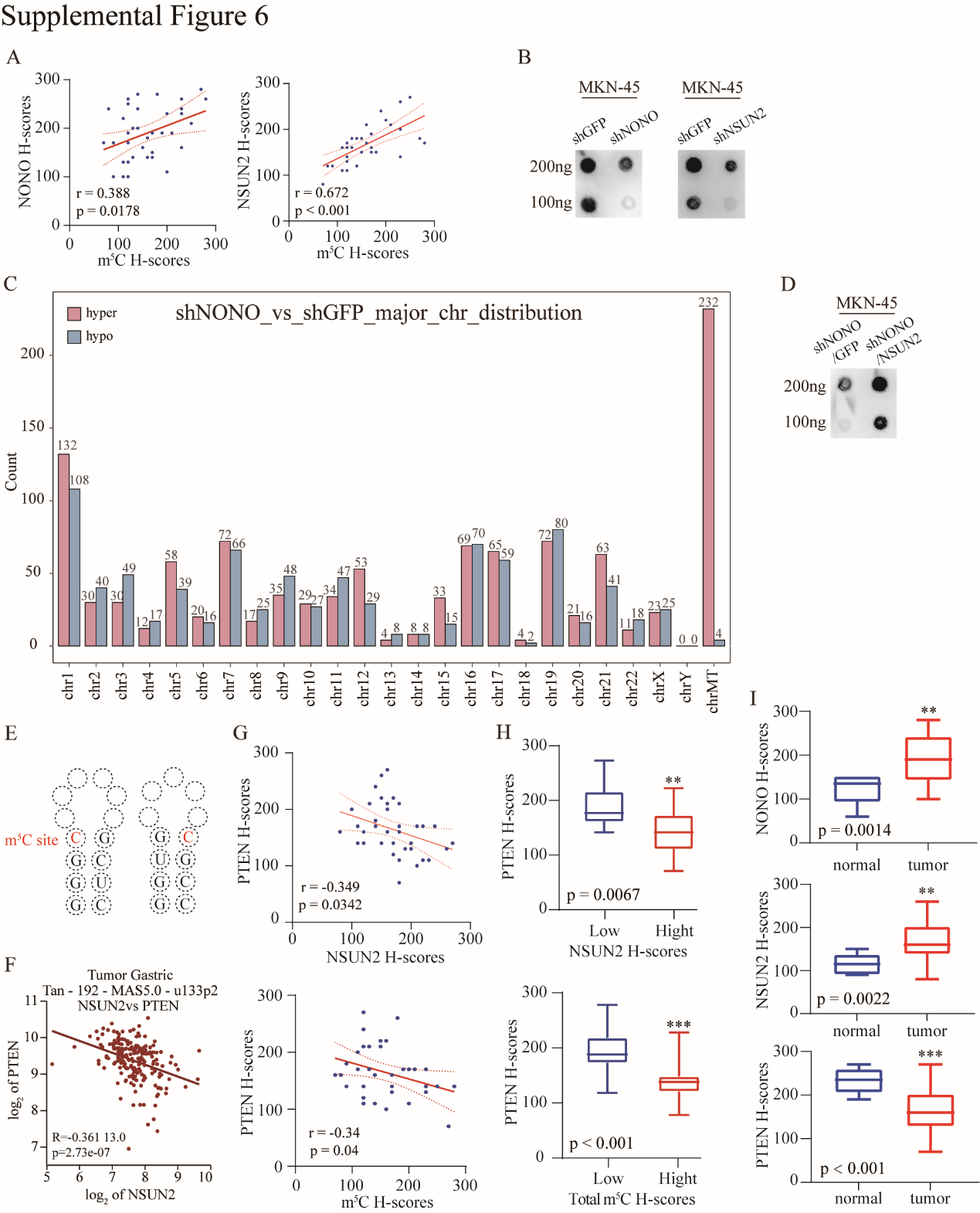
**

**Fig. S6 The m^5^C pattern at PTEN mRNAs is affected by NONO and NSUN2.**

**A.** Correlation of m^5^C methylation with proteins expression levels of NONO (top) and NSUN2 (down) in 40 gastric tissue samples. The regression lines and 95% confidence intervals are shown. The Spearman’s correlation coefficients (R) and *p* values were calculated using the Spearman correlation. **B.** m^5^C dot blot assay of knockdown of NONO or NSUN2 in MKN-45 cells. **C.** Distribution of differential m^5^C methylation sites on chromosomes. **D.** m^5^C dot blot assay of overexpress NSUN2 after knockdown of NONO in MKN-45 cells. **E.** The predicted secondary structure of changed m^5^C sites (m^5^C site: chr10: 87947629/ 87949987). The m^5^C site is indicated by the red font. **F.** Pearson correlations between the expression of PTEN and NSUN2 in Tumor Gastric Tan-192-fRMA-u133p2. R: Pearson’s correlation coefficient. *p* value was based on two-sided Pearson’s correlation test. **G.** Correlation of m^5^C methylation with proteins expression levels of NONO (top) and NSUN2 (down) in 40 gastric tissue samples. The regression lines and 95% confidence intervals are shown. The Spearman’s correlation coefficients (R) and *p* values were calculated using the Spearman correlation. **H.** Distribution of the protein expression of PTEN in high expression of NSUN2 or m^5^C methylation tumors compared with matched low expression samples. The horizontal bars in the boxplots represent the median and the box edges represent the first and third interquartile ranges. **I.** The differential expression of NONO/NSUN2/PTEN between tumor and adjacent normal tissue. The *p* values were calculated using a two-sided unpaired Student’s t-test.


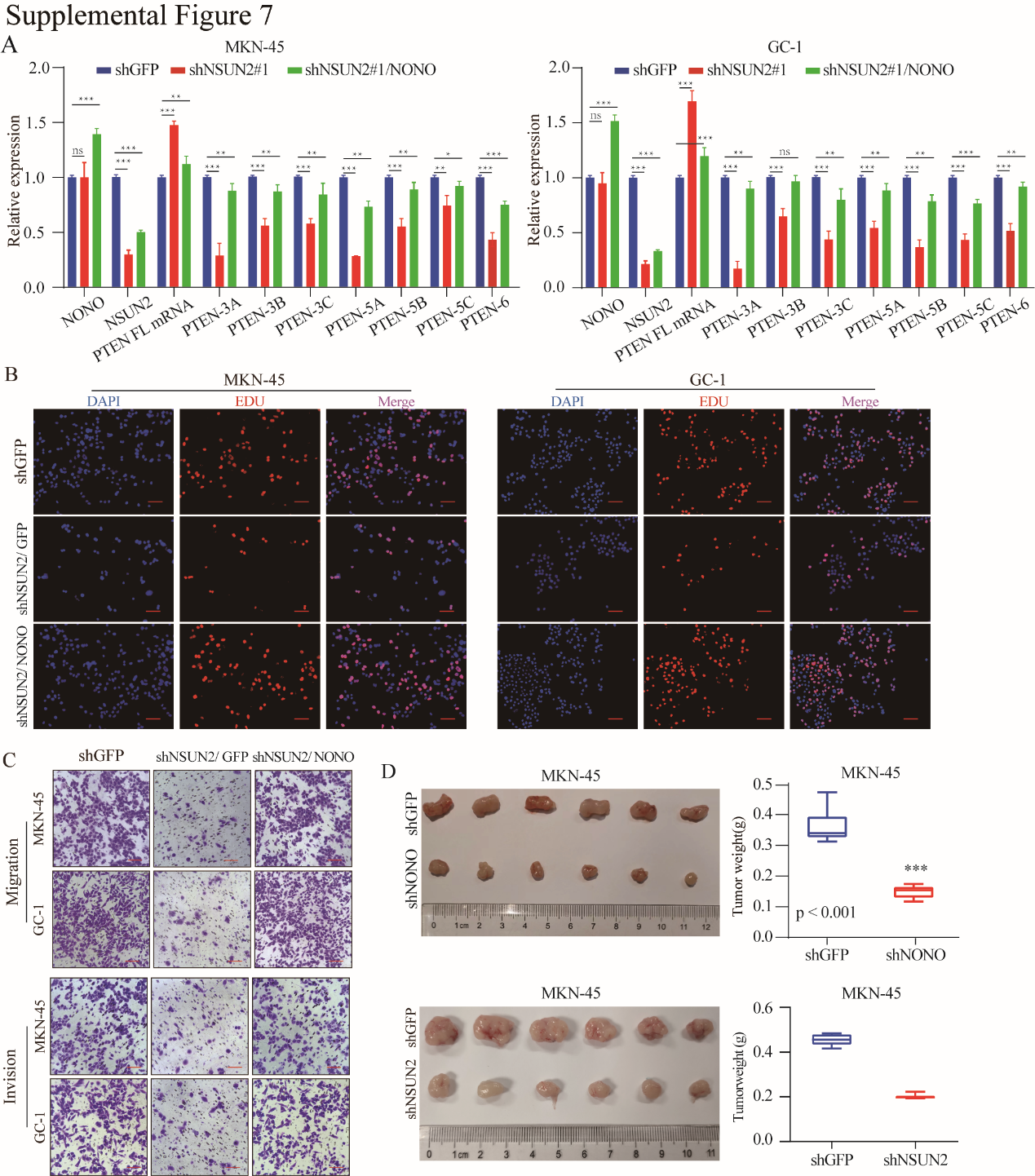


**Fig. S7 PTEN Alternative splicing dependent on NONO and NSUN2.**

**A.** qRT-PCR analysis of PTEN splicing mRNAs expression under overexpression NONO after NSUN2 knockdown. **B.** Representative images of EDU assays with MKN-45 and GC-1 cells under overexpression NONO after NSUN2 knockdown. Scale bar = 100 μm. **C.** Representative images of Transwell assays with MKN-45 and GC-1 cells under overexpression NONO after NSUN2 knockdown. Scale bar = 100. **D.** Xenograft assays were performed in MKN-45 cells under NONO or NSUN2 knockdown (n=6). The weight of tumors was analyzed and P-values were indicated. Data shown as means ± SD. The *p* values were calculated using an unpaired two-tailed Student’s t test; ∗p < 0.05, ∗∗p < 0.01, ∗∗∗p <0.001.


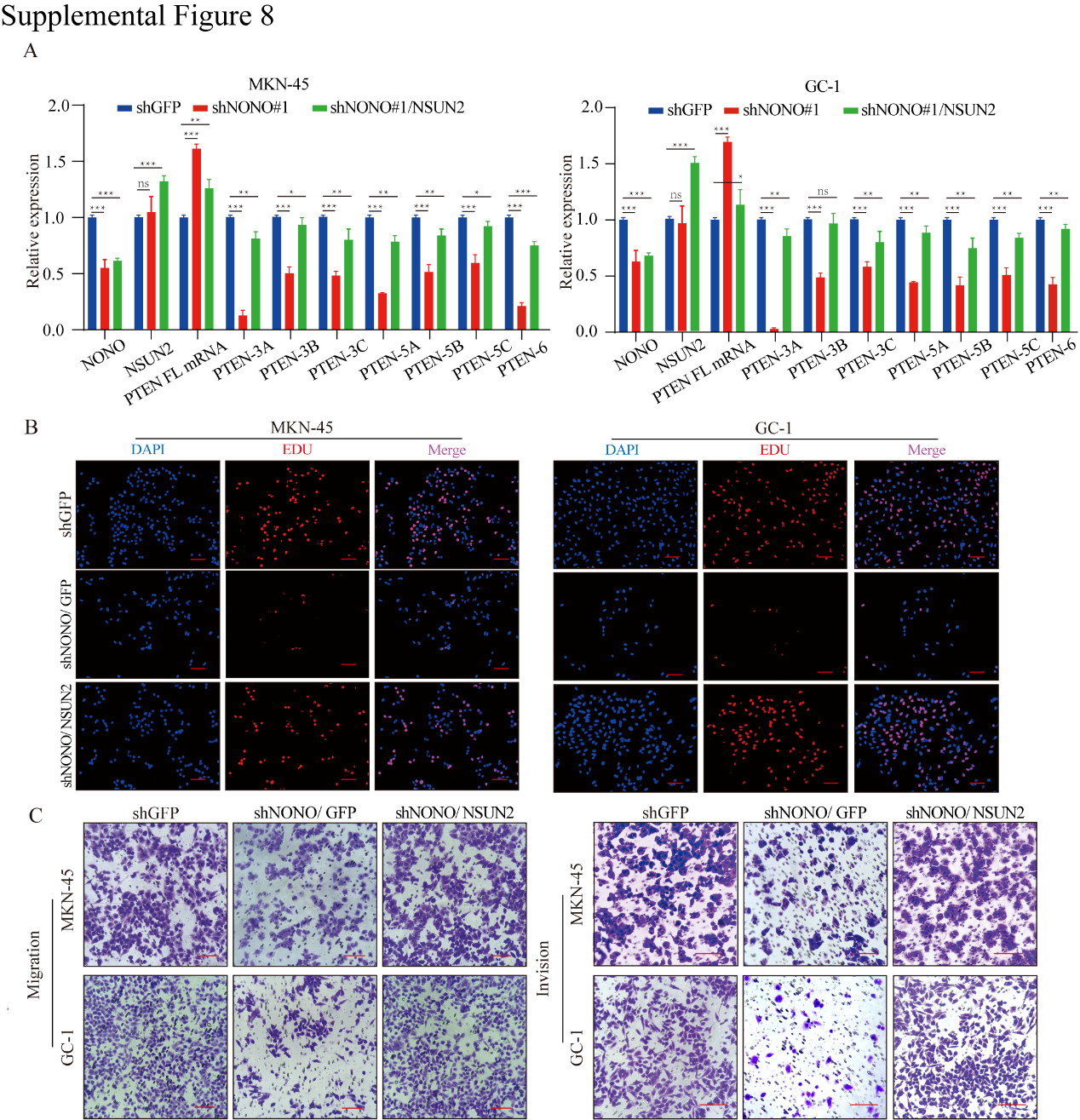


**Fig. S8 NONO promotes GC pathogenesis in an NSUN2-dependent mechanism.**

**A.** qRT-PCR analysis of PTEN splicing mRNAs expression under overexpression NSUN2 after NONO knockdown. **B.** Representative images of EDU assays with MKN-45 and GC-1 cells under overexpression NSUN2 after NONO knockdown. Scale bar=100 μm. **C.** Representative images of Transwell assays with MKN-45 and GC-1 cells under overexpression NSUN2 after NONO knockdown. Scale bar = 100 μm. Data shown as means ± SD. The *p* values were calculated using an unpaired two-tailed Student’s t test; ∗p < 0.05, ∗∗p < 0.01, ∗∗∗p <0.001.

**Supplementary Table**

Table 1. Clinicopathological features and NONO in GC from TCGA.

| Variables | All patients | Low | High | P value |
| --- | --- | --- | --- | --- |
| Gender |  |  |  |  |
| Female | 144 | 85 | 59 | 0.7237 |
| Male | 263 | 147 | 116 |  |
| Age |  |  |  |  |
| <65 | 165 | 50 | 115 | 0.3311 |
| ≥65 | 242 | 182 | 60 |  |
| Grade |  |  |  |  |
| 1 +2 | 160 | 106 | 54 | 0.0035 |
| 3 | 238 | 121 | 117 |  |
| Stage |  |  |  |  |
| I +II | 185 | 102 | 83 | 0.0165 |
| III +IV | 198 | 120 | 78 |  |

Table 2. Clinicopathological features and NONO in GC tissue microarray

| Variables | All patients | Low | High | P value |
| --- | --- | --- | --- | --- |
| Gender |  |  |  |  |
| Female | 5 | 3 | 2 | 0.5575 |
| Male | 35 | 19 | 16 |  |
| Age |  |  |  |  |
| <65 | 31 | 16 | 15 | 0.3939 |
| ≥65 | 9 | 6 | 3 |  |
| Grade |  |  |  |  |
| 1 +2 | 17 | 12 | 5 | 0.0367 |
| 3 | 23 | 10 | 13 |  |
| Stage |  |  |  |  |
| I +II | 16 | 12 | 4 | 0.0253 |
| III +IV | 24 | 10 | 14 |  |

Supplementary Table 3 Antibodies

| Antibodies | Source | Dilution |
| --- | --- | --- |
| Anti-NONO | Abcam | 1:1000 |
| Anti-CDK4 | Abcam | 1:1000 |
| Anti-cyclinD1 | Abcam | 1:1000 |
| Anti- CDK6 | Abcam | 1:1000 |
| Anti-MMP2 | Abcam | 1:1000 |
| Anti-MMP7 | Abcam | 1:1000 |
| Anti-Slug | Abcam | 1:1000 |
| Anti-PI3K | Proteintech | 1:1000 |
| Anti-AKT | Abcam | 1:1000 |
| Anti- p-AKT | Abcam | 1:1000 |
| Anti- PTEN | Abcam | 1:1000 |
| Anti- p53 | Abcam | 1:1000 |
| Anti- NSUN2 | Proteintech | 1:1000 |
| Anti-HA tag | Abcam | 1:1000 |
| Anti-Flag tag | Abcam | 1:1000 |
| Anti-α-Tubuli­n | Abcam | 1:1000 |
| anti-Mouse IgG-HRP | Abcam | 1:10000 |
| anti-Rabbit IgG-HRP | Abcam | 1:10000 |
| anti-Rabbit IgG, Alexa Fluor 594 | Thermo Fisher | 1:1000 |
| anti-mouse IgG, Alexa Fluor 488 | Thermo Fisher | 1:1000 |
| anti-Rabbit IgG, Alexa Fluor 488 | Thermo Fisher | 1:1000 |
| anti-mouse IgG, Alexa Fluor 594 | Thermo Fisher | 1:1000 |
|  |  |  |
|  |  |  |

Supplementary Table 4 qRT-PCR primers

| Primers | Sequences |
| --- | --- |
| NONO | F: 5’- GGCAGGCGAAGTCTTCATTCA-3’  R: 5’- TGGCAATCTCCGCTAGGGT-3’ |
| PTEN pre-mRNA | F: 5’- TGATGTATAAACCGTGAG-3’  R: 5’- TGCAGGAAATCCCATAGC-3’ |
| PTEN mRNA | F: 5’- CAGCCATCATCAAAGAGATCG-3’  R: 5’- TTGTTCCTGTATACGCCTTCAA-3’ |
| PTEN-3A | F: 5’- CAGCCATCATCAAAGAGATCG-3’  R: 5’- CTTTCAGCACAATTAACTTCTCT-3’ |
| PTEN-3B | F: 5’- CAGCCATCATCAAAGAGATCG-3’  R: 5’- CTGTGTGACCTTGTTCAACTCA-3’ |
| PTEN-3C | F: 5’- CAGCCATCATCAAAGAGATCG-3’  R: 5’- GCAGTACCCTGGTAACTCCAA-3’ |
| PTEN-5A | F: 5’- TCTTTGTGCTGAAAGACATT-3’  R: 5’- GGAGCCTTCTCTTGGATTTAA-3’ |
| PTEN-5B | F: 5’- TCTTTGTGCTGAAAGACATT-3’  R: 5’- CGCCTCGGCCTCCCAAAGT-3’ |
| PTEN-5C | F: 5’- TCTTTGTGCTGAAAGACATT-3’  R: 5’- GGCCTCTACAAGGTCAGGATCAT-3’ |
| PTEN-6 | F: 5’- AGGACCAGAGACAAAAAGATC-3’  R: 5’- CTGGTCCTGGTATGAAGAAT-3’ |
| GAPDH | F: 5’-GGAGCGAGATCCCTCCAAAAT-3’  R: 5’-GGCTGTTGTCATACTTCTCATGG-3’ |
|  |  |

Supplementary Table 5 shRNA and plasmids

| Primers/gene | Sequences/Gene ID | Vector |
| --- | --- | --- |
| shGFP | 5’-GCAAGCTGACCCTGAAGTTCA-3’ | pLKO.1 - Puro |
| shNONO#1 | 5’- CAGGCGAAGTCTTCATTCATA -3’ | pLKO.1 - Puro |
| shNONO#2 | 5’- GCAGGCGAAGTCTTCATTCAT -3’ | pLKO.1 - Puro |
| shNSUN2#1 | 5’- GAACAAGCTGTTCGAGCACTA-3’ | pLKO.1 - Puro |
| shNSUN2#2 | 5’- GCACACAAATTTAAGTCGAAA-3 | pLKO.1 - Puro |
| NONO | Gene ID: 4841 | pCDH-GFP+Puro-Flag-N--Flag-C |
| NONO ΔRRM1 | NONO (1-228, 439-1416aa) | pCDH-GFP+Puro-Flag-N--Flag-C |
| NONO ΔRRM2 | NONO (1-438, 679-1416aa) | pCDH-GFP+Puro-Flag-N--Flag-C |
| NONO ΔRRM1/2 | NONO (1-228, 679-1416aa) | pCDH-GFP+Puro-Flag-N--Flag-C |
|  |  |  |
| NONO ΔNOPS | NONO (1-678, 826-1416aa) | pCDH-GFP+Puro-Flag-N--Flag-C |
|  |  |  |
| NONO ΔCC | NONO (1-825, 1117-1416aa) | pCDH-GFP+Puro-Flag-N--Flag-C |
| NSUN2 | Gene ID: 54888 | pCDH-GFP+Puro-HA-N--HA-C |

Supplementary Table 6 Probes used for RNA FISH.

| Target transcript | Probe sequence (5’-3’) |
| --- | --- |
| PTEN- probe1 | GATGGAAATGGCTCTGGACTTGGCGGTAG |
| PTEN- probe2 | CGCCGCCGTGTTGGAGGCAGTAGAAGGG |
| U6 probe | TTTGCGTGTCATCTTCG |
| 18s rRNA probe | CTGCCTTCCTTGGATGTGGGTAGCCGTTTC |

Supplementary Table 7 Primers used for RIP-qRT-PCR analysis.

| Primers | Sequences |
| --- | --- |
| PTEN mRNA | F: 5’- CAGCCATCATCAAAGAGATCG-3’  R: 5’- TTGTTCCTGTATACGCCTTCAA-3’ |
| PTEN pre-mRNA | F: 5’- GGATTCGACTTAGACTTGACCTATATT-3’  R: 5’- CCTGTATACGCCTTCAAGTCTTT-3’ |
| PTEN spliced RNA | F: 5’- CTTTGTGCTGAAAGACATTATGACA-3’  R: 5’- GTTCTAGCTGTGGTGGGTTATG-3’ |
| GAPDH | F: 5’-GGAGCGAGATCCCTCCAAAAT-3’  R: 5’-GGCTGTTGTCATACTTCTCATGG-3’ |

Supplementary Table 8 Primers used for T7 Promoter-PTEN mRNA.

| Primers | Sequences |
| --- | --- |
| T7-PTEN-F | 5’-TAATACGACTCACTATAGGGATGACAGCCATCATCAAAGAG-3’ |
| T7-PTEN-R | 5’-TCAGACTTTTGTAATTTGTGTATGCTGATCTTCATC-3’ |


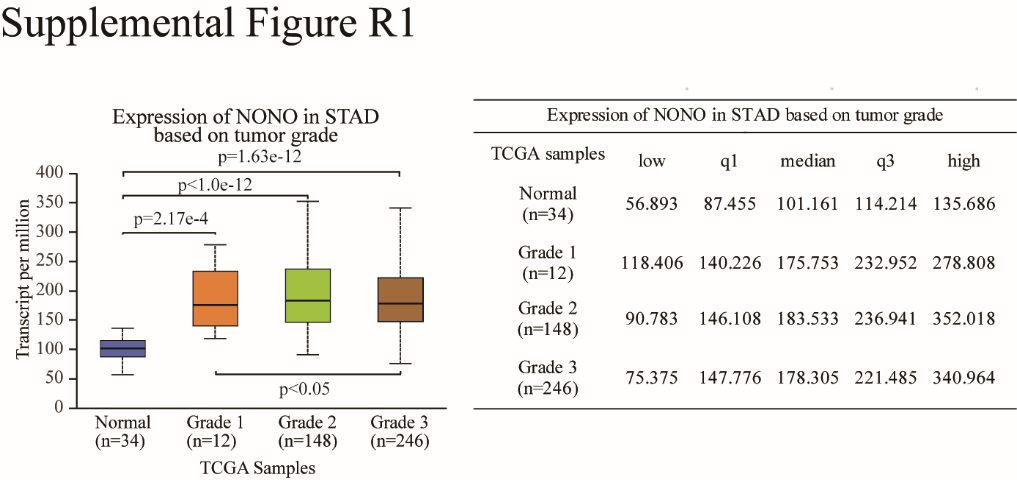


Supplemental Figure R1 Expression of NONO in STAD based on tumor grade.


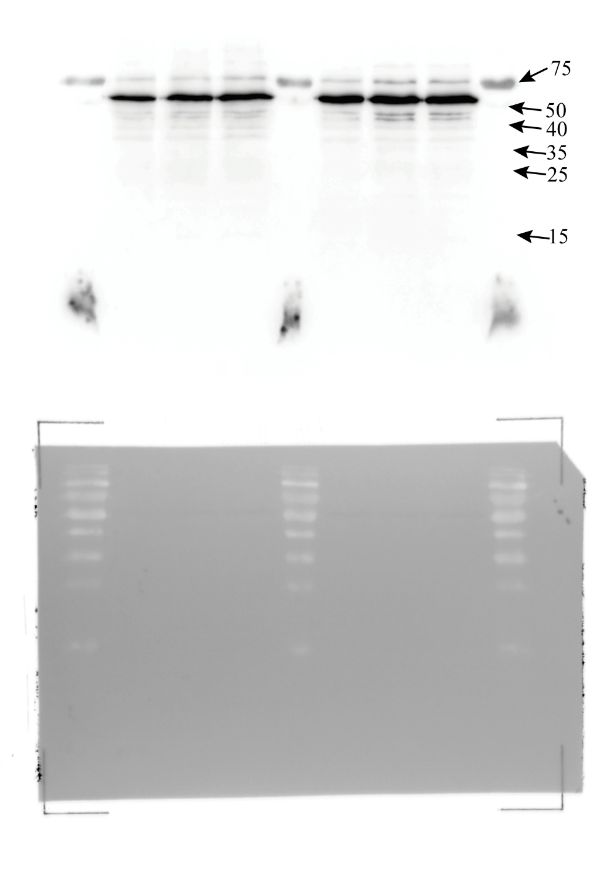


Supplemental Figure R2: FL-PTEN protein detected by western blot.


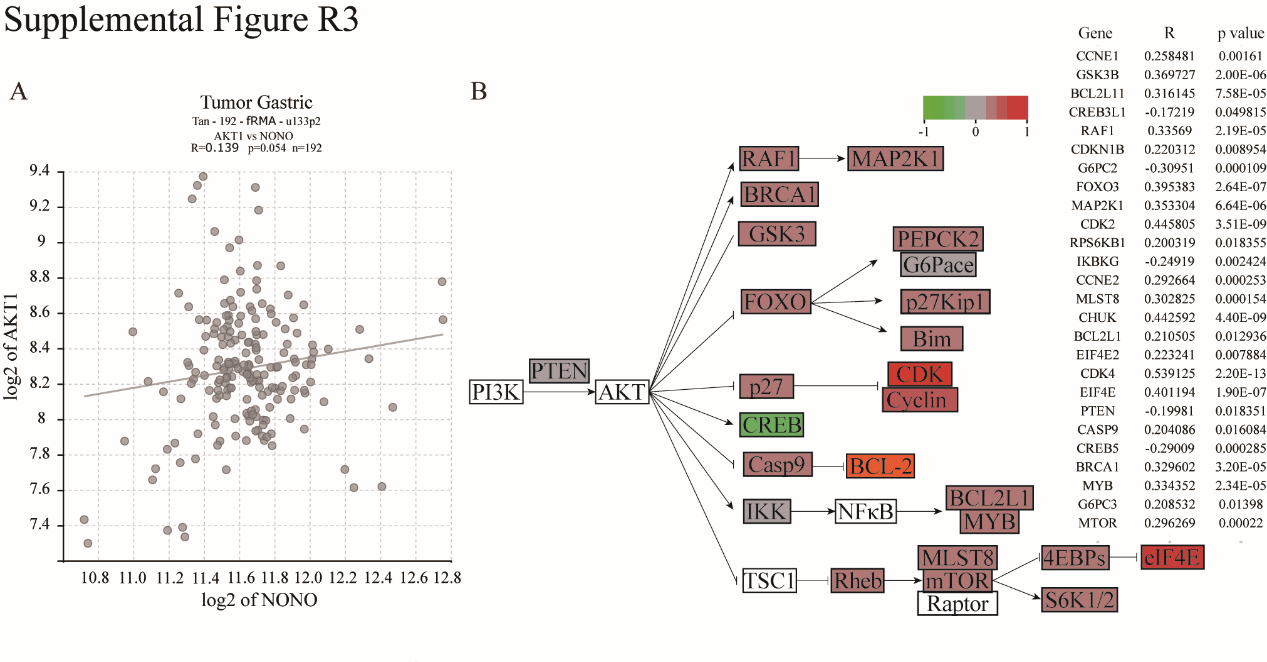


Supplemental Figure R3 **A.** Pearson correlations between the expression of PTEN and NONO in Tumor Gastric Tan-192-fRMA-u133p2. **B.** The downstream of AKT pathway in NONO knockdown GC cells.
